# Lineage specification of human dendritic cell is marked by expression of the transcriptionl factor IRF8 in HSCs and MPPs

**DOI:** 10.1038/ni.3789

**Published:** 2017-06-26

**Authors:** Jaeyop Lee, Yu Jerry Zhou, Wenji Ma, Wanwei Zhang, Arafat Aljoufi, Thomas Luh, Kimberly Lucero, Deguang Liang, Matthew Thomsen, Govind Bhagat, Yufeng Shen, Kang Liu

**Affiliations:** 1 Department of Microbiology and Immunology, Columbia University Medical Center, New York, NY 10032, USA; 2 Departments of Systems Biology and Biomedical Informatics, Columbia University Medical Center, New York, NY 10032, USA; 3 Department of Pathology and Cell Biology, Division of Hematopathology, Columbia University Medical Center, New York, NY 10032, USA

## Abstract

The origin and specification of human dendritic cells (DCs) have not been investigated at clonal level. Using clonal assays combined with statistical computation to quantify the yield of granulocytes, monocytes, lymphocytes and three subsets of DCs from single human CD34^+^ progenitor cells, we show DC lineage specification occurs in parallel with myeloid and lymphoid lineages in HSCs, starting as a lineage bias defined by specific transcriptional programs correlated with the relative IRF8/PU.1 ratios, which is transmitted to most progeny and reinforced by FLT3L-driven IRF8 upregulation over division. We propose a model in which DC lineage specification is driven by parallel and inheritable transcriptional programs in HSCs, and reinforced over cell division by recursive interaction between transcriptional programs and extrinsic signals.

Efforts to construct generally accepted and coherent hierarchical relationships for dendritic cell (DC) development have proven contentious ^1,2, 3,4^. The debate is fueled by the observation that progenitors from either myeloid and lymphoid branches give rise to the same DC subsets ^5, 6^ and by the fact that progenitors defined by the current markers are heterogeneous ^7, 8, 9^. Moreover, most studies have focused on qualitative potency and as such, multipotency has traditionally been interpreted as equipotency ^10^. In addition, suitable ways to quantify, mathematically analyze and identify the significance of potency differentials have not been available. Single-cell RNA-seq and functional clonal analysis have reassessed the homogeneity of progenitor subsets defined by current markers^8, 11, 12, 13^. Single-cell transplantation^14^ and endogenous bar-coding ^15^ has suggested that most mouse myeloid cells derive from HSCs that are restricted to the myeloid lineage, leading to the idea of ‘early imprinting or commitment’ at the HSC stage ^10^. However, human DC lineage specification has not been studied at single-cell resolution. In mouse, *Irf8* expression and function(i.e. driving DC and monocyte development) are thought to occur after the lymphoid-primed multipotent progenitor (LMPP) stage ^16,9, 17^. However, the role and timing of *IRF8* expression and regulation in human DC lineage specification remains unclear.

Here we investigated the developmental potency of human hematopoietic progenitors at the single-cell level and used quantitative analysis of clonal output to investigate the development of granulocyte, monocyte, CD1c^+^ conventional DC (DC1), CD141^+^ conventional DC (DC2), plasmacytoid DC (pDC) and lymphocyte from single cord blood CD34^+^ cells. We found that multipotent progenitors exhibited inherent lineage bias that was established *in vivo* in HSCs, and transmitted to most progeny. The concentration and the relative dosage ratio of PU.1 and IRF8 were highly correlated with specific lineage biases, while FLT3L drove and maintained the DC lineage program over cell division. These results indicate that combinatorial dosage of a common set of transcription factors in HSC-MPPs can shape parallel and inheritable programs for distinct hematopoietic lineages, which are then reinforced through recursive interaction with environmental cytokines.

## Results

### Hematopoietic progenitor subsetss are functionally heterogeneous

**Figure 1:**
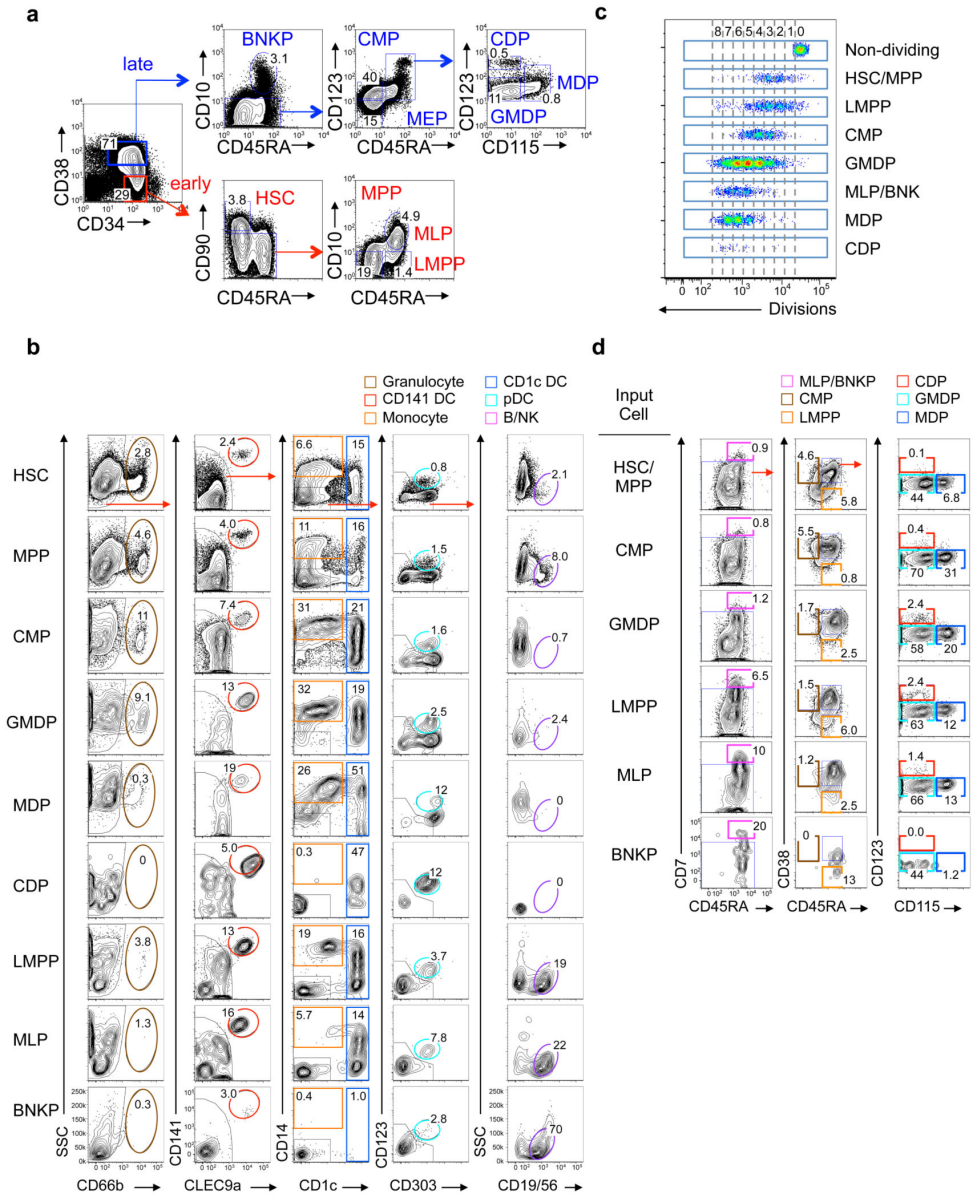
Marker-defined hematopoietic progenitors exhibit hierarchical and convergent potency (**a**) Flow cytometry plot showing gating scheme of progenitor populations from a representative sample of seventeen human cord blood units. Starting gate: Lin(CD3/19/56/14/16/66b/1c/303/141)^-^. BNKP, B/NK progenitor; CMP, common myeloid progenitor; MEP, megaerythrokaryocyte progenitor; GMDP, granulocyte-monocyte-DC progenitor; MDP, monocyte-DC progenitor; CDP, common DC progenitor; HSC, hematopoietic stem cell; MPP, multipotent progenitor; LMPP, lymphoid-primed multi-potent progenitor; MLP, multi-lymphoid progenitor. **(b)** Flow cytometry plots showing output of granulocytes (brown), monocytes (orange), CD1c^+^ DCs (blue), CD141^+^ DCs (red), pDCs (cyan) and B and NK cells (purple) from 100 cells from each indicated population after culturing in MP+FSG condition for 14 days. Shown are live, singlet CD45^+^ cells and numbers indicate mean % of total CD45^+^ cells produced from five independent experiments. **(c)** Concatenated FACS plots showing number of cell divisions (CFSE signal dilution) of indicated populations descended from 1,000 HSCs that were sorted as in **a**, labeled with CFSE and cultured for 7 days. Populations were gated as shown in **d**. Plot is representative of four independent experiments. **(d)** Representative flow cytometry plots showing intermediate output from HSC/MPPs, CMPs, LMPPs, MLPs and BNKPs after culturing 1,000 cells of each population for 7 days. Shown are live, singlet CD45^+^Lin(CD3/19/56/14/16/1c/303/141)^-^CD34^+^ cells. Numbers indicate mean % from total CD34^+^ cells from four independent experiments.

To map the developmental relationship between DC, myeloid and lymphoid lineages, we isolated human CD34^+^ hematopoietic progenitor cells from cord blood and divided them into 10 non-overlapping progenitor populations: CD34^+^CD38^-^CD45RA^-^CD10^-^CD90^+^ HSC, CD34^+^CD38^-^CD45RA^-^CD10^-^CD90^-^ multipotent progenitor (MPP), CD34^+^CD38^-^CD45RA^+^CD10^-^ LMPP, CD34^+^CD38^-^CD45RA^+^CD10^+^ multilymphoid progenitor (MLP), CD34^+^CD38^+^CD45RA^+^CD10^+^ B-NK cell progenitor (BNKP), CD34^+^CD38^+^CD45RA^-^CD10^-^CD123^+^ common myeloid progenitors (CMP), CD34^+^CD38^+^CD45RA^+^CD10^-^CD123^+^CD115^-^ granulocyte-monocyte-DC progenitor (GMDP), CD34^+^CD38^+^CD45RA^+^CD10^-^CD123^+^CD115^+^ monocyte-DC progenitor (MDP), CD34^+^CD38^+^CD45RA^+^CD10^-^CD123^hi^CD115^-^ common DC progenitor (CDP) and CD34^+^CD38^+^CD45RA^-^CD10^-^CD123^-^ megakaryocyte-erythroid progenitor (MEP: used throughout unless otherwise specified) (Table 1, Fig. 1a) ^18, 19, 20, 7^. Because MEPs do not produce DCs, lymphoid or myeloid cells ^18,19^, we evaluated the developmental potential of the other nine progenitor populations into seven mature cell types: granulocytes (G), monocytes (M), lymphocytes (L), specifically B cells (B) and natural killer (NK) cells, and three DC subsets—pDC, DC1, and DC2 using two *in vitro* systems: a colony formation assay for the G, M, megakaryocyte (Mk) and erythrocyte (Er) lineages (Supplementary Fig. 1a) and a culture containing MS5 and OP9 stromal cells, and FLT3L, SCF and GM-CSF cytokines (MP+FSG), to assess G, M, L, A, C and P lineages (see Methods) (Fig. 1b). Due to the lack of NOTCH signaling in the MP+FSG culture, the L lineage is represented only by the output of B and NK cells. As expected, HSCs and MPPs produced all lineages, CMP and GMDP did not produce L cells, while LMPP, MLP and BNKP did not produce Mk/Er cells (Fig. 1b and Supplementary Fig. 1a). However, LMPP and MLP produced G, M and three DC subsets, indicating some myeloid potential (Fig. 1b).

To determine the developmental sequence of the nine progenitor subsets, we cultured carboxyfluorescein succinimidyl ester (CSFE)-labeled HSCs on MP+FSG for 7 days, a period that allows the differentiation of intermediate progenitors ^7, 21^(Fig. 1c and Supplementary Fig. 1b). Various progenitor subsets were observed after a certain number of divisions: CD34^+^CD38^-^CD45RA^+^CD7^-^ LMPPs appeared at division 1–2, CD34^+^CD38^+^CD45RA^-^CD7^-^ CMPs and CD34^+^CD38^+^CD45RA^+^CD7^-^CD123^+^CD115^-^ GMDPs at division 3, CD34^+^CD38^-^CD45RA^+^CD7^+^ BNKPs and CD34^+^CD38^+^CD45RA^+^CD7^-^CD123^+^CD115^+^ MDPs at division 5, and CD34^+^CD38^+^CD45RA^+^CD7^-^CD123^hi^CD115^-^ CDPs at division 7 (Fig. 1c), indicating a hierarchy among progenitor phenotypes. When individual progenitor populations were cultured *in vitro* at 100 cells per well for 7 days, HSCs and MPPs produced both CD34^+^CD38^+^CD45RA^-^CD7^-^ CMPs and CD34^+^CD38^-^CD45RA^+^CD7^-^ LMPPs, CMPs and LMPPs did not differentiate into each other, LMPPs produced MLPs and BNKPs, CMPs produced GMDPs, and GMDPs produced MDPs and CDPs (Fig. 1d and Supplementary Fig. 1c). In addition, MLPs produced GMDPs (Fig. 1d). Similar results were observed at 7 days after 10,000 cells of HSCs and MPPs, CMPs and LMPPs were intratibially transferred into NOD-SCID-IL-2Rγ^null^ (NSG) mice *in vivo* (Supplementary Fig. 1d). These results indicate that MPPs gave rise to CMPs and LMPPs, and that CMP, LMPP and MLP all gave rise to GMDPs (Supplementary Fig. 1e).

**Figure 2:**
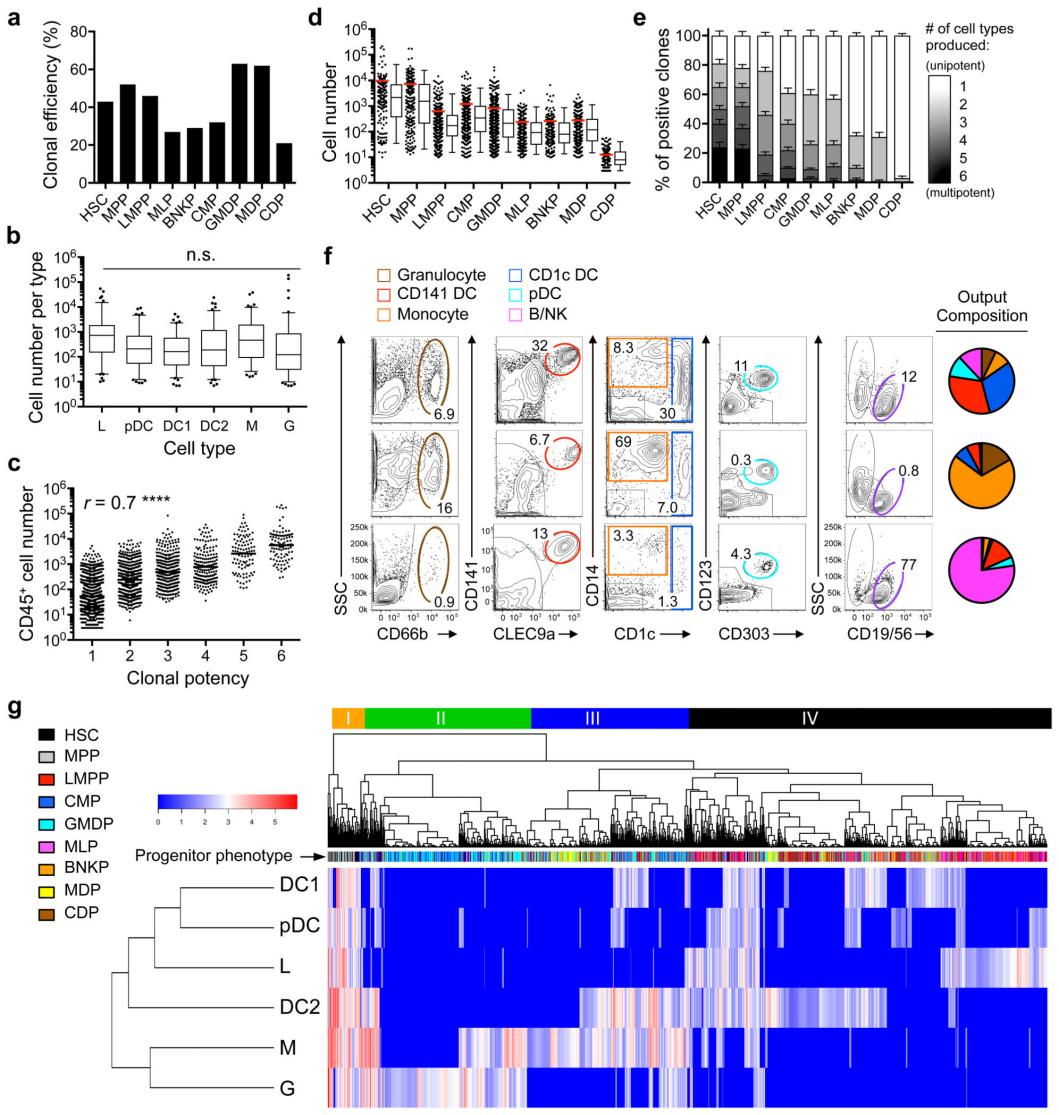
Clonal potency indicates heterogeneity of marker-pure progenitor populations and developmental distance from HSCs **(a)** Bar chart showing clonal efficiency for indicated populations after clonal culture in MP+FSG for 2-3 weeks. Clonal efficiency is defined as percentage of productive clones among total seeded wells for HSC (*n*=360), MPP (*n*=408), LMPP (*n*=791), MLP (*n*=720), BNKP (*n*=542), CMP (*n*=800), GMDP (*n*=890), MDP (*n*=357), and CDP (*n*=691). **(b)** Box-and-whisker plots (center line, median; lower and upper whiskers, 5% and 95% limit, respectively) showing yield of each cell type from all multipotent clones (*n*=105). (L): B/NK cells; (DC1): CD141^+^ DC; (DC2): CD1c^+^ DC; (M): monocyte; (G): granulocyte. Multiple unpaired two-tailed Student's *t*-test, n.s., not significant. **(c)** Scatter plots showing degree of correlation between clonal yield and potential. *r*, Spearman correlation factor; ****, *p* <0.0001. **(d)** Scatter plots and Box-and-whisker plots showing CD45^+^ cell yield of all clones in each population (red lines, mean). **(e)** Stacked columns summarizing qualitative potency of clones from each progenitor population. Bars are standard error of proportion of total positive clones. (**f**) Representative flow cytometry plots showing phenotype of live CD45^+^ cells produced from three individual multipotent clones. Pie charts showing relative abundance of cells for each corresponding clone. **(g)** Heat map showing normalized output for all six mature blood cell types (rows) from each single progenitor cell (columns). Colored bars on the top indicating four major clusters identified by unsupervised hierarchical clustering.

Next we analyzed >5,000 single progenitor cells (referred to as clone hereafter) including HSCs, MPPs, LMPPs, CMPs, MLPs, BNKPs, GMDPs, MDPs and CDPs from human cord blood, of which 2,247 gave rise to progeny in the MP+FSG culture (Fig. 2a). Of the 2,247 clones tested, 105 clones were multipotent and generated all six lineages including L, G, M, DC1, DC2 and pDC, and their average clonal yield of each lineage was statistically indistinguishable, ranging between 620 – 3,465 cells (Fig. 2b), indicating that this culture conditions do not create bias toward any lineage. We divided the 2,247 clones into 6 groups based on the lineage number produced by each clone. The 105 clones that generated six lineages produced the highest number of CD45^+^ progeny, while the 923 unipotent clones produced the lowest numbers of CD45^+^ cells (Fig. 2c), indicating that HSC differentiation correlates with loss of lineage and proliferation potentials. We then compared the clonal (Fig. 2d) and lineage yield (Fig. 2e) of all 2,247 clones grouped via progenitor subsets. Although ranking the progenitor subsets by mean clonal yield correlated with ranking by developmental hierarchy, the yield of individual clones within each progenitor subset varied by orders of magnitude (Fig. 2d and Supplementary Fig. 2a). 24% of HSCs, 23% of MPPs and 0% of all other progenitors produced six lineages; all progenitor subsets displayed marked variation of lineage yield (Fig. 2e and Supplementary Fig. 2b). Notably, although all these progenitors are defined as common progenitors for several lineages ^7, 19^ and thus expected to produce more than one lineage, each population possessed many unipotent progenitors (Fig. 2e and Supplementary Fig. 2b), confirming previous observations ^22^ and suggesting that lineage specification can occur very early. Using flow cytometry to quantify the number of terminally differentiated cells of each lineage (G, M, L, DC1, DC2 and pDC), we observed that the yield of various lineages, or the “quantitative potency” of a given clone, was highly variable (Fig. 2f and Supplementary Table 1), indicating that multipotent progenitors are not equi-potent. Unsupervised hierarchical clustering of 2,247 clones via their quantitative potencies revealed four major clusters that reflected progression of cell development (Fig. 2g). Cluster I comprised highly proliferative and multipotent cells with five- or six-lineage developmental capacity. Clusters II and III consisted of oligopotent and unipotent progenitors with biases toward G and M lineages, respectively. Cluster IV comprised oligopotent and unipotent progenitors that tended to give rise to L, A, P or C lineages (Fig. 2g). All nine progenitor subsets analyzed were highly heterogeneous, and were located in multiple clusters in aggregate (Fig. 2g) or filtered by donor (Supplementary Fig. 2c). As such, progenitor subsets are heterogeneous, but they can be ordered on a differentiation hierarchy based on their proliferation potential.

### Quantitative clonal potency reveals lineage bias in progenitors

**Figure 3:**
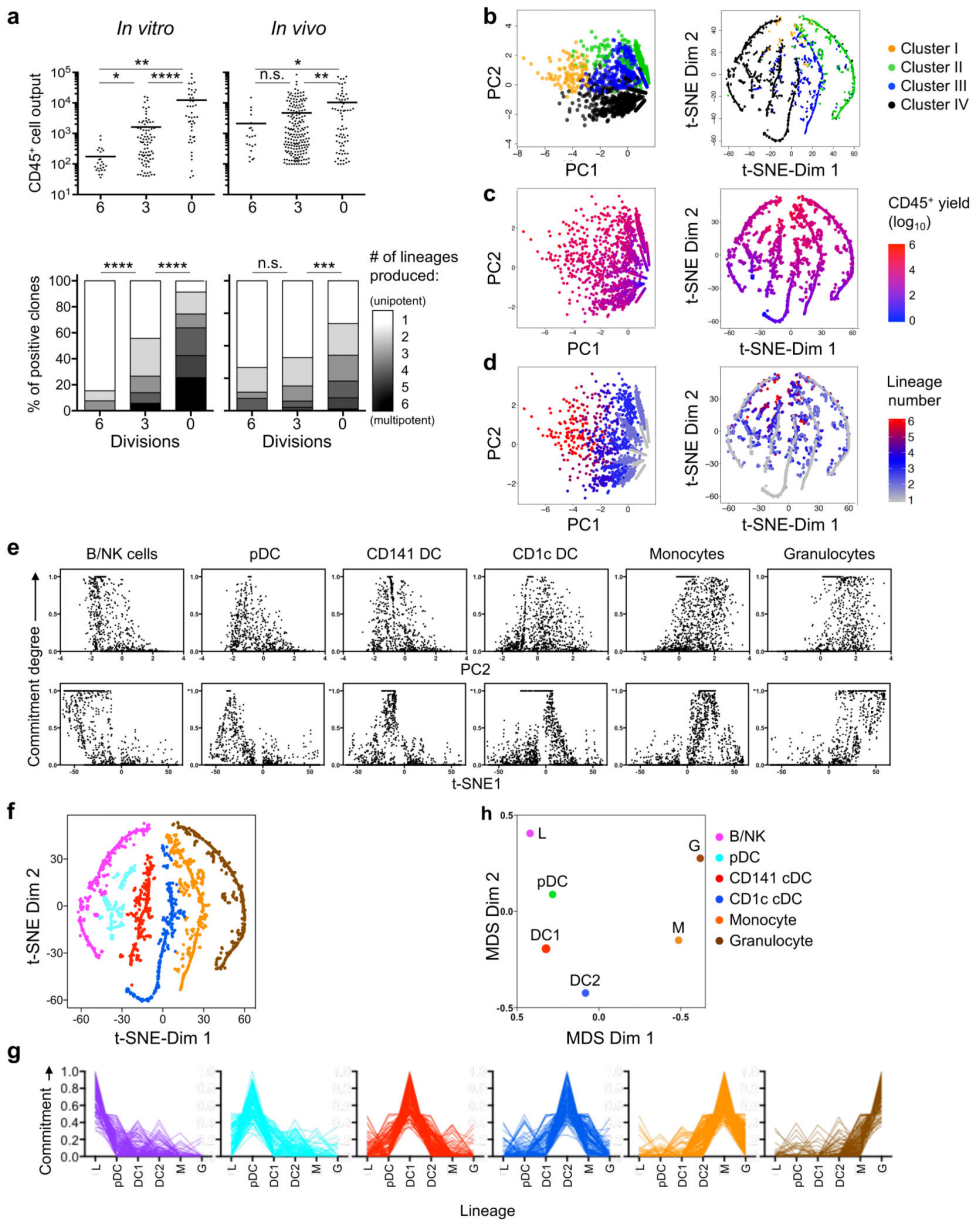
Statistical modeling of clonal potency reveals developmental patterns and lineage biases **(a)** Scatter plots and stacked bar charts showing clonal output and potency composition of CD45^+^ cells derived from division 0, 3, and 6 of CFSE-labeled HSCs, either cultured in MP+FSG (left) or transferred to NSG mice (right) for 6 days. Lines are means and bars s.e.m. **(b-d)** Principal Component Analysis (PCA, left) and t-distributed stochastic neighbor embedding (t-SNE, right) showing clustered clonal data from Fig. 2a, in terms of assigned cluster (**b**), yield **(c)**, and lineages produced **(d)**. **(e)** Plots showing pattern similarities between PCA and t-SNE analysis. Clones were plotted according to their degree of commitment toward a specific cell type (top label) vs. either PCA dimension 2 (upper panels) or t-SNE dimension 1 (lower panels). **(f)** t-SNE map showing each clone assigned to a track based on its shortest distance. **(g)** Lines showing the degree of relative commitment, calculated in terms of offspring composition for each clone (line) on the indicated tracks in **f**. **(h)** Multidimensional scaling (MDS) plot showing developmental relationship among six cell types in terms of the likelihood that two lineages will arise from a common progenitor. *, *p* <0.05; **, *p* <0.01; ***, *p* <0.001; ****, *p* <0.0001; n.s., not significant (**a**: unpaired two-tailed Student's *t*-test, and Fisher's exact test on the frequency of unipotent cells). Data represent cumulative clones from three independent experiments (**a**), or seventeen cord blood donors (**b-h**).

We asked whether quantitative potency could determine each progenitor clone's developmental capacity. CSFE-labeled HSC-MPPs were cultured in MP+FSG or injected intratibially into NSG mice, purified after three or six divisions, corresponding to intermediate or late developmental stages, and evaluated in terms of clonal output (Supplementary Fig. 3a). When total progeny yield and lineage yiled of each clone were compared, HSC-MPPs isolated after three divisions had lower progeny and lineage yields than undivided HSC-MPPs (Fig. 3a), and the magnitude of this decrease was even greater after six total divisions (Fig. 3a), indicating that each clone's quantitative potency inversely correlates with developmental distance from HSCs.

To investigate the developmental relationship between all progenitor clones, we analyzed the similarity of the 2,247 clones as determined by their quantitative potency. Each clone's quantitative potency was described as a six-dimensional vector on its output of each of the six lineages (G, M, L, DC1, DC2 and pDC), and their potency similarity was analyzed using principal component analysis (PCA), which converts data into linearly uncorrelated variables, and t-distributed stochastic neighbor embedding (t-SNE) combined with a Gaussian kernel diffusion model ^23, 24^, which preserves local structure in the multi-dimensional space, to generate two-dimensional maps. Both analyses genererated the same four clusters I-IV (Fig. 3b) that were identified by hierarchical clustering (Fig. 2g), with one dimension correlating with proliferative capacity (Fig. 3c) or number of lineages generated (Fig. 3d), and the other dimension's coordinate correlating with the predominant lineage yield (Fig. 3e). tSNE allows a visualization map in which clones on a given track predominantly generate one lineage, but are ordered in the spectrum from multipotency to unipotency, and from high-yield to low-yield (Fig. 3c,d). As such, progenitor clones that predominantly produce L, pDC, DC1, DC2, M or G cells fall on separate tracks (Fig. 3f and Supplementary Fig. 3a,b), and all clones on a given track have the same lineage bias, producing cells of one lineage in greater numbers than other lineages (Fig. 3f,g). When all clones' quantitative potency was used to compute the degree of ancestry sharing, L and G lineages were considerably less likely to share ancestry than either M and G, or L and pDC (Fig. 3h). Therefore, distances between the lineage tracks reflect the likelihood of “shared ancestry”. Critically, although repeating t-SNE mapping generated different maps, the clustering pattern was highly consistent (Supplementary Fig. 3d). These results indicate that quantitative potency offers a meaningful indicator of a progenitor cell's developmental capacity, allowing grouping of progenitors based on their predominant lineage yield and continuum of yield and lineage restriction.

### Hematopoietic lineage bias starts in HSCs

**Figure 4:**
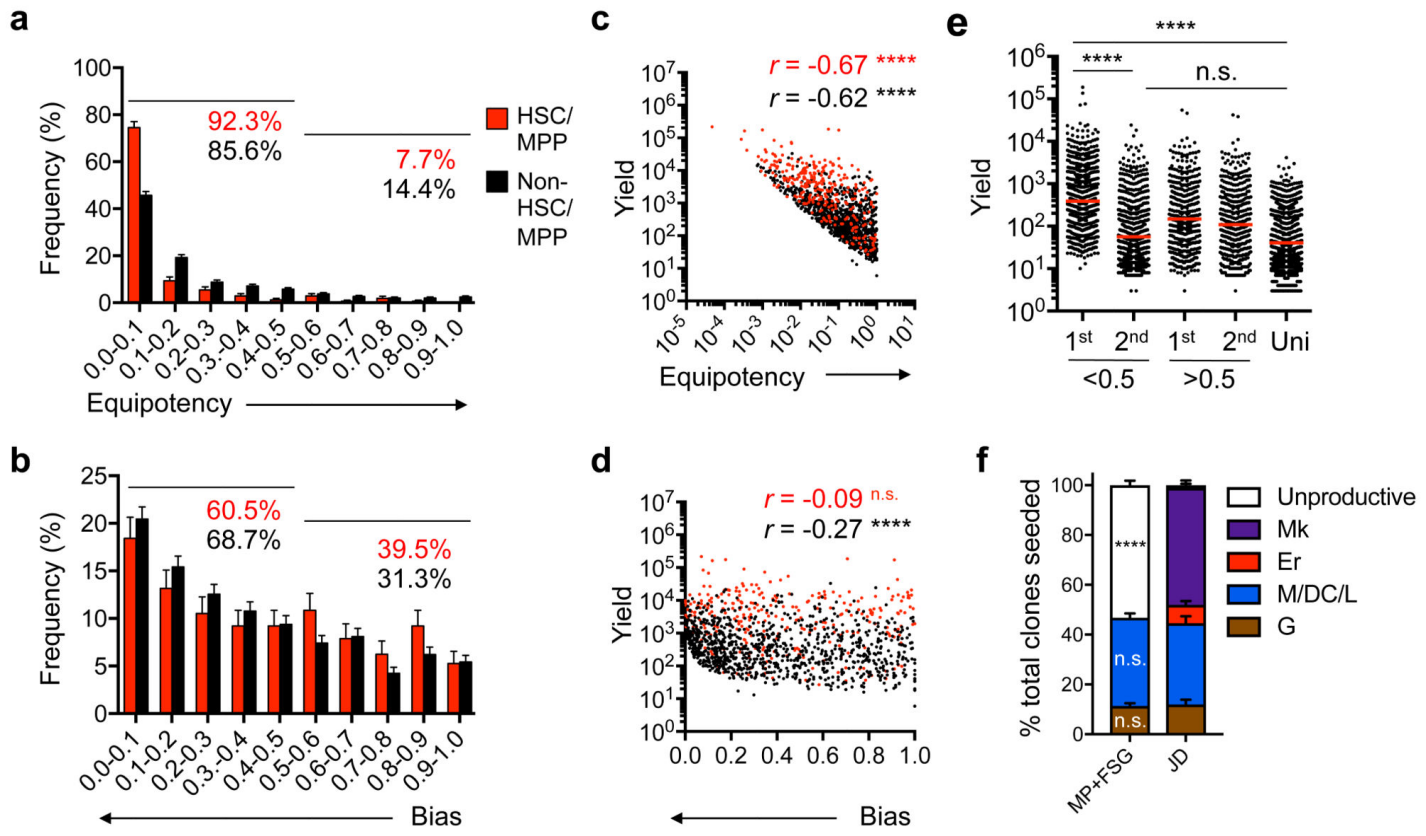
Lineage bias is prevalent and starting early in HSCs (**a-b**) Bar charts showing frequency distribution of all non-unipotent clones based on their degree of equipotency, as determined by the ratio of minimal lineage yield to maximal lineage yield **(a)**; or by their degree of cell-type-specific potency bias, determined by the ratio of second-highest lineage yield to maximal lineage yield (**b**). Numbers indicate the cumulative % of clones for which the ratio is <0.5 (left line) or >0.5 (right line). **(c and d)** Scatter plots showing correlation between equipotency (**c**) or bias degree (**d**) and clonal yield for clones in panel **a** and **b**. *r*, Spearman correlation coefficient. (**e**) Scattered dots showing yield of the largest (1^st^) lineage and second largest (2^nd^) lineage produced by non-unipotent clones whose bias are <0.5 (*n*=115) or >0.5 (*n*=162), and of unipotent progenitors (*n*=931). Red lines are means and bars are s.e.m. (**f**) Stacked bars showing the proportion of HSC/MPP clones that are either unproductive or biased toward erythrocyte (Er), megakaryocytes (Mk), granulocyte (G), or monocyte/DC/lymphocyte (M/DC/L) lineages in MP+FSG (left, n=768) and JD (right, n=286) culture conditions. Bars are standard error of proportion. * *p* <0.05; **** *p* <0.0001; n.s., not significant (**e**: one-way ANOVA; **f**: Fisher's exact test on proportions of productive and non-productive clones, or of M/DC/L and G lineages between the two culture systems). Data represent cumulative clones from seventeen cord blood donors (**a-f**).

To determine whether non-unipotent progenitors are equipotent, as assumed by classical differentiation models, or show lineage bias, we calculated the “equipotency ratio” of all non-unipotent clones by dividing the smallest lineage yield by the largest lineage yield; a ratio of 1 indicates a truly unbiased (i.e. equipotent) clone. Of 1,324 non-unipotent clones, 152 clones had ratios >0.5, and 1,172 had ratios <0.5 (Fig. 4a), indicating that the vast majority of progenitors are not equipotent. Of HSCs and MPPs, 92.3% have ratios <0.5; of all other non-HSC-MPP clones, 85.6% have ratios <0.5 (Fig. 4a), indicating that even HSC and MPP clones are not equipotent. We also calculated the “bias ratio” by dividing the second-largest lineage yield by the maximum lineage yield; a ratio of 0 indicates a wholly biased clone. We observed that 66.7% of non-unipotent progenitors, which included HSCs-MPPs, showed bias ratios < 0.5 (Fig. 4b), indicating lineage bias.

To exclude the possibility that the lineage bias was due to artifacts *in vitro*, we first asked whether the cultured multipotent progenitor clones were initially equipotent and the bias was caused by stochastic progeny death. We plotted bias degree *vs* yield of all non-unipotent clones and observed that highly-biased HSC-MPP or oligopotent progenitors tended to have higher offspring yields (Fig. 4c,d), whereas equipotent progenitors tended to have lower yields (Fig. 4c,d). Because stochastic progeny death would reduce yields, this indicates that lineage bias was not caused by progeny death. Next, to address whether the bias was caused by random lineage expansion during culture, we compared the largest lineage yield from 878 biased progenitor clones that produced a single major lineage (bias ratio <0.5) with the largest lineage yield from 438 unbiased clones with two major lineages (bias ratio >0.5) (Fig. 4e). The largest lineage yields of biased clones was significantly higher than those of unbiased clones (Fig. 4e), indicating that lineage bias was the product of neither stochastic death nor random lineage expansion *in vitro*, but rather *in vivo* establishment prior to isolation and culture, and it is intrinsically correlated with proliferative capacity.

To address whether the lineage bias is caused by media microenvironment, we compared the clonal composition of HSC-MPPs in MP+FSG culture and in a different culture system using MS5 stromal cells with SCF, FLT3, TPO, EPO, IL-6, IL-3, IL-11 and GM-CSF cytokines (JD culture here after), which supports differentiation of Er and Mk lineages in addition to the G, M, DC and L lineages ^22^ (Supplementary Fig. 4a,b). In terms of clonal efficiency, 52% of total HCS-MPP clones were unproductive in MP+FSG, while 2% of HCS-MPP were unproductive in the JD culture (Fig. 4f), indicating that HSCs and MPPs were neither totipotent nor equipotent, as totipotent and equipotent HSC-MPPs should expand to display comparable clonal efficiency in either culture system. The clonal composition of G and M-DC-L lineages was 46.75% in MP+FSG and 44.56% in JD cultures (Fig. 4f), indicating that culture conditions do not induce lineage biases based on cytokine composition and concentration, which are different in the two cultures. About 5% of HSC-MPP clones produced all lineages in JD system, versus ∼11% in MP+FSG system (Supplementary Fig. 4c), suggesting they were totipotent progenitors. On average, totipotent HSC-MPPs were more proliferative than non-totipotent HSC-MPPs (Supplementary Fig. 4d). However, the HSC-MPPs with the highest clonal yield were not totipotent (Supplementary Fig. 4d). In addition, the totipotent HSC-MPP clones were not equipotent and exhibited lineage bias, like the non-totipotent clones, in both MP+FSG and JD cultures (Supplementary Fig. 4e,f). These observations indicate that multipotent progenitors were not equipotent, that most progenitors—including rare totipotent clones—had an inherent lineage bias that was established *in vivo* early in HSPs, and that there is a correlation between lineage bias and the proliferative capacity.

### Lineage bias is heritable and transmitted to progeny

**Figure 5:**
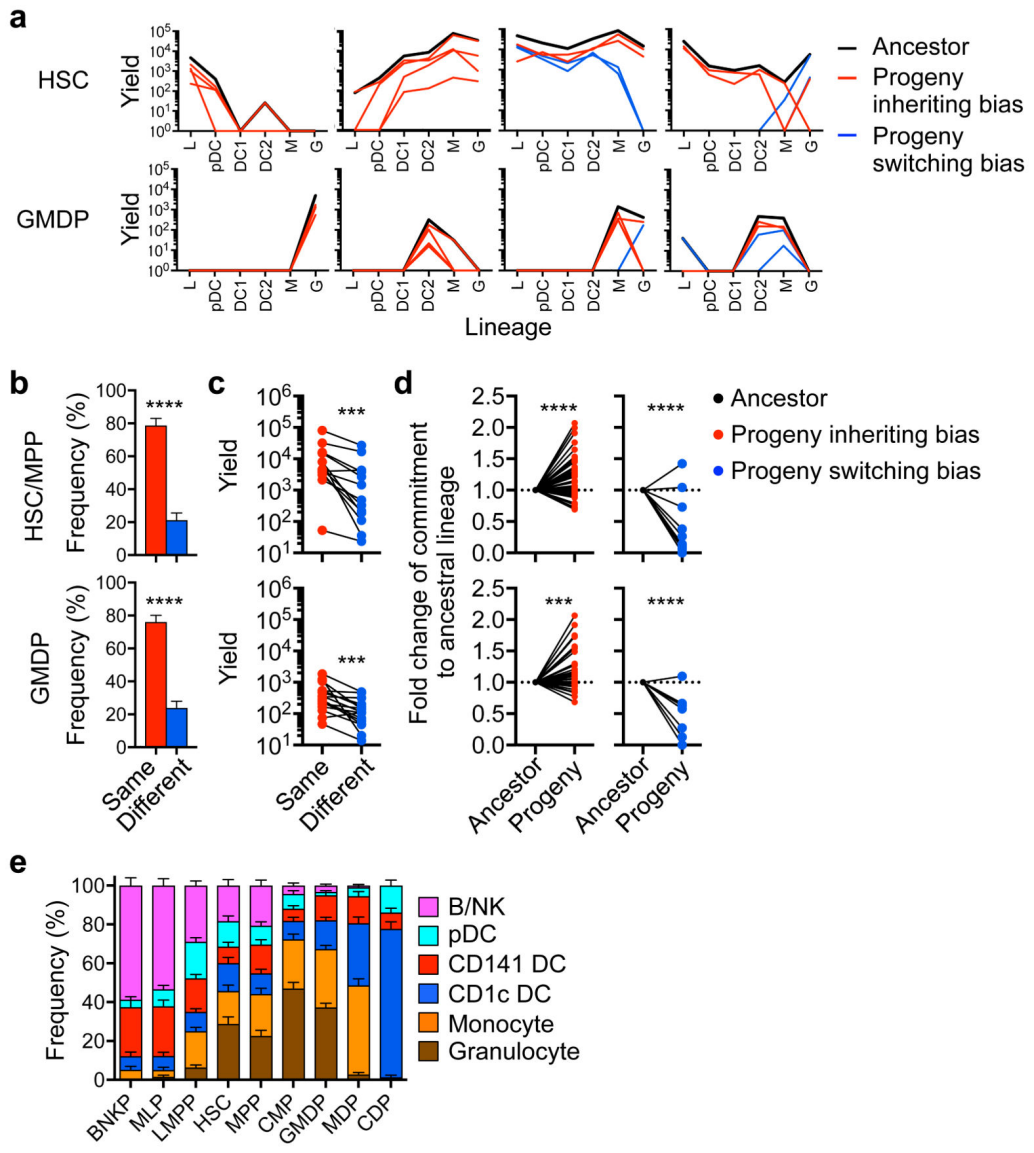
Lineage bias is transmitted to most progeny and can be further amplified toward full commitment along division (**a**) Lines showing the yield for six lineages from ancestors (black) and progeny with potency profiles that are similar (red) or different (blue) from the ancestral clone. **(b)** Bar graphs showing the frequency of granddaughter cells that have inherited (red) or switched (blue) their ancestor's lineage bias (HSC, *n*=89; GMDP, *n*=109). **(c)** Plots comparing the average yield of siblings with inherited (red) or switched (blue) bias (HSC, *n*=13; GMDP, *n*=16). (**d**) Fold-change of commitment degree in progeny that inherited (left; HSC, *n*=66; GMDP, *n*=47) or switched (right; HSC, *n*=19; GMDP, *n*=26) from their ancestor's lineage bias. **(e)** Stacked columns showing relative composition of clonal bias for progenitor subtypes isolated from cord blood. Bars, mean values; error bars, standard error of proportion (calculated from total number of positive clones for each progenitor). ***, *p* <0.001; ****, *p* <0.0001 (**b**: unpaired two-tail Student's *t*-test; **c-d**: paired two-tail Student's *t*-test). Data shown are representative of cumulative clones from three (**a-d**) or from seventeen cord blood donors (**e**).

To evaluate if lineage bias is maintained through progenitor cell differentiation into their progeny, single HSCs and GMDPs labeled with the fluorescent dye DiD were cultured in MP+FSG for 2–4 days, and each of the four granddaughter cells was individually cultured for 2 more weeks (Supplementary Fig. 5a). We measured each granddaughter's quantitative potency and inferred each ancestor's quantitative potency as the sum of its granddaughters'. Tracing 198 granddaughter cells showed that the majority of HSCs' and GMDPs' progeny produced the same predominant lineage as their ancestor, suggesting lineage inheritance, although some progeny produced a different predominant lineage from their ancestor, suggesting bias “switching” (Fig. 5a and Supplementary Fig. 5b, c). To quantify the relative rate of bias inheritance and bias switching, we compared the lineage bias of each granddaughter cell to that of its ancestor. Notably, 79.6% of HSC progeny and 76.1% of GMDP progeny inherited ancestral bias, and 20.4% of HSC progeny and 23.9% of GMDP progeny switched bias to a different lineage (Fig. 5b), indicating that the majority of progeny inherited ancestral bias. We then compared the clonal yield of the bias-inheriting progeny with their bias-switching siblings. The clonal yields of bias-inheriting progeny was significantly higher than those of bias-switching progeny (Fig. 5c). There was a significantly higher degree of commitment among all bias-inheriting progeny than bias-switching ones (Fig. 5d), indicating that bias-inheriting progeny amplified their inherited bias. For bias-switching progenies, there was considerable flexibility in the bias-switching direction, such that ancestors biased towards G, M, DC1, DC2, pDC or L lineages could give rise to progeny with any other lineage bias (Supplementary Fig. 5b, c). However, bias switches in GMDP progeny were more likely to occur between G and M, M and DC2, or DC1 and DC2, while HSC progeny could switch between G and L (Supplementary Fig. 5b, c), indicating a greater degree of bias switch flexibility in HSC than GMDP.

Next, we calculated the frequency of clones biased toward different lineages in each of the marker-defined progenitor populations. Each population was comprised of groups of clones biased toward distinct lineages, and the proportion of these lineage groups was distinct and characteristic for each population analyzed (Fig. 5e). On the t-SNE visualization map, clones within marker-defined progenitor populations were distributed across multiple tracks of distinct lineage bias (Supplementary Fig. 5d), indicating that clones attributed to each progenitor population by markers can fall on either tracks consistent with the unique transcriptional patterns described by single-cell RNA-seq in mouse CMPs and GMPs ^13^. We conclude that the hematopoietic lineage bias is established *in vivo* in HSCs, heritable and amplified during proliferation.

### IRF8 expression marks CD141 DC lineage specification in HSCs and MPPs

**Figure 6:**
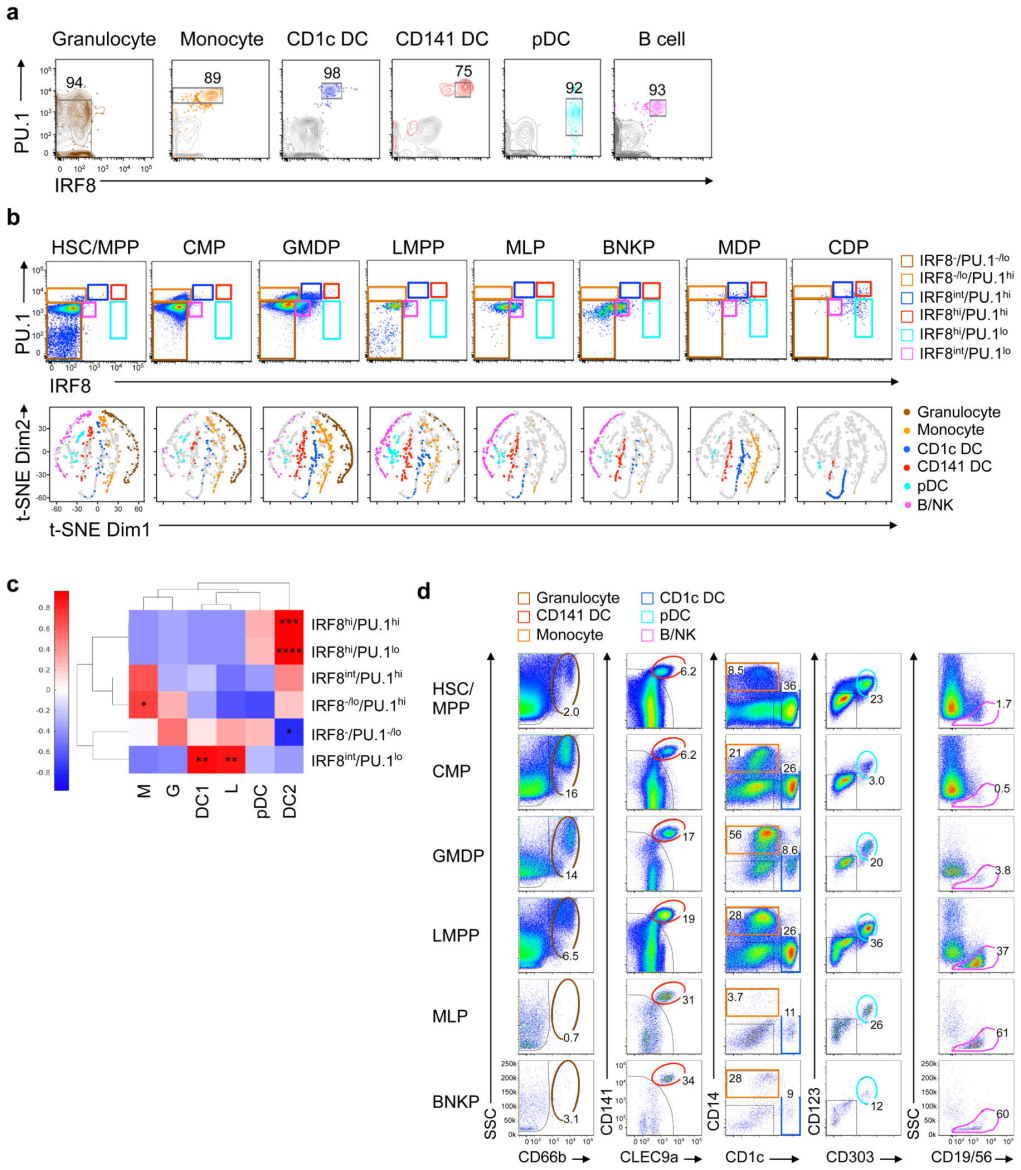
Distinct and inheritable pattern of IRF8 and PU.1 expression in progenitors correlates with lineage bias **(a-b)** Flow cytometry plots showing protein levels of IRF8 and PU.1 in six types of mature immune cells **(a)** and nine types of progenitors (**b, top**). Boxes correspond to the gates for relative IRF8 and PU.1 expression level in each of the six mature cell lineages. t-SNE maps in **b (bottom)** show distribution of clones derived from progenitors, with colors indicating their corresponding lineage biases. (**c**) Heat map showing Pearson correlation coefficients between percentages of subpopulations identified by IRF8/PU.1 dosage and percentages of lineage composition of different progenitors as determined for **b**. The correlation matrices were hierarchically clustered and are shown in the heat map. **(d)** Flow cytometry plots showing *in vivo* potency of HSC/MPP, CMP, GMDP, LMPP, MLP and BNKP populations in NSG-SGM3 mice 14 days after intratibial injection. Numbers show percentages of parental gate. * *p* <0.05; ** *p* <0.01; *** *p* <0.001; **** *p* <0.0001 (**c**: paired two-tail Student's *t*-test for transformed correlation). Data shown are representative of three independent experiments (**a-d**).

We next investigated the transcriptional program associated with lineage bias in HSC. The transcription factors IRF8 and PU.1 are important for development of multiple blood lineages, including DC subsets ^25, 26, 27^. Because in mice, Pu.1 controls *Irf8* expression^17^*,* which prevents neutrophil development in MDP and common monocytic progenitor (cMOP) ^16, 17, 28^ and regulates the survival and function of terminally differentiated DC1s and pDCs ^9^ we examined the protein expression kinetics of IRF8 and PU.1 during human DC hematopoiesis by intracellular staining. We found distinct concentrations and ratios of IRF8 and PU.1 (IRF8-PU.1 dosage hereafter) in differentiated G (IRF8^-^/PU.1^lo^), M (IRF8^lo-int^/PU.1^hi^), L (IRF8^int^/PU.1^lo^), DC1 (IRF8^hi^/PU.1^hi^), DC2 (IRF8^int^/PU.1^hi^) and pDC (IRF8^hi^/PU.1^lo^) cells (Fig. 6a). IRF8 protein was high in pDCs and DC1 compared to DC2 and other cells (Fig. 6a). IRF8 and PU.1 were detectable as early as the HSC and MPP stages, albeit in a small number of cells (Fig. 6b), while MLP, BNKP, CMP, MDP and CDP could be divided into sub-populations with distinct dosage combinations of IRF8 and PU.1 (Fig. 6b), reminiscent of those seen in mature L, pDC, DC1, DC2, M and G cells (Fig. 6a). The IRF8^int^PU.1^lo^ subpopulation was prominent among LMPPs, MLPs and BNKPs, whereas the IRF8^int^PU.1^hi^ subpopulation was abundant among GMDP and MDPs (Fig. 6b). To examine the correlation between the percentages of subpopulations identified by IRF8-PU.1 dosage and percentages of clones biased to L, G, M, DC1, DC2 and pDC lineages in different progenitor populations measured by clonal assay in MP+FSG (Fig. 5e), we calculated Pearson correlation coefficients between the percentages in all tested populations. There was a positive correlation between the IRF8^int^PU.1^lo^ subpopulation and DC1 lineage (r=0.91), and between the IRF8^int^PU.1^hi^ subpopulation and DC2 (r=0.46) and M (r=0.64) lineages (Fig. 6c), suggesting the propensity of IRF8^int^PU.1^lo^ to produce DC1 and IRF8^int^PU.1^hi^ cells to produce DC2 and M. To test the relevance of IRF8 and PU.1 expression in terms of DC subset potency *in vivo*, we purified HSC-MPP, MLP, BNKP, LMPP, GMDP and CMP from cord blood and injected them intratibially into NSG-SGM3 mice. Two weeks after the transfer, CMPs and GMDPs, which are predominantly IRF8^int^PU.1^hi^ produced abundant G, M and DC2, but fewer DC1 and pDC (Fig. 6d). In contrast, LMPPs, MLPs and BNKPs, which are predominantly IRF8^int^PU.1^lo^ produced abundant L, DC1 and pDC, but few DC2 and M (Fig. 6d), indicating that the progenitor cells' IRF8-PU.1 dosages correlate with certain biases towards distinct DC subsets.

**Figure 7:**
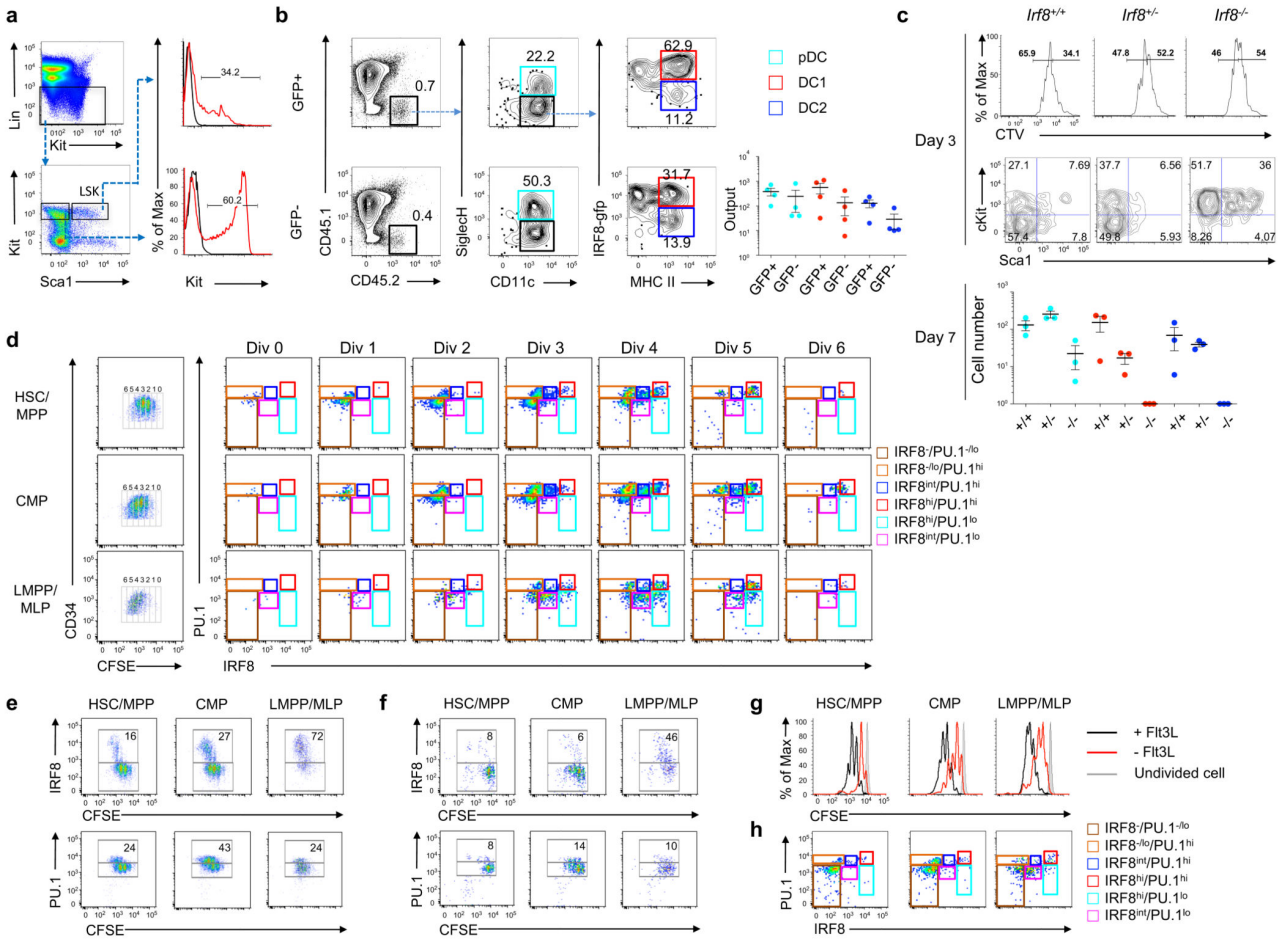
Early expression of IRF8 in HSCs facilitates the specification of DC1 lineage (**a**) Flow cytometry plots and histograms showing gating scheme of bone marrow Lin^-^Sca^+^Kit^+^ (LSK) cells from B6 and *Irf8^gfp^* mice. (**b**) FACs plots showing production of differentiated DCs from GFP^+^ and GFP^-^ LSK cells after 7 days of co-culture with CD45.1 bone marrow cells with Flt3L. Scatter plots (right) showing yield of differentiated DCs. Bars are mean + s.e.m. (**c**) Histogram showing proliferation (top) and dot plots showing Sca1 and cKit phenotype of LSK cells from *Irf8^+/+^*, *Irf8^+/-^*, *Irf8^-/-^* mice after 3 days of culture as in **a**. Scatter plot showing yield of differentiated DCs after 7 days of culture. Bars are mean ± s.e.m. (**d**) FACs plots showing CFSE dilution of CB-derived HSC/MPPs, CMPs, and LMPP/MLPs after 6 days of MP+FSG culture (left). Numbers indicate rounds of divisions. Remaining panels (right) show protein levels of IRF8 and PU.1 per division. (**e-f**) FACs plots showing expression of IRF8 (top) and PU.1 (bottom) along division after 6 days of MP+FSG (**e**) or MP+SG culture (**f**). Numbers indicate percentage of IRF8^+^ (top) and PU.1^+^ cells (bottom). (**g**) Histogram showing division of indicated progenitors in MP+FSG or MP+SG culture. (**h**) Plots showing IRF8 and PU.1 expression for each progenitor after six days in MP+SG culture. Data are representative from four (**a**) and three (**d-h**) independent experiments, or from four (**b**) and three (**c**) mice per group.

To test whether the expression of Irf8 may mark DC lineage specification at the HSC-MPP stage in mice, we used Irf8^gfp/gfp^ mice that have eGFP fused to the C terminus of the endogenous *Irf8*^29^. About 34% of Lin^-^Sca^+^Kit^+^ (LSK) cells, which include HSCs, MPPs and LMPPs, in Irf8^gfp/gfp^ mice expressed intermediate level of GFP (Fig. 7a), correlating with intracellular antibody staining of IRF8 (data not shown). Same numbers of GFP^+^ and GFP^-^ LSKs from Irf8^gfp/gfp^ mice were seeded in a culture containing Flt3L cytokine, which supports differentiation of DC1, DC2 and pDC ^30^. GFP^+^ LSKs produced 4 fold more DC1s and DC2s than GFP^-^ LSKs from the same mice, although their pDC output was similar (Fig. 7b), indicating that IRF8 expression in LSKs can distinguish subpopulations with distinct DC subset potency.

To analyze the dosage effect of *Irf8*, we used mice carrying different number of *Irf8^-^* alleles generated by crossing of B6(Cg)-*Irf8*^tm1.1Hm^/J (*Irf8^f/f^*) with *Sox2-Cre* mice. We isolated LSK cells from *Irf8^+/+^, Irf8^+/-^* and *Irf8^-/-^* mice, labeled them with CellTrace Violet (CTV) and investigated their proliferation and DC development in the Flt3L culture as above. On day 3 of culture, *Irf8^+/-^* and *Irf8^-/-^* LSKs showed reduced proliferation compared to *Irf8^+/+^* LSK (Fig. 7c). Moreover, *Irf8^-/-^* LSKs maintained high expression of Sca-1 and Kit in comparison to *Irf8^+/-^* and *Irf8^-/-^* LSKs (Fig. 7c), indicating that IRF8 deficiency impaired LSK differentiation. On day 7, *Irf8^+/-^* LSKs produced 9 fold fewer DC1s and 2 fold fewer DC2s compared to *Irf8^+/+^* LSKs, while *Irf8^-/-^* LSKs failed to produce any DC1s and DC2s (Fig. 7c). pDCs did not develop from Irf8^-/-^ LSKs, but developed normally from *Irf8^+/-^* LSKs (Fig. 7c). These data indicate that Irf8 functionally regulates the proliferation and specification of DC subset lineages in a dose-dependent manner at around the HSC stage.

To trace the development of human IRF8^int^PU.1^lo^ DC progenitors into IRF8^hi^PU.1^hi^ CD141^+^ DC1s, we purified HSCs, CMPs and LMPPs from cord blood, labeled them with CFSE and assessed the change of PU.1 and IRF8 expression over several cell divisions in MP+FSG culture. Few LMPP progeny were IRF8^int^PU.1^hi^ throughout all divisions, while the IRF8^int^PU.1^lo^ LMPP progeny expanded and peaked at division 3-4 (Fig. 7d), followed by an increase in the number of IRF8^hi^PU.1^hi^ cells at division 4-5 (Fig. 7d), suggesting that the initial IRF8^int^PU.1^lo^ expression profile of LMPPs was transmitted to most progeny and further reinforced during cell division to establish a bias towards commitment to CD141 DCs. Both IRF8 and PU.1 expression increased over the course of LMPP division (Fig. 7d), but while IRF8 expression increased rapidly over the course of cell division, PU.1 expression remained relatively low and increased at a considerably slower rate (Fig. 7d). This suggests that IRF8^int^PU.1^lo^ cells rapidly increases IRF8 expression over cell division and give rise to IRF8^hi^PU.1^hi^ cells.

Because cell division is driven by extrinsic cytokines, we examined the role of extrinsic cytokines in strengthening lineage identity by assessing the effect of withdrawing FLT3L, the key cytokine that regulates DC development *in vivo*. We cultured CFSE-labeled HSCs, CMPs and LMPP-MLPs a culture containing MS5, OP9 stromal cells and SCF and GM-CSF cytokines without Flt3L (MP+SG hereafter). Compared to MP+FSG culture, HSC-MPPs, CMPs and to a lesser degree LMPPs showed reduced division in MP+SG (Fig. 7e-g), and few of the cells that underwent division upregulated IRF8 (Fig. 7f), resulting in a significantly lower generation of IRF8^hi^PU.1^hi^ cells (Fig. 7h), which were associated with development of CD141^+^ DC, indicating that FLT3L not only facilitated division of early progenitors, but also drove IRF8 expression, maintenance and upregulation. Altogether, these data indicate that IRF8-PU.1 dosage correlates with lineage bias established in HSCs (Supplementary Fig. 6a), that IRF8 expression starts as early as in HSCs, where it regulates propagation of LSKs and their development into DCs, and that the maintenance and reinforcement of IRF8 expression over the course of cell division was dependent on FLT3L (Supplementary Fig. 6b).

## Discussion

Here we show that human DC lineage specification occurs in parallel with myeloid and lymphoid lineages in HSCs, and is defined by specific transcriptional programs correlated with the relative IRF8/PU.1 ratios. IRF8 expression in HSC-MPPs facilitated propagation of DC progenitors and was driven by FLT3L over cell division.

Previous single cell studies suggested early myeloid and DC lineage specification in mouse^11, 14, 15^, and divergence of erythro-megakaryocytic lineage from HSC-MPPs in humans ^22^. We showed that in HSC-MPPs lineage specification started as a bias, which was heritable and transmitted to most progeny, where it was further amplified and reinforced toward commitment over cell division. Consistent with this, the proportion of Er- and Mk-biased HSC-MPPs in JD (i.e. Er- and Mk-supportive) culture corresponds with the proportion of unproductive HSC-MPPs in MP+FSG (i.e. Er- and Mk-unsupportive) culture. Our granddaughter-tracing experiment suggested that ancestor cells can generate progeny that switch lineage biases, which would explain previous interpretations as a series of binary choices events in multipotent progenitors. However, most progeny inherited the ancestral lineage bias, whereas bias-switching happened infrequently, and these progeny tended to be less proliferative. Thus, we estimate that the majority of mature blood cells are produced from lineage-specified, long-term progenitors that proliferate and transmit their lineage bias to their progeny, while bias switching contributes minimally to the overall production of mature blood cells. This is consistent with that most mature blood myeloid cells descend from both myeloid-restricted HSCs progenitors ^15^.

Progenitor subsets contained clones with various dosage combinations of IRF8 and PU.1, which correlated with the clonal lineage biases. This is consistent with the reported dosage-dependent roles of IRF8 and PU.1 in regulating development of DCs, monocytes and B cells ^26, 31,32,33^ and could explain the heterogeneity of progenitor subsets reported in many studies ^8, 11, 12, 13^.

We observed IRF8 expression in HSCs with low PU.1 expression, and that IRF8 expression rapidly increased in HSC, CMP and LMPP progeny, consistent with the idea that *Irf8* transcription depends on Pu.1 ^17^ and auto-activation ^9^. Due to Irf8's low affinity for interferon response elements, it must be recruited to DNA through interactions with Pu.1 or Batf (AP-1) ^34, 35^. In mouse MDPs, Pu.1 binds a distal enhancer of *Irf8* to drive its transcription^17^. Later in pre-cDCs, Irf8 binds its own enhancer to reinforce its own transcription, thereby reinforcing CD8^+^ DC1 commitment^9^. E2-2 employs similar autoactivation to reinforce the pDC lineage program^36^. IRF8 expression increased sharply within human LMPP progeny despite relatively low PU.1 protein. Given that different Irf8 enhancers are activated in mouse MDPs, DC1s and pDCs^9, 17^, an alternative enhancer may facilitate *IRF8* transcription in human LMPPs.

FLT3L drove both division of early progenitors and IRF8 upregulation over cell divisions, consistent with a Flt3L requirement for mouse DC development ^37^. Lineage bias in HSCs was transmitted and further amplified over cell divisions, and that cell division was coupled with the sequential acquisition of progenitor phenotypes, as defined by the expression of cell surface receptors including CD38, CD45RA, FLT3(CD135), CD115, CD10 and CD123^7, 18^. Although receptor expression phenotype does not equalize or synchronize with the transcriptional program, both can be linked with extrinsic signals and cell division. We speculate that combinatorial dosage of a common set of transcription factors in HSC/MPPs can shape parallel and inheritable programs for distinct hematopoietic lineages, which are then reinforced over cell division by recursive interaction between transcriptional programs and extrinsic signals.

## Methods

### Human samples

Human umbilical cord blood was purchased from New York Blood Center (New York) and processed 24-48hrs post-delivery. Human bone marrow was obtained from the Hematopathology Division or the Columbia Center for Translational Immunology at Columbia University Medical Center (New York). Informed consent was obtained from the patients and/or exempt from informed consent being residual material after diagnosis and fully de-identified. All samples were collected according to protocols approved by the Institutional Review Board at Columbia University Medical Center.

### Mice

NOD.Cg-Prkdcscid-IL2rg*^tmlWjl^*/Sz (NOD/SCID/IL2rγ^null^ or NSG) mice and NOD.Cg-Prkdcscid-IL2rg*^tmlWjl^* Tg(CMV-IL3,CSF2,KITLG)1Eav/MloySzJ (NSG-SGM3), C57BL/6J, CD45.1, *Irf8*^-/-^ (stock number 018298) mice and IRF8^gfp^ reporter mice (stock number 027084) were purchased from Jackson Laboratory and bred in pathogen free animal facility at CUMC. *Irf8*^+/−^ mice were obtained by crossing of *Irf8*^−/−^ mice to wild-type C57BL/6J mice. All experiments were performed according to the guidelines of IACUC at CUMC. For experiments, both sex of mice between 4-8 weeks were used.

### Cell isolation and flow cytometry

Fresh mononuclear cells were isolated from cord blood or bone marrow by density centrifugation using Ficoll-Hypaque (Amersham Pharmacia Biotech, Piscataway, NJ). Samples were incubated with fluorescent-labeled antibodies for direct analysis on the BD LSR II flow cytometers (Becton Dickinson Immunocytometry Systems [BDIS], San Jose, CA) or further purification by fluorescence-activated cell sorting on the BD Influx or BD FACSAria, both using HeNe and argon lasers. Sorted population showed >95% purity.

For human hematopoietic progenitor cell analysis, single cell lineage potential, developmental hierarchy relationship experiments, daughter cell lineage potential, and characterization of progenitor cells from normal and patient BM, CD34^+^ cells were first enriched from cord blood or bone marrow using CD34 MicroBead Kit and LS MACS magnetic columns (Miltenyi Biotec, Auburn, CA). Enriched CD34^+^ cells (70%-95% purity) were incubated with antibodies against CD3 (OKT3, Brilliant Violet (BV) 650, Biolegend), CD19 (HIB19, BV650, Biolegend), CD56 (HCD56, BV650, Biolegend),CD14 (TuK4, Qdot-655, Invitrogen), CD66b (G10F5, PerCP-Cy5.5, Biolegend), CD303 (201A, PerCP-Cy5.5, Biolegend), CD141 (M80, PerCP-Cy5.5, Biolegend), CD1c (L161, APC-Cy7, Biolegend), CD34 (581, Alexa Fluor (AF) 700, Biolegend), CD38 (HIT2, BV421, Biolegend), CD90 (5E10, PE, Biolegend), CD45RA (HI100, AF488, Biolegend), CD123 (6H6, BV510, BD), CD10 (HI10a, PE-Cy7, Biolegend), CD115 (9-4D2-1E4, APC, Biolegend). For culture experiments, progenitors were sorted from Lin(CD3/19/56/14/66b/303/141/1c)^-^cells and following Table 1 surface phenotypes.

For inter-developmental relationship experiments, cells from either culture or NSG bone marrow were stained for LIVE/DEAD® (Life technologies), CD45 (HI30, AF700, Biolegend), CD14 (Qdot-655), CD3 (OKT3, BV650, Biolegend), CD19 (HIB19, BV650, Biolegend), CD56 (HCD56, BV650, Biolegend), CD16 (3G8, BV650, Biolegend), CD11c (3.9, PerCP-Cy5.5, Biolegend), CD66b (PerCP-Cy5.5), CD303 (PerCP-Cy5.5), CD141 (PerCP-Cy5.5), CD34 (581, APC-Cy7, Biolegend), CD38 (BV421), CD90 (PE), CD7 (CD7-6B7, PE-Cy7, Biolegend), CD45RA (AF488), CD123 (BV510) and CD115 (APC). For *in vivo* transfer experiments, mouse CD45 (30-F11, PB, BD) was also stained.

For characterization of terminally differentiated cells in single cell cultures or NSG bone marrow, cells were stained with LIVE/DEAD® (Life technologies), CD45 (AF700), CD66b (PerCP-Cy5.5), CD56 (B159, Pacific Blue (PB), BD), CD19 (HIB19, PB, Biolegend), CD14 (Qdot-655), CLEC9a (8F9, PE, Biolegend), CD1c (L161, PE-Cy7, Biolegend), CD303 (201A, FITC, Biolegend), CD123 (6H6, Brilliant Violet (BV) 510, Biolegend), CD141 (AD5-14H12, APC, Miltenyi), CD235a (GA-R2, APC, BD Pharmingen), CD41a (HIP8, APC-H7, BD Pharmingen) for 40 minutes on ice. 4 or 10ul of antibody mix was used to stain cells harvested from 96- or 24-well plates, respectively. For *in vivo* transfer experiments, mouse CD45 (30-F11, PB, BD) was also stained.

For intracellular staining of PU.1 and IRF8, cells were first stained with antibodies against surface markers, fixed and permeablized using the Foxp3 Fixation/Permeabilization Concentrate and Diluent Kit (eBioscience) for 20 minutes on ice, and then stained with antibody against IRF8 (V3GYWCH, PE, eBioscience) and PU.1 (7C6B05, AF647, Biolegend) in 1× Permeabilization buffer (eBioscience) for more than one hour on ice.

Differentiated DCs from mouse bone marrow progenitor cells were identified by CD45.2 (clone number, company), CD45.1 (clone number, company), CD11c (clone number, company), I-Ab (clone number, company), SiglecH (clone number, company), CD172a (clone number, company).

### Cell culture

Two culture system were used for cord blood derived progenitor clonal assay. For MP+FSG culture, MS5 and OP9 stromal cells were maintained and passed in complete alpha MEM medium (Invitrogen) with 10% FCS and penicillin/streptomycin (Invitrogen) as previously described *(9)*. Briefly, after 2 hours of 10ug/ml of mytomicin C (Sigma) treatment and washing with PBS, MS5 and OP9 cells were seeded at a 1:6 ratio in 96- or 24-well plates 24 hours before culturing hematopoietic cells. For 96-well plates, 3.75 × 10^4^ MS5 cells and 6.25 × 10^3^ OP9 cells were seeded per well, and for 24-well plates, 1.5 × 10^5^MS5 and 2.5 × 10^4^ OP9 cells were seeded per well. Purified progenitor populations were cultured in medium containing 100ng/ml Flt3L (Celldex), 20ng/ml SCF (Peprotech) and/or 10ng/ml GM-CSF (Peprotech), with half media change every 7 days. Cells were harvested between days 3-21 for flow cytometry analysis. For JD culture, we used the condition developed by Notta et al. ^22^, In brief, MS5 was plated in flat-bottom 96-well plate at the density of 5,000 cells Myelocult medium (H5100, Stem cell technologies) per well and given 24-48 hours to attach. Before cell sorting, Myelocult media was carefully removed and 200uL media was added. We used serum free media (StemPro34 SFM with nutrient, Life Technologies) supplemented with SCF_100ng/mL, FLT3_20ng/mL, TPO_100ng/mL, EPO *3units/mL, IL-6* 50ng/mL, IL-3_ 10ng/mL, IL-11_ 50ng/mL, GM-CSF_ 20ng/mL, LDL_ 4ug/mL, 2-ME, L-Glutamine, Pen-strep. At week 2, half medium was changed. Colony forming unit assay was performed using MethoCult™ (Stemcell, H4434), containing SCF, GM-CSF, IL-3 and EPO. Colony-forming unit–cells (CFU-C) were counted after 14 days of culture.

For Flt3L culture of mouse progenitor cells, 200 purified LMPPs from CD45.2 WT, irf8^+/-^ or Irf8^-/-^ mice were seeded with 3×10^5^ CD45.1 total bone marrow cells in 200 ul of RPMI culture with 10% FCS, L-glutamin, 1mM L-glutamine, 1mM Sodium Pyruvate, 10mM HEPES, NEAA ug/ml Flt3L in 96 well round bottom plates, and cultured for 2- 7 days prior to analysis.

To determine cellular divisions in culture, input populations were labeled for 15 min with 5μM carboxyfluorescein diacetate succinimidyl ester (CFSE, Molecular Probes) or cell track violet (CTV, Molecular Probes) at 37°C and washed with complete alpha MEM prior to culture or *in vivo* transfer.

### Tracing of single cell progeny

For daughter cell tracing, HSC/MPPs and GMDPs were first sorted as a population based on their surface marker phenotype described in Table 1. Washed cells in cold PBS were incubated in 500ul of alpha MEM medium (Invitrogen) without serum containing Vybrant® DiD cell-labeling solution (1:200 dilution, Life Technologies) for 20 min at 37°C water bath. Cells were spun down at 1500 rpm for 5 min and washed twice with complete alpha MEM medium (Invitrogen) with 10% FCS and penicillin/streptomycin (Invitrogen). Cells were then resuspended in PBS and resorted as DiD^+^ directly into MP+FSG 96-well plates at a 1cell/well concentration. Each cell was monitored daily for division using either an EVOS FL Cell imaging system (Life Technologies) or an Inverted Leica fluorescent microscope DM16000 (Leica) equipped with a Cy5 light source. This method allowed us to trace up to more than 5 divisions (>50 daughter cells) from a single initial cell (data not shown).

When the initial cell generated 4 granddaughter cells, as detected by microscopy, we collected and manually aliquoted them into 8 separate wells of a MP+FSG 96-well plate (0.5 cells/well) in order to increase the probability of seeding 1 granddaughter cell into secondary wells. GMDP-derived granddaughter cells were cultured for 2 weeks and HSC/MPP-derived granddaughter cells were cultured for 3 weeks before harvest. Ancestors that only had one viable granddaughter cell by the end of the culture were not included for analysis.

The ancestor's potency was inferred by the sum of the all granddaughters. The lineage bias was determined by lineage that exhibited highest yield. The progeny exhibited the same lineage bias with its ancestor was considered bias-inherited, and the progeny exhibited different lineage bias from its ancestor was considered bias-switched.

### *In vivo* transplantation into NSG mice

NSG mice were injected intraperitoneally with busulfan (Sigma, 30ug/g of body weight) to ablate endogenous hematopoietic system 2 days prior to human CD34^+^ cell transfer. Human progenitors purified from cord blood were resuspended in 10μl PBS and intratibially injected with a Hamilton syringe and 27-gauge needle. 7 or 14 days after transplantation, bone marrow was harvested from recipient mice and analyzed for human CD45^+^ cells. NSG mice were used to characterize progenitor hierarchy and for *in vivo* CFSE-labeled HSC/MPP transfer experiments. NSG-SGM3 mice were used to determine *in vivo* progenitor lineage potential.

### Clonal analysis of progenitors

Progenitors were individually sorted as single cells directly into 96-well plates containing mitomycin C-treated stromal cells. Immediately after, media containing cytokine mix was added. Each well was harvested after 7-21 days of culture and stained with LIVE/DEAD®, CD45, CD66b, CLEC9a, CD14, CD1c, CD303, CD141, CD19 and CD56. Positive clones were determined by the detection of at least 2 (for CDPs) or 7 (for all other progenitors) events in any of the lineage specific gates.

### Heat map, PCA and MDS

Clonal output data was normalized with the procedure described in DESeq, assuming the geometric mean of total clonal output for a single progenitor phenotype across different donors should be similar. Normalized cell counts were scaled by log base 10, clustered by unsupervised hierarchical clustering function with *hclust {stats}* (R Statistical Software) and visualized with *heatmap.2 {gplots}*. Complete linkage method was used for clustering, with the distance metric between progenitors defined by Euclidean distance. The ordering of leaves was optimized with the *cba* package, so that the sum of similarities between adjacent leaves can be maximized while keeping the hierarchical tree structure unchanged. Principal component analysis was performed with the function *prcomp()* in R, with centering , scaling and cor options on. Ancestral similarity between each pair of cell lineages was calculated as Spearman's rho with *cor {stats}*, using their yield from 2,247 progenitors as 6 dimensions. Distance (d = 1 – rho) between each cell type was calculated. The distance matrix was reduced to 2 dimensions with MDS via *cmdscale {stats}*, with eig = True and k =2. Potency similarity between each pair of progenitors was calculated in a similar way differing by transposing the counts matrix first.

### Visualization of development trajectories using t-SNE map

To identify putative developmental trajectories from HSCs to six individual blood lineages through clonal output, we used t-Distributed Stochastic Neighbor Embedding (t-SNE) technique to do dimension reduction for visualization. First we further normalized the yield of each lineage with DESeq to make sure the geometric mean of each progeny type yield is similar across all progenitors (the culturing system is producing less pDCs than other types of progenies). Then we take the normalized clonal output as input to the Barnes-Hut t-SNE package ^38^ with parameters perplexity = 20 and theta = 0.3 for visualization (cord blood samples). t-SNE minimized the Kullback-Leibler divergence between two similarity distributions, with one measures pairwise similarities of the input objects and the other measures pairwise similarities of the projected low-dimensional points in the embedding space. In our case, the similarities in the high dimension space between pairwise progenitor cells is calculated using the joint probabilities with an isotropic Gaussian kernel over the number of their terminal outputs by symmetrizing two conditional probabilities:

$$P_{j|i}=\frac{\exp\left(\frac{-{\left(x_{i}-x_{j}\right)}^{2}}{2{\sigma_{i}}^{2}}\right)}{\sum_{k\neq i}\exp\left(\frac{-{\left(x_{i}-x_{k}\right)}^{2}}{2{\sigma_{i}}^{2}}\right)},P_{i|i}=0$$

$$P\left(i,j\right)=\frac{P_{j|i}+P_{i|j}}{2N}$$

where *x_i_* and *x_j_* are the logarithm of terminal cells number vectors for progenitors *i* and *j*, *σ_i_*, the bandwidth of the Gaussian kernel, is determined in a way that the perplexity of the conditional distribution *P_i_* equals a user defined perplexity parameter. *P*(*i, j*) has a range of 0 to 1, and can be interpreted as the transition probability of a diffusion process from progenitor *i* to progenitor *j* (*i* ≠ *j*), as cells are moving towards more differentiated state in a heterogeneous and stochastic way similar to diffusion dynamics. Then we use *P*(*i, j*) as input similarities to t-SNE for visualization in 2-dimension space and generate the diffusion map.

### Distance computation of progenitors to track and assignment of cell-type specific lineage bias

To determine the distance of each cell to every lineage in the diffusion map, we first established a backbone of each lineage using cells with 70% commitment degree to that lineage. Commitment degree is defined as the ratio of one lineage yield over the sum of all six lineages yield, ranging from 0 to 1. 0 means no potential and 1 means fully committed. We then computed the Euclidean distance between every pair of cells. The distance of a cell to a track is defined as the closest distance to any of the cells on the backbone for all tracks. We finally assigned as the closest track, the track to which the cell is closest.

### Calculation of correlation between TF dosage and lineage potency

For correlation between IRF8/PU.1 dosage and lineage potency of all progenitors, we first calculated the percentage of subpopulations identified by relative IRF8/PU.1 dosage and lineage bias composition of each progenitor, then calculated Pearson correlation coefficiencies between them for all progenitors. Student's t-test for transformed correlation ^39^ were used to access the statistical significance of correlation.

### Statistical analysis

Statistical tests are described in their corresponding figure legends. All values indicated are means + standard error of the mean, or + standard error of proportion, unless otherwise specified. For result comparison, we have used one-way analysis of variance (ANOVA), unpaired or paired two-tailed Student's t-test, Spearman's correlation test, Fisher's exact test and Pearson correlation test. Statistical analysis was done with GraphPad prism v7.0, Microsoft Excel, R or R Studio. Significance was set at p < 0.05. Data exclusion criteria was only applied to determine unproductive clones.

### Data availability statement

Clonal data can be found in the Supplementary Information. All other data that support the findings of this study are available from the corresponding authors upon request.

## Supplementary Material

1

2

## Figures and Tables

**Table 1:** Characterization of progenitor populations in human cord blood

Population	Phenotype	% of CD34+		Lineage output							Ref.
				CFU			MS5				
		CB	BM	ME	G	M	G	M	DC	L	
Hematopoietic stem cell (HSC)	CD34+CD38-CD45RA-CD90+	3.79	1.70	+	+	+	+	+	+	+	*19*
Multipotent progenitor (MPP)	CD34+CD38-CD45RA-CD90-	19.0	11.3	+	+	+	+	+	+	+	*19*
Lymphoid-primed multipotent progenitor (LMPP)	CD34+CD38-CD45RA+CD10-	1.36	16.5		+	+	+	+	+	+	*20*
Multilymphoid progenitor (MLP)	CD34+CD38-CD45RA+CD10+CD7+/-	4.88	0.80			+		+	+	+	*19*
B and NK cell progenitor (BNKP)	CD34+CD38+CD45RA+CD123int/-CD115-CD10+	3.12	2.20							+	*19*
Common myeloid progenitor (CMP)	CD34+CD38+CD45RA-CD10-CD123int	40.0	33.8	+	+	+	+	+	+		*18*
Granulocyte/Monocyte/DC progenitor (GMDP)	CD34+CD38+CD45RA+CD10-CD123int	11.4	11.2		&plus;	&plus;	&plus;	&plus;	&plus;		*7*
Monocyte/DC progenitor (MDP)	CD34+CD38+CD45RA+CD123intCD115+	0.81	5.60			+		+	+		*7*
Common DC progenitor (CDP)	CD34+CD38+CD45RA+CD123hiCD115-	0.54	3.00						+		*7*
Megaerythrokaryocyte progenitor (MEP)	CD34+CD38+CD45RA-CD10-CD123-	15.3	11.4	+							*18*

CFU, Colony forming unit assay; MS5, MS5 stromal cell culture assay

ME, erythrocyte and megakaryocyte; G, granulocyte; M, monocyte; DC, dendritic cell; L, B/NK lymphocytes.
