# Supplementary material for: Lineage specification of human dendritic cell is marked by expression of the transcriptionl factor IRF8 in HSCs and MPPs
Source: Nat Immunol. Author manuscript; Available in PMC 2017 Dec 27. (PMC5743223; doi:10.1038/ni.3789)
Supplement: 1 [file NIHMS881985-supplement-1.doc]

| 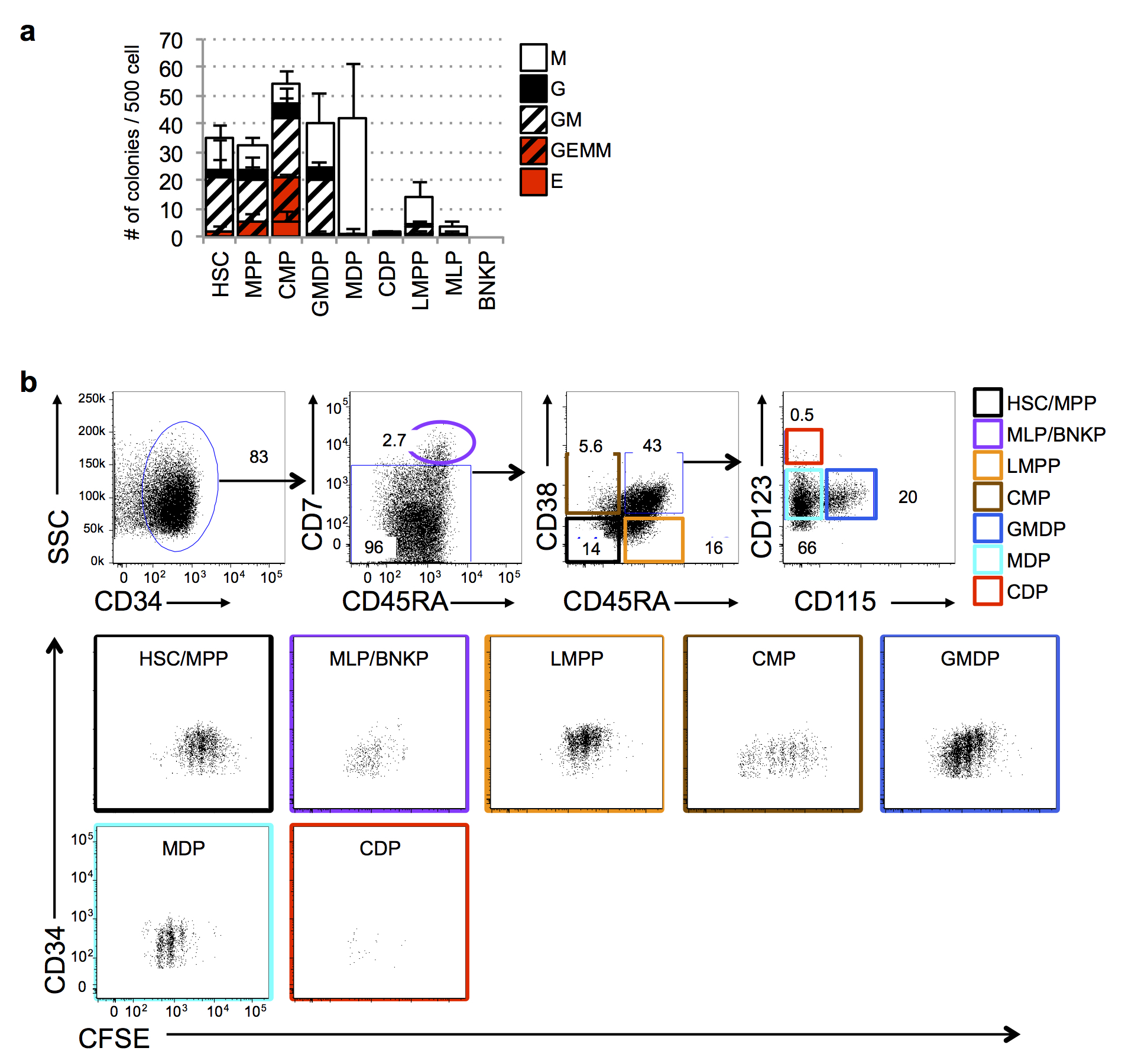  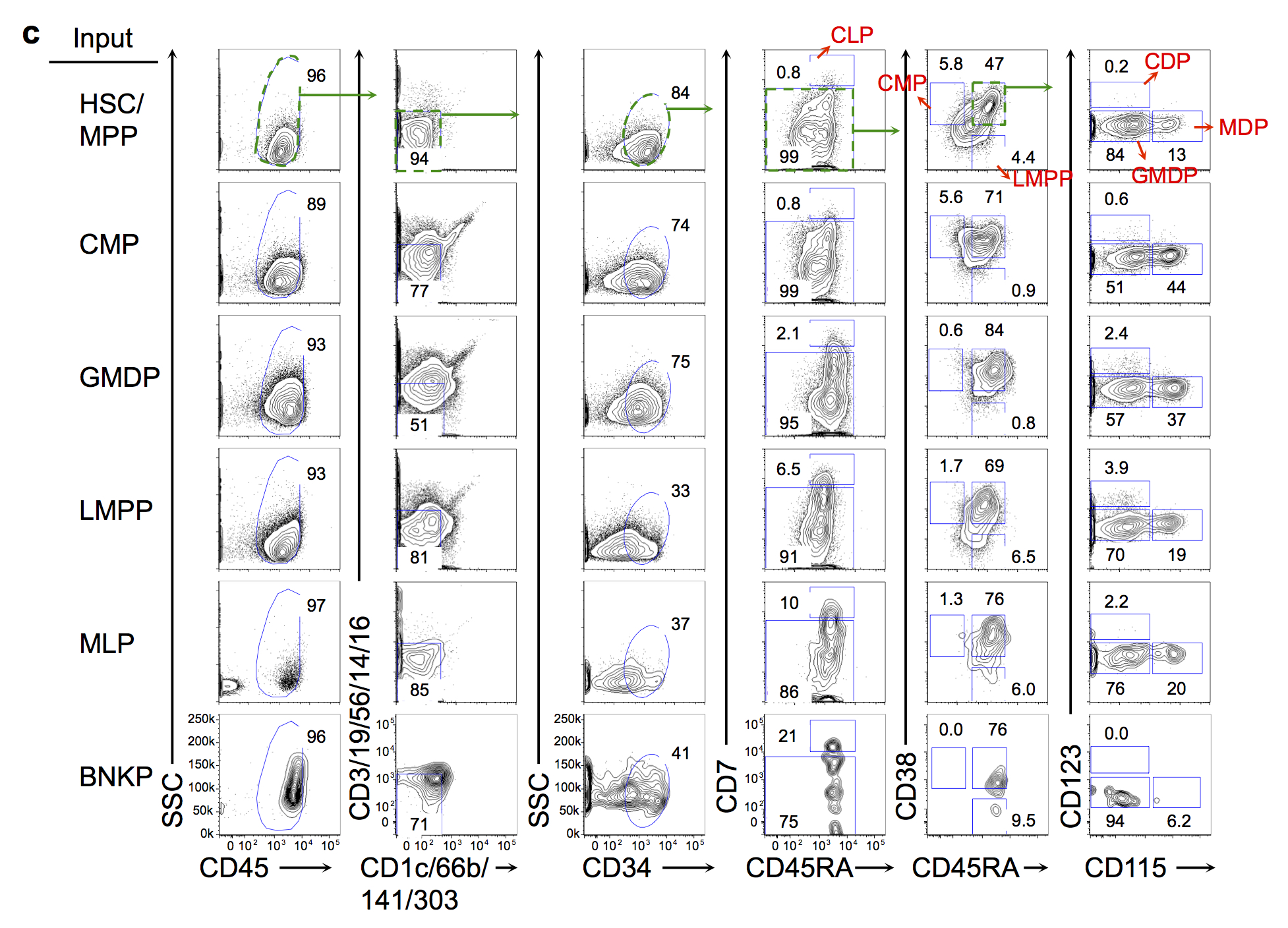  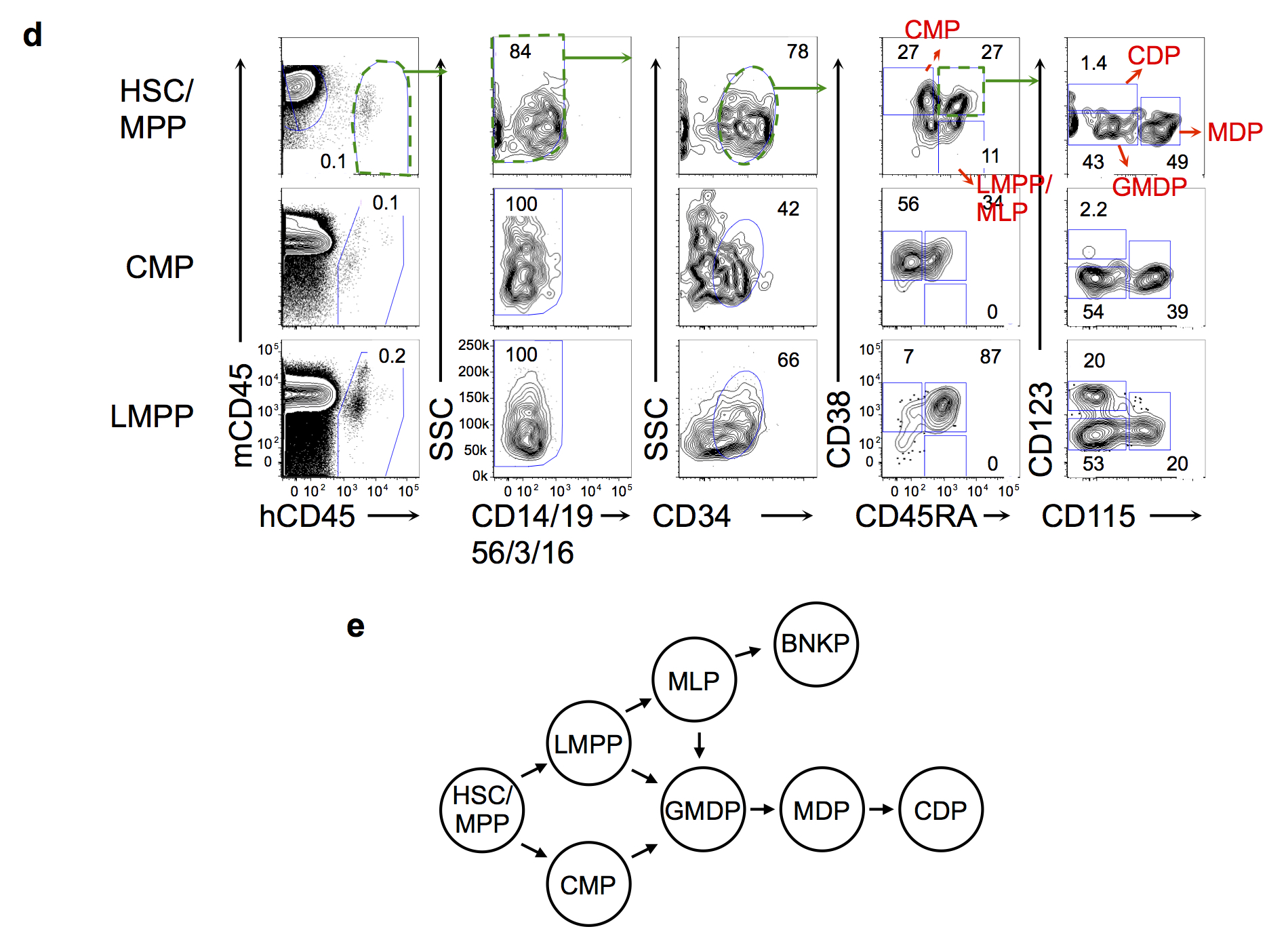 |
| --- |
| **Supplementary Figure 1** |
| Colony-Forming Unit (CFU) and MP+FSG culture reveal developmental convergence for different marker-pure progenitor cells isolated from cord blood. |
| (**a**) Stacked columns showing colony-forming units (CFU) produced by indicated populations after culturing in methylcellulose for 14 days. M, macrophage; G, granulocyte; GM, granulocyte and macrophage; GEMM, granulocyte, erythrocyte, megakaryocyte and macrophage; E, erythrocyte. Bars, mean averages; error bars, SEM. (**b**) Flow cytometry plots (top) showing the gating strategy to identify downstream progenitors from HSC/MPPs after culturing in MP+FSG for 6 days. Colored frames (bottom) showing the populations that have been concatenated in order to plot Fig. 1c and their respective degree of cell division as indicated by CFSE. (**c, d**) Representative flow cytometry plots showing all downstream populations produced by the input progenitor after (**c**) in vitro culture or (**d**) in vivo transfer. Plots in **c** are the original gating strategy for **Fig. 1d**. Numbers indicate percentages from parental gate. (**e**) Schematic picture summarizing the developmental relationship of CD34+ progenitors. Data shown are representative of four (**a**), four (**b-c**), and three (**d**) independent experiments. |
| 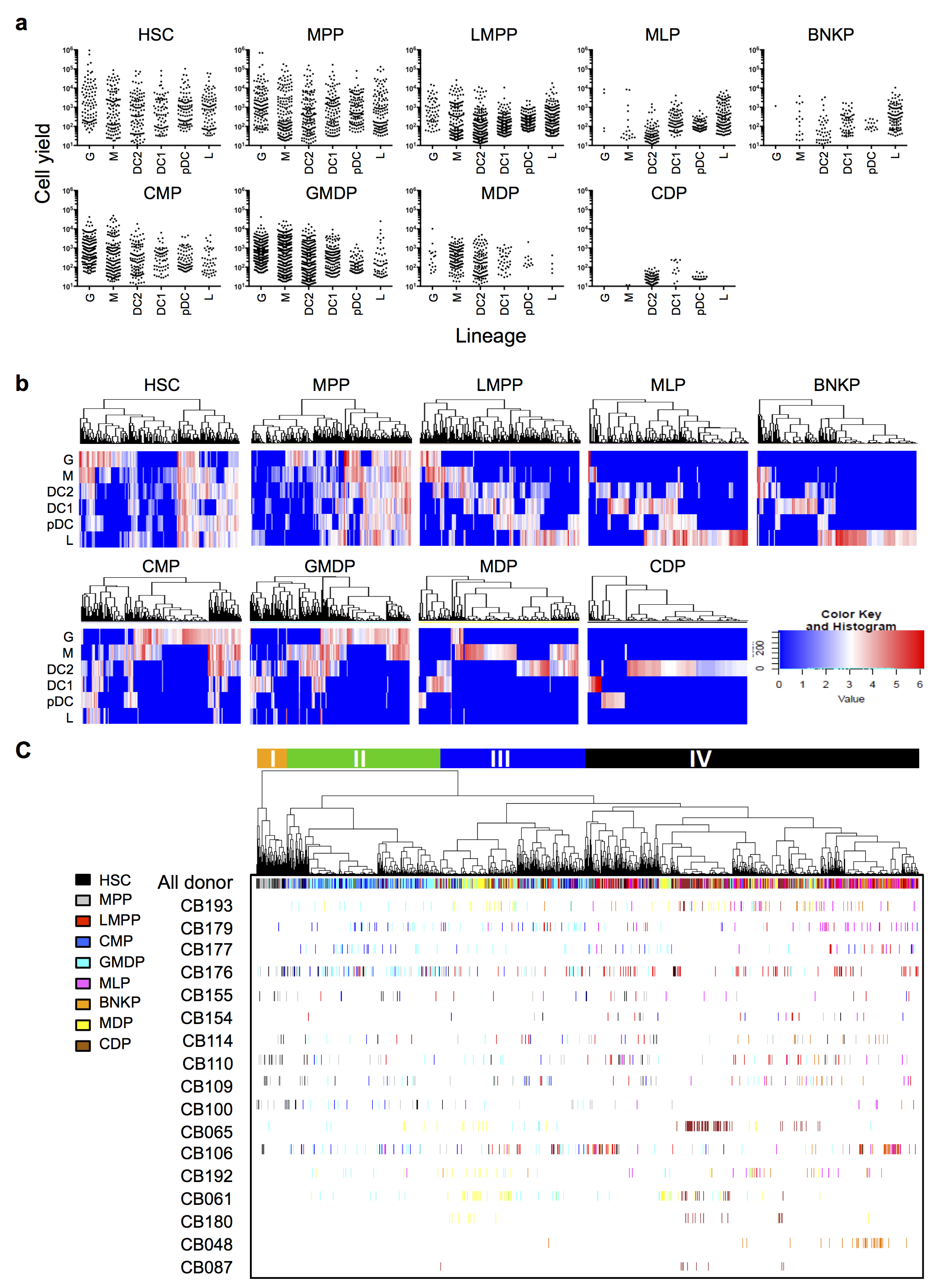 |
| **Supplementary Figure 2** |
| Clonal output of single cord blood progenitors reveals lineage heterogeneity within marker-pure progenitor populations. |
| (**a**) Scatter plots showing the number of cells produced for each lineage from all clones of each indicated progenitor type. (**b**) Heat maps showing normalized output of mature cells of each type (row) from each single cell (column) in indicated progenitor populations. (**c**) Plots showing distribution of single progenitor cells (columns) from individual donors (rows) within the four clusters (top) identified by unsupervised hierarchical clustering (Euclidean distance and complete linkage) based on the clonal outcome, as shown in **Fig. 2g**. Data represent cumulative clones from seventeen cord blood donors (**a-c**). |
| 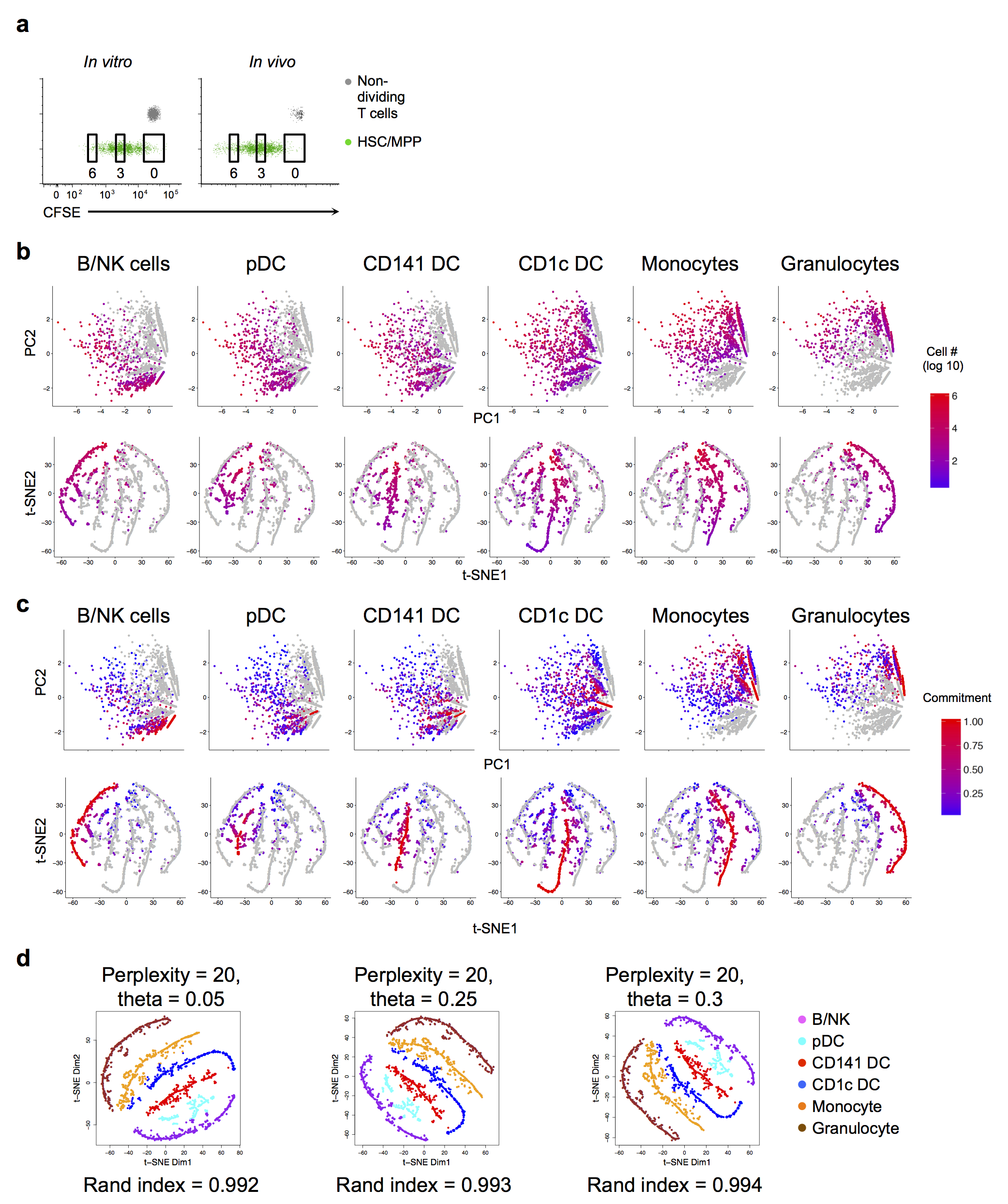 |
| **Supplementary Figure 3** |
| t-SNE analysis and visualization of quantitative clonal data resembles PCA results. |
| (**a**) Flow cytometry plots showing HSC-derived cells sorted from division 0, 3 and 6 (boxes) for clonal analysis in either MP+FSG culture (left) or in NSG mice (right) as described in Fig. 3a. (b, c) PCA analysis (upper panels) or t-SNE analysis (lower panels) visualizing clonal output data from 2,247 individual progenitor cells, with color labeling according to (**b**) the number of cells produced or (**c**) the degree of commitment toward the stated cell lineage (top labels). (**d**) t-SNE plots showing consistent clusters and patterns from total clonal data despite different thresholds for perplexity, theta and random index parameters from three runs. Data represent cumulative clones from seventeen cord blood donors (**a-d**). |
| 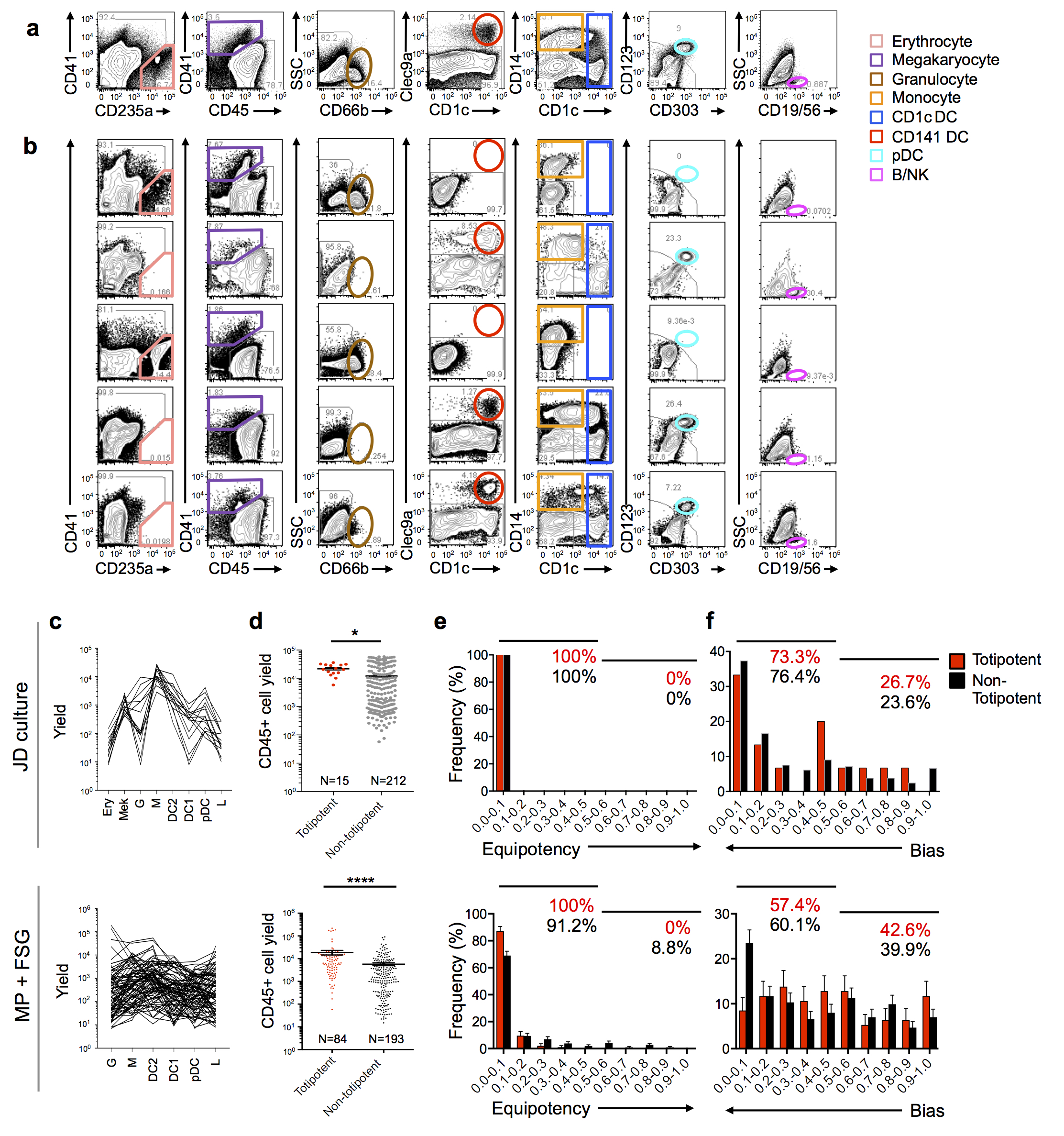 |
| **Supplementary Figure 4** |
| HSC/MPPs show lineage bias in a permissive culture system that supports eight lineages. |
| (**a-b**) Flow cytometry plot showing lineage output of representative HSC/MPP clones in a culture system described by John Dick’s group (Notta et al. Science 2016), referred to here as JD culture. Data represent concatenated (**a**) and individual (**b**) clones. The following eight lineages are highlighted in gates with different colors: erythrocyte (Er), megakaryocyte (Mk), granulocyte (G), monocyte (M), CD1c cDCs (DC2), CD141 cDCs (DC1), and B/NK cells (L). (**c**) Line plot showing totipotent HSC/MPP clones in JD (top) and MP+FSG culture (bottom). Clones were plotted according to the yield of each lineage, where each line is an individual clone. (**d**) Scatter plot comparing the yield of all progenies from totipotent or non-totipotent HSC/MPP clones in JD culture (top) and MP+FSG culture (bottom). (**e-f**) Frequency distribution of all non-unipotent clones, based on their degree of equipotency (**e**) and bias (**f**) from JD culture (top) and MP+FSG culture (bottom). Numbers indicate the cumulative % of clones for which the ratio is <0.5 (left line) or >0.5 (right line). Red bars indicate totipotent HSC/MPP clones; black bars indicate non-totipotent HSC/MPP clones. Data shown are representative of three independent experiments (for JD culture), or cumulative clones from seventeen cord blood donors (for MP+FSG culture). * p <0.05; **** p <0.0001 (unpaired two-tailed Student’s t-test). |
| 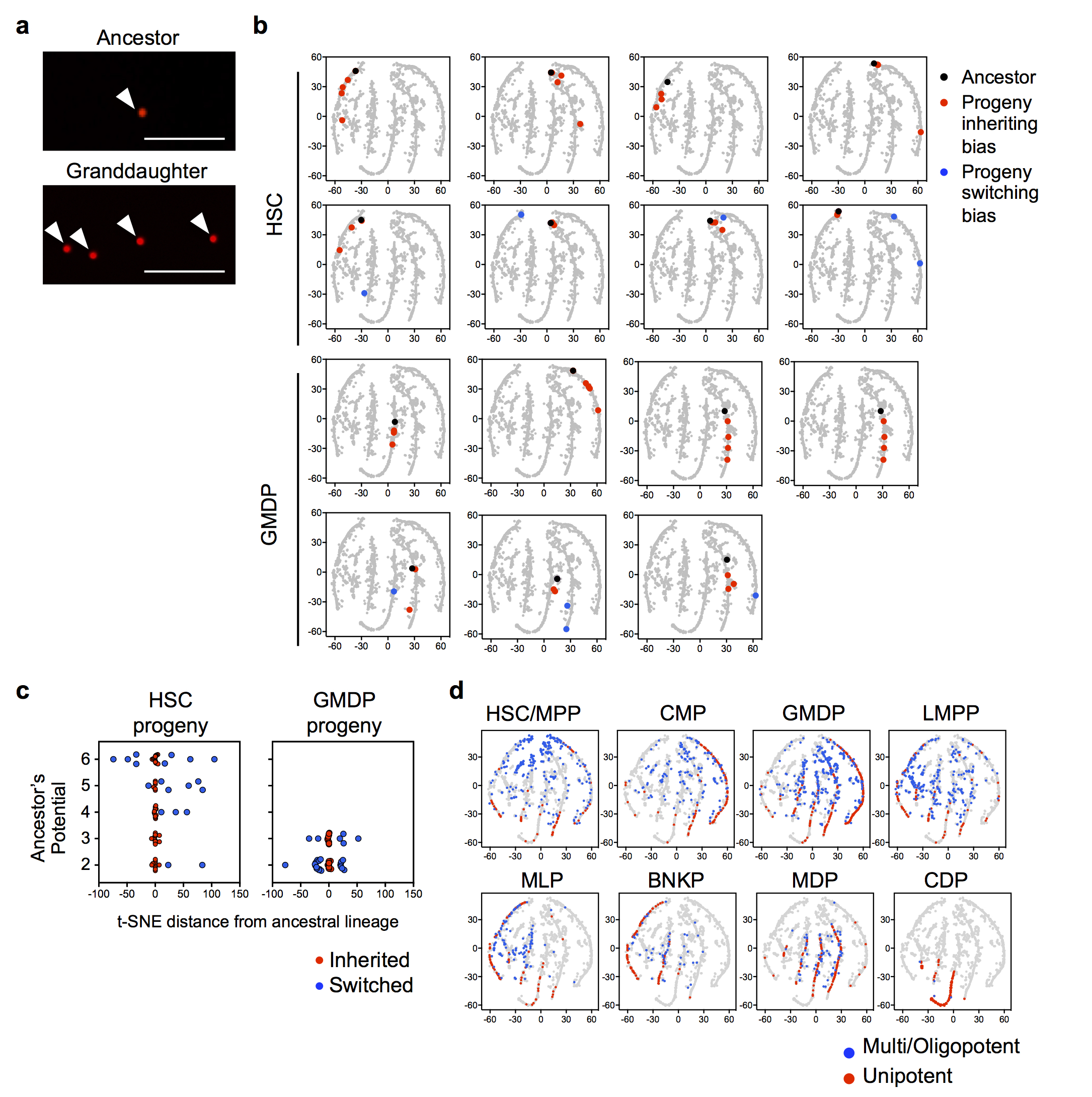 |
| **Supplementary Figure 5** |
| Lineage switch by HSC/MPP-derived granddaughter cells are more flexible than those from GMDPs. |
| (**a**) Fluorescence microscopy images showing DiD-labeled single ancestor cell and four granddaughter cells after 2–4 days of culture in MP+FSG condition. Scale bar: 100μm (**b**) Each t-SNE plot showing the developmental position of the ancestral clone (black) and progeny that have either inherited (red) or switched (blue) lineage bias. (**c**) Dot plots summarizing the distance of all progeny of HSCs and GMDPs, including bias-inherited (red) and bias-switched (blue) progeny, to its ancestral track. (**d**) t-SNE maps showing distribution of clones from each marker-pure population. Color indicates multipotency or unipotency. Data shown are representative of three independent experiments (**a-c**), or cumulative clones from seventeen cord blood donors (**d**). |
| 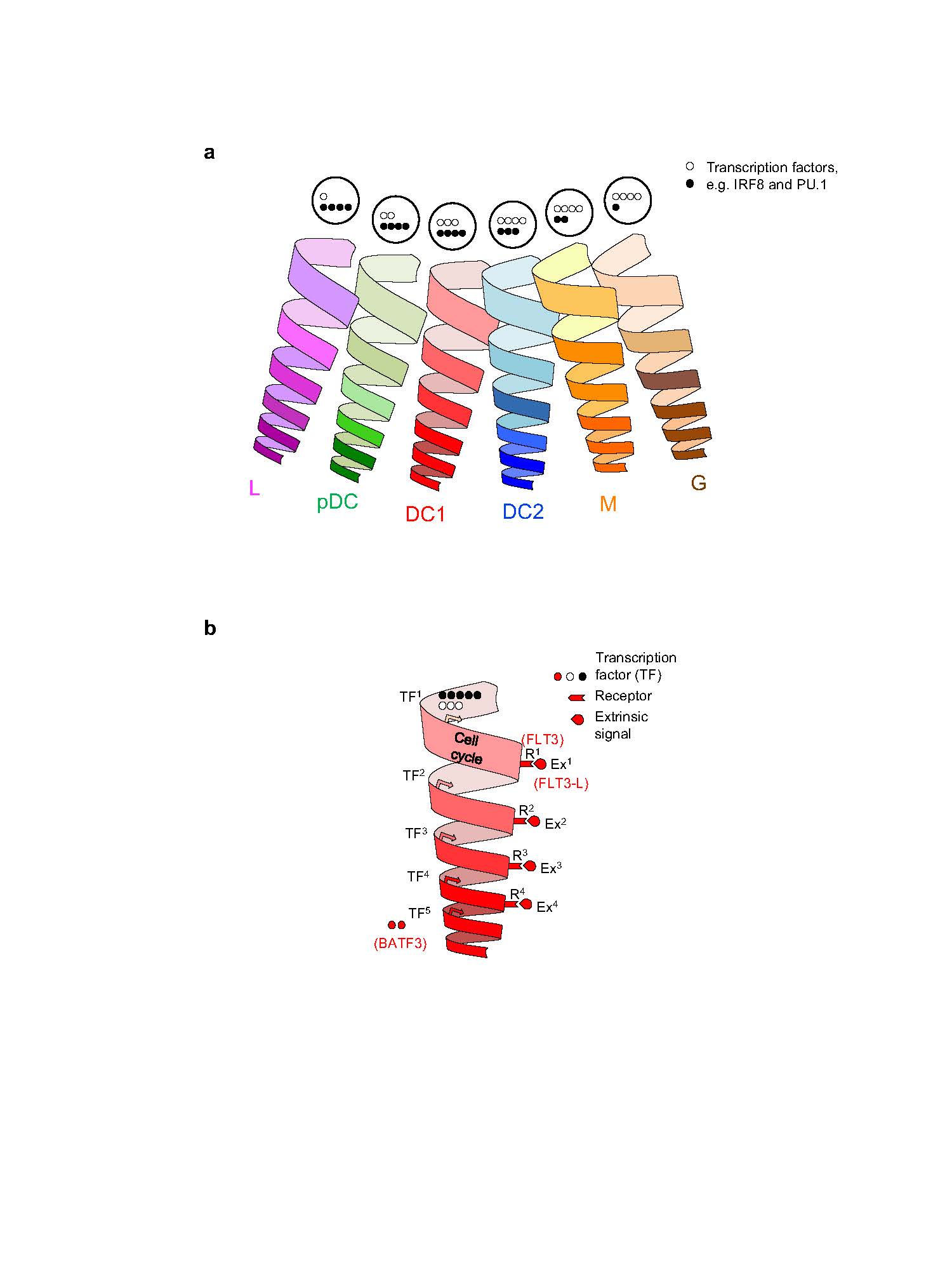 |
| **Supplementary Figure 6** |
| Graphical representation of the heritable lineage bias model. |
| (**a**) A limited transcription factor set and environmental signals (Ex) are potentially sufficient to initiate L (B/NK), pDC, DC1 (CD141+ DC), DC2 (CD1c+ DC), M (Monocyte) and G (Granulocyte) lineages that can be memorized and reinforced over division through dose-dependent function and the process of recursive interaction described in **b**. (**b**) Graphic representation of feedback cycle of CD141 DC lineage commitment. Transcriptional programs established in HSCs by different dosage combinations of common transcription factors (TFs) including PU.1 and IRF8 (TF^1^) cause initial expression of cell surface receptors (R^1^), which can in turn bind extrinsic signals (Ex^1^). These signals drive cell division but also orchestrate intracellular signals that give rise to a modified intrinsic transcriptional program (TF^2^) and expression of new receptors (R^2^) that can integrate additional extrinsic signals (Ex^2^). The recursive interaction between intrinsic and extrinsic signals over each cycle enables progenitors to “memorize” the preferential lineage identity conferred by the initial transcriptional program at the HSC stage, and to strengthen that lineage identity through expression of TFs like BATF3, which is required for terminal commitment. |
