# Supplementary material for: Lineage specification of human dendritic cell is marked by expression of the transcriptionl factor IRF8 in HSCs and MPPs
Source: Nat Immunol. Author manuscript; Available in PMC 2017 Dec 27. (PMC5743223; doi:10.1038/ni.3789)
Supplement: 2 [file NIHMS881985-supplement-2.pdf]

MP+FSG culture

Source	Progenitor	Batch	Clone.name	CD45	Granulocytes	Monocytes	CD1c DCs	CD141 DCs	pDCs	B/NK cells
CB	MDP	CB193	_001	1535	88	116	254	43	269	91
CB	LMPP	CB179	_002	959	123	39	81	371	107	25
CB	CMP	CB179	_003	609	31	19	148	133	120	29
CB	GMDP	CB177	_004	1008	7	17	10	10	15	895
CB	GMDP	CB177	_005	291	16	34	99	18	72	12
CB	CMP	CB177	_006	815	14	92	37	19	141	156
CB	MPP	CB176	_007	7308	362	547	1806	760	917	2507
CB	MPP	CB176	_008	11147	30	1924	735	12	428	111
CB	MPP	CB176	_009	19285	2027	9135	1598	29	30	19
CB	MPP	CB176	_010	3073	266	167	183	41	589	500
CB	MPP	CB176	_011	21007	455	12575	2321	500	21	12
CB	MPP	CB176	_012	13338	245	6158	5104	1035	192	146
CB	LMPP	CB176	_013	691	91	120	14	24	23	127
CB	LMPP	CB176	_014	929	45	154	176	16	98	136
CB	HSC	CB176	_015	6370	16	190	1741	40	1708	147
CB	HSC	CB176	_016	18209	721	11235	3361	114	124	27
CB	HSC	CB176	_017	5493	31	253	1118	180	1358	720
CB	HSC	CB176	_018	4095	207	957	20	14	150	774
CB	HSC	CB176	_019	6195	33	573	1441	209	697	439
CB	HSC	CB176	_020	9649	30	2167	2044	37	714	41
CB	HSC	CB176	_021	2496	48	466	963	302	426	173
CB	GMDP	CB176	_022	32960	4787	3031	42	175	29	228
CB	CMP	CB176	_023	4020	370	2288	991	153	142	36
CB	CMP	CB176	_024	2009	20	273	862	724	52	16
CB	CMP	CB176	_025	3018	20	1997	578	139	28	7
CB	MPP	CB155	_026	3828	281	26	692	225	473	202
CB	MPP	CB155	_027	5701	854	1487	2575	580	34	38
CB	MPP	CB155	_028	8518	3079	782	887	71	163	663
CB	MPP	CB155	_029	16520	109	358	7489	4363	1330	643
CB	MPP	CB155	_030	1704	34	80	1191	231	84	48
CB	MPP	CB155	_031	5847	454	1875	727	361	261	1592
CB	HSC	CB155	_032	11653	1005	8771	793	25	189	21
CB	HSC	CB155	_033	2528	1196	77	360	588	96	172
CB	HSC	CB155	_034	8388	1244	5045	90	108	140	834
CB	HSC	CB155	_035	6310	3551	142	419	136	44	1667
CB	HSC	CB155	_036	4131	991	120	1499	1388	47	83
CB	MPP	CB154	_037	742	68	80	57	78	70	236
CB	HSC	CB154	_038	3229	65	861	1085	830	55	27
CB	MPP	CB114	_039	1535	150	88	251	885	7	44
CB	MPP	CB114	_040	6494	157	39	62	25	21	367
CB	LMPP	CB114	_041	956	9	545	224	37	19	36
CB	LMPP	CB114	_042	1318	126	124	150	40	74	164
CB	HSC	CB114	_043	2507	24	177	631	67	63	52
CB	HSC	CB114	_044	4739	890	719	206	682	32	720
CB	HSC	CB114	_045	2309	51	54	833	160	194	188
CB	MPP	CB110	_046	14899	46	2137	1983	11	462	28
CB	MPP	CB110	_047	5920	8	62	2205	1398	304	146
CB	MPP	CB110	_048	6093	1011	4144	419	404	22	47

CB	MPP	CB110	_049	511	7	21	92	345	30	8
CB	MPP	CB110	_050	684	26	26	120	301	44	105
CB	MPP	CB110	_051	1323	59	29	333	230	309	64
CB	MPP	CB110	_052	5582	52	174	72	690	235	4170
CB	MPP	CB110	_053	14050	4363	7861	680	62	17	26
CB	MPP	CB110	_054	3108	20	291	1459	177	409	27
CB	MPP	CB110	_055	311	13	63	21	186	8	15
CB	MPP	CB110	_056	14274	993	1125	4240	4357	1467	1658
CB	MPP	CB110	_057	13352	1042	117	785	30	173	1152
CB	MPP	CB110	_058	20633	583	426	2613	812	762	9790
CB	MPP	CB110	_059	4279	100	33	42	128	40	3128
CB	MPP	CB110	_060	14214	382	598	1985	8384	563	1329
CB	MPP	CB110	_061	18560	77	2164	9179	1861	777	114
CB	MPP	CB110	_062	11976	54	453	4152	3194	1446	1419
CB	MPP	CB110	_063	5849	20	70	394	4710	463	167
CB	MPP	CB110	_064	7144	1472	362	138	586	441	3944
CB	MPP	CB110	_065	12233	707	194	1195	2920	597	5019
CB	HSC	CB110	_066	14574	151	1175	1348	98	1179	1367
CB	HSC	CB110	_067	5444	16	548	1959	532	397	201
CB	HSC	CB110	_068	5353	251	756	712	3165	125	15
CB	HSC	CB110	_069	4146	169	402	134	269	340	2301
CB	HSC	CB110	_070	3680	660	973	56	16	23	13
CB	HSC	CB110	_071	15756	1249	1226	799	366	2314	5097
CB	MPP	CB109	_072	11960	125	379	5232	5105	305	146
CB	MPP	CB109	_073	5237	41	266	1839	613	592	13
CB	MPP	CB109	_074	1319	20	177	294	566	97	88
CB	MPP	CB109	_075	4932	22	157	342	595	1428	1220
CB	MPP	CB109	_076	20744	74	1015	9322	9196	913	26
CB	MPP	CB109	_077	8222	591	56	142	730	908	5179
CB	MPP	CB109	_078	9478	45	62	272	357	633	477
CB	MPP	CB109	_079	1138	13	73	146	140	282	199
CB	HSC	CB109	_080	15154	12	1449	5921	1917	1165	29
CB	HSC	CB109	_081	13383	14	1778	3334	5620	1858	219
CB	HSC	CB109	_082	13652	714	499	1728	4800	1624	1880
CB	HSC	CB109	_083	12151	2281	5722	965	262	88	9
CB	HSC	CB109	_084	2198	8	16	539	670	448	43
CB	MPP	CB100	_085	865	13	57	282	188	21	302
CB	MPP	CB100	_086	173126	46289	32324	42226	4722	5213	14302
CB	MPP	CB100	_087	4737	1002	1622	1523	252	194	66
CB	MPP	CB100	_088	175830	137758	4367	3564	291	138	24231
CB	MPP	CB100	_089	10400	1655	7150	478	126	84	529
CB	MPP	CB100	_090	26587	23144	2503	46	18	134	148
CB	MPP	CB100	_091	72781	2815	8080	23299	133	2304	2615
CB	HSC	CB100	_092	614	28	54	79	28	21	303
CB	HSC	CB100	_093	1776	494	350	20	114	14	764
CB	HSC	CB100	_094	161908	287	3092	26311	9	2612	1258
CB	HSC	CB100	_095	14758	2137	2731	5396	3286	398	607
CB	HSC	CB100	_096	1615	280	57	11	50	12	1128
CB	HSC	CB100	_097	1387	76	572	324	13	104	291
CB	HSC	CB100	_098	186503	6335	38315	54159	2914	4085	4026

CB	HSC	CB100	_099	217713	189633	10033	487	9	266	13459
CB	HSC	CB100	_100	55155	4283	7711	17797	56	3044	7998
CB	HSC	CB100	_101	1908	14	62	74	71	15	316
CB	CMP	CB100	_102	4573	98	1704	2386	39	102	26
CB	CMP	CB100	_103	4189	100	1981	1482	140	34	171
CB	GMDP	CB065	_104	13414	26	3165	1054	803	68	1329
CB	GMDP	CB065	_105	10218	85	1668	273	160	7	4359
CB	GMDP	CB193	_106	491	178	17	130	119	5	38
CB	GMDP	CB193	_107	2874	519	1216	557	79	4	7
CB	BNKP	CB193	_108	6566	7	743	1878	0	23	18
CB	MLP	CB179	_109	968	7	7	422	358	95	3
CB	LMPP	CB179	_110	306	1	13	25	53	34	166
CB	LMPP	CB179	_111	435	0	21	119	258	12	12
CB	LMPP	CB179	_112	121	0	8	9	23	26	42
CB	GMDP	CB179	_113	212	17	58	28	94	12	1
CB	GMDP	CB179	_114	901	4	264	162	116	47	258
CB	CMP	CB179	_115	1296	24	19	363	793	3	10
CB	LMPP	CB177	_116	120	0	17	21	26	13	26
CB	GMDP	CB177	_117	113	12	4	30	20	20	25
CB	GMDP	CB177	_118	953	1	9	94	48	87	625
CB	CMP	CB177	_119	1116	129	285	264	7	8	3
CB	CMP	CB177	_120	2279	95	168	1370	173	76	1
CB	CMP	CB177	_121	4856	35	36	1887	2	84	409
CB	CMP	CB177	_122	740	1	85	505	88	27	20
CB	CMP	CB177	_123	2320	0	7	460	25	405	12
CB	CMP	CB177	_124	2106	2	8	825	25	12	119
CB	MPP	CB176	_125	8173	1171	5225	1559	54	55	0
CB	MPP	CB176	_126	6877	9	4036	346	23	2	13
CB	MPP	CB176	_127	15091	35	354	33	0	15	8
CB	MPP	CB176	_128	30194	1250	4315	190	0	9	10
CB	MPP	CB176	_129	3956	120	13	63	1	165	127
CB	MPP	CB176	_130	1511	0	146	242	15	31	12
CB	LMPP	CB176	_131	455	40	130	160	73	45	6
CB	LMPP	CB176	_132	221	117	0	13	53	28	10
CB	LMPP	CB176	_133	450	0	22	10	10	71	75
CB	HSC	CB176	_134	5668	178	4450	926	76	28	0
CB	HSC	CB176	_135	14908	538	9238	1761	16	42	2
CB	HSC	CB176	_136	4216	76	706	738	13	44	3
CB	HSC	CB176	_137	4184	2472	877	719	92	15	3
CB	HSC	CB176	_138	3934	7	1032	1111	57	26	0
CB	HSC	CB176	_139	4909	845	2116	1046	382	4	8
CB	HSC	CB176	_140	14647	13970	28	29	2	56	290
CB	HSC	CB176	_141	1063	6	127	337	19	156	383
CB	GMDP	CB176	_142	335	19	8	251	43	11	2
CB	GMDP	CB176	_143	421	32	68	188	70	1	61
CB	GMDP	CB176	_144	24388	1154	1858	6	81	9	1542
CB	GMDP	CB176	_145	202	0	11	78	70	22	7
CB	CMP	CB176	_146	6033	13	5427	437	18	9	0
CB	CMP	CB176	_147	4642	9	1968	1530	123	202	1
CB	CMP	CB176	_148	1311	0	65	61	23	592	153

CB	MPP	CB155	_149	3326	528	1949	708	101	21	5
CB	MPP	CB155	_150	403	7	14	46	0	19	125
CB	MPP	CB155	_151	181	5	34	29	24	14	50
CB	HSC	CB155	_152	872	15	173	225	111	6	331
CB	CMP	CB155	_153	4541	103	529	901	18	2	51
CB	CMP	CB155	_154	3329	33	1969	1072	44	1	49
CB	MPP	CB154	_155	415	2	86	88	131	32	73
CB	HSC	CB154	_156	1255	8	387	163	2	56	354
CB	HSC	CB154	_157	1066	0	203	48	124	170	313
CB	CMP	CB154	_158	373	36	0	60	188	45	9
CB	CMP	CB154	_159	289	11	2	19	21	10	41
CB	MPP	CB114	_160	3222	0	14	22	101	248	1093
CB	LMPP	CB114	_161	661	23	38	220	322	32	3
CB	HSC	CB114	_162	1503	159	19	114	1117	5	33
CB	HSC	CB114	_163	3395	32	11	6	20	24	755
CB	MPP	CB110	_164	170	17	61	18	15	23	5
CB	MPP	CB110	_165	3044	2895	30	7	12	11	5
CB	MPP	CB110	_166	29539	6334	20335	363	1715	68	6
CB	MPP	CB110	_167	20278	3165	16432	124	360	21	0
CB	MPP	CB110	_168	23131	1527	13194	4241	184	78	3
CB	MPP	CB110	_169	2002	2	12	36	65	159	1121
CB	MPP	CB110	_170	9949	2	100	38	42	82	943
CB	MPP	CB110	_171	8135	1	67	247	1092	1110	5352
CB	HSC	CB110	_172	8277	69	2202	1527	3	266	30
CB	HSC	CB110	_173	1493	11	3	119	208	475	212
CB	HSC	CB110	_174	12197	1	97	1135	4054	4170	258
CB	HSC	CB110	_175	9594	5	1021	2589	194	408	76
CB	HSC	CB110	_176	1159	6	35	177	146	135	516
CB	GMDP	CB110	_177	342	190	0	9	89	44	8
CB	MPP	CB109	_178	3690	12	356	437	27	65	0
CB	MPP	CB109	_179	1628	121	554	588	198	27	0
CB	MPP	CB109	_180	15588	536	10910	1730	1427	3	53
CB	MPP	CB109	_181	3279	486	1897	749	113	0	9
CB	MPP	CB109	_182	9054	4	1208	4971	665	168	17
CB	MPP	CB109	_183	5642	0	67	374	1156	1723	1734
CB	MPP	CB109	_184	20455	5	779	6433	1005	1103	19
CB	LMPP	CB109	_185	2778	0	9	158	192	284	1911
CB	HSC	CB109	_186	17746	184	5621	5800	775	242	1
CB	HSC	CB109	_187	11675	5	115	3097	64	1890	41
CB	LMPP	CB106	_188	7288	670	3071	3507	8	18	2
CB	LMPP	CB106	_189	3171	7	733	2381	43	7	0
CB	LMPP	CB106	_190	764	8	183	316	2	8	33
CB	LMPP	CB106	_191	383	0	121	145	18	10	12
CB	LMPP	CB106	_192	9603	0	4006	5441	8	47	31
CB	LMPP	CB106	_193	5436	0	833	4431	9	79	21
CB	HSC	CB106	_194	41542	7	17949	7441	0	9	3109
CB	HSC	CB106	_195	32663	23	1857	7420	0	14	731
CB	HSC	CB106	_196	43235	24	11012	11909	0	14	451
CB	CMP	CB106	_197	12474	114	7646	4573	43	7	2
CB	CMP	CB106	_198	14026	1490	10843	70	0	8	173

CB	MPP	CB100	_199	1057	510	54	11	19	1	384
CB	MPP	CB100	_200	346	21	47	21	6	11	191
CB	MPP	CB100	_201	67947	90	1127	15482	1	1575	394
CB	HSC	CB100	_202	91018	71984	18426	177	20	27	3
CB	HSC	CB100	_203	13551	5039	7464	626	159	2	47
CB	CMP	CB100	_204	2067	10	637	1328	64	11	5
CB	CMP	CB100	_205	2557	14	1556	794	17	16	1
CB	GMDP	CB065	_206	2795	12	1226	575	755	3	169
CB	MLP	CB193	_207	103	0	0	11	59	20	9
CB	MLP	CB193	_208	206	0	0	76	98	8	15
CB	GMDP	CB193	_209	2462	307	12	937	1181	3	0
CB	GMDP	CB193	_210	2445	0	161	1644	56	196	2
CB	GMDP	CB193	_211	487	0	129	298	7	37	3
CB	GMDP	CB193	_212	627	0	0	368	193	23	30
CB	GMDP	CB193	_213	197	0	0	78	32	53	18
CB	MLP	CB192	_214	129	0	0	14	69	21	20
CB	MLP	CB192	_215	195	0	0	56	78	27	9
CB	MLP	CB192	_216	207	0	0	20	97	48	24
CB	MLP	CB192	_217	200	0	3	56	46	10	72
CB	MLP	CB192	_218	153	0	0	26	38	30	17
CB	MDP	CB192	_219	502	0	0	223	227	13	18
CB	BNKP	CB192	_220	217	0	15	90	84	24	1
CB	MLP	CB179	_221	337	0	4	8	33	11	277
CB	MLP	CB179	_222	118	0	1	21	64	10	18
CB	LMPP	CB179	_223	184	10	111	11	9	1	1
CB	LMPP	CB179	_224	115	0	10	10	13	3	75
CB	LMPP	CB179	_225	1174	1	12	6	41	15	1067
CB	LMPP	CB179	_226	579	0	5	186	306	26	48
CB	LMPP	CB179	_227	322	0	5	86	193	11	19
CB	LMPP	CB179	_228	74	0	4	9	10	24	10
CB	GMDP	CB179	_229	890	110	657	70	13	2	0
CB	GMDP	CB179	_230	1587	9	505	928	101	0	0
CB	GMDP	CB179	_231	359	23	182	31	7	4	0
CB	GMDP	CB179	_232	1252	7	550	173	521	0	0
CB	GMDP	CB179	_233	349	74	39	116	117	1	0
CB	GMDP	CB179	_234	1484	12	563	872	33	3	0
CB	GMDP	CB179	_235	120	1	45	22	14	4	28
CB	CMP	CB179	_236	1771	71	527	395	13	0	0
CB	CMP	CB179	_237	3947	36	158	2270	31	0	1
CB	CMP	CB179	_238	185	7	55	99	3	8	0
CB	CMP	CB179	_239	1223	0	36	685	77	375	2
CB	CMP	CB179	_240	939	0	31	229	604	33	1
CB	CMP	CB179	_241	1594	0	299	921	347	13	0
CB	MLP	CB177	_242	93	0	0	7	15	12	30
CB	LMPP	CB177	_243	334	0	8	31	17	5	259
CB	LMPP	CB177	_244	164	0	7	36	39	3	70
CB	LMPP	CB177	_245	174	0	4	15	8	11	127
CB	LMPP	CB177	_246	582	2	2	59	30	18	452
CB	GMDP	CB177	_247	889	13	11	11	2	2	807
CB	GMDP	CB177	_248	85	2	7	13	0	7	51
CB	GMDP	CB177	_249	205	0	5	82	50	48	13
CB	GMDP	CB177	_250	284	0	6	99	155	15	7
CB	CMP	CB177	_251	1773	181	70	99	0	1	74
CB	CMP	CB177	_252	873	0	2	167	73	127	210

CB	CMP	CB177	_253	1674	0	2	16	7	11	29
CB	CMP	CB177	_254	1041	0	1	23	66	95	584
CB	MPP	CB176	_255	3010	355	1821	464	329	0	5
CB	MPP	CB176	_256	3506	263	1669	1209	138	2	2
CB	MPP	CB176	_257	4742	2	2120	678	18	10	1
CB	MPP	CB176	_258	1264	0	0	26	160	230	839
CB	MPP	CB176	_259	949	0	3	21	27	12	840
CB	LMPP	CB176	_260	270	26	1	57	21	112	3
CB	LMPP	CB176	_261	129	0	11	78	29	10	1
CB	LMPP	CB176	_262	83	1	9	30	25	16	1
CB	LMPP	CB176	_263	177	0	12	21	17	121	5
CB	LMPP	CB176	_264	104	0	17	52	25	8	1
CB	LMPP	CB176	_265	122	0	20	39	25	35	1
CB	LMPP	CB176	_266	716	4	155	427	26	23	0
CB	LMPP	CB176	_267	170	0	20	43	31	68	5
CB	LMPP	CB176	_268	249	0	183	36	9	0	10
CB	LMPP	CB176	_269	800	0	518	99	3	24	22
CB	LMPP	CB176	_270	277	0	15	58	6	34	148
CB	LMPP	CB176	_271	124	1	77	10	5	10	7
CB	LMPP	CB176	_272	331	0	1	17	30	29	196
CB	LMPP	CB176	_273	708	0	2	14	10	79	337
CB	LMPP	CB176	_274	206	1	4	18	40	108	32
CB	LMPP	CB176	_275	548	0	2	11	23	57	362
CB	LMPP	CB176	_276	202	0	0	32	12	80	15
CB	LMPP	CB176	_277	226	0	1	107	73	32	9
CB	LMPP	CB176	_278	239	0	1	20	36	61	74
CB	LMPP	CB176	_279	36	0	1	7	13	7	7
CB	HSC	CB176	_280	4491	131	1991	231	0	2	43
CB	HSC	CB176	_281	2543	2	39	75	0	57	1163
CB	HSC	CB176	_282	1964	3	0	387	127	468	204
CB	HSC	CB176	_283	221	5	3	21	48	47	97
CB	HSC	CB176	_284	213	1	2	7	29	18	86
CB	GMDP	CB176	_285	5020	163	4388	406	9	0	0
CB	GMDP	CB176	_286	889	138	440	294	17	0	0
CB	GMDP	CB176	_287	1863	273	1512	53	21	0	1
CB	GMDP	CB176	_288	360	28	79	243	9	0	0
CB	GMDP	CB176	_289	278	10	152	93	6	6	9
CB	GMDP	CB176	_290	370	2	81	218	61	8	0
CB	CMP	CB176	_291	1628	201	1268	121	22	0	1
CB	CMP	CB176	_292	418	0	0	188	146	64	13
CB	MPP	CB155	_293	18612	6375	10498	670	12	1	2
CB	MPP	CB155	_294	360	52	111	142	38	4	2
CB	MPP	CB155	_295	351	2	6	174	7	41	53
CB	MLP	CB155	_296	94	0	0	8	7	13	26
CB	LMPP	CB155	_297	146	0	0	14	12	11	90
CB	LMPP	CB155	_298	226	0	0	9	7	10	185
CB	HSC	CB155	_299	2274	2	2	184	1587	83	387
CB	HSC	CB155	_300	960	0	3	63	338	166	381
CB	CMP	CB155	_301	389	2	56	220	52	8	1
CB	MPP	CB154	_302	2079	148	1069	653	203	3	1
CB	MPP	CB154	_303	240	0	0	27	26	34	122
CB	LMPP	CB154	_304	484	0	0	15	8	43	346
CB	HSC	CB154	_305	1263	0	2	14	347	89	794
CB	CMP	CB154	_306	136	20	43	57	14	1	0

CB	CMP	CB154	_307	334	86	1	4	8	37	30
CB	MPP	CB114	_308	3331	42	4	140	2737	0	354
CB	MPP	CB114	_309	1657	10	5	182	1403	0	17
CB	MPP	CB114	_310	841	39	5	3	16	9	135
CB	MLP	CB114	_311	254	0	0	7	79	57	109
CB	LMPP	CB114	_312	343	1	2	63	241	13	16
CB	LMPP	CB114	_313	188	0	2	39	36	26	49
CB	LMPP	CB114	_314	521	0	1	90	291	97	9
CB	HSC	CB114	_315	499	8	8	16	4	6	17
CB	HSC	CB114	_316	977	1	5	45	137	10	337
CB	CMP	CB114	_317	396	93	36	21	232	0	5
CB	CMP	CB114	_318	871	42	206	40	1	16	6
CB	CMP	CB114	_319	1256	0	335	509	8	23	1
CB	CMP	CB114	_320	583	0	0	85	93	53	109
CB	CMP	CB114	_321	657	1	1	211	87	160	9
CB	BNKP	CB114	_322	160	0	0	9	57	10	84
CB	MPP	CB110	_323	3525	3486	8	7	9	2	0
CB	MPP	CB110	_324	145	48	11	29	6	13	2
CB	MPP	CB110	_325	9542	84	156	11	4	30	2
CB	MPP	CB110	_326	9840	0	15	10	9	1	9734
CB	MPP	CB110	_327	5226	0	25	3	41	58	5028
CB	MPP	CB110	_328	752	2	6	31	499	28	172
CB	MPP	CB110	_329	2164	0	6	7	8	40	24
CB	MPP	CB110	_330	1136	0	2	8	23	31	868
CB	MLP	CB110	_331	188	0	9	38	0	9	60
CB	MLP	CB110	_332	266	0	2	19	142	20	75
CB	LMPP	CB110	_333	853	1	352	481	10	9	0
CB	LMPP	CB110	_334	77	0	9	5	16	21	22
CB	LMPP	CB110	_335	292	0	3	58	165	47	9
CB	LMPP	CB110	_336	169	0	1	18	73	48	11
CB	HSC	CB110	_337	1863	5	52	132	1	131	12
CB	HSC	CB110	_338	645	3	0	56	56	69	269
CB	GMDP	CB110	_339	337	0	7	173	138	15	0
CB	CMP	CB110	_340	1404	131	557	171	539	6	0
CB	MPP	CB109	_341	348	0	7	42	19	86	3
CB	MPP	CB109	_342	8863	4	233	1666	6780	5	88
CB	LMPP	CB109	_343	270	0	0	90	132	11	30
CB	LMPP	CB109	_344	298	0	0	15	258	8	14
CB	LMPP	CB109	_345	3053	0	2	471	2539	32	7
CB	HSC	CB109	_346	17843	2	3	1273	121	1970	135
CB	GMDP	CB109	_347	1000	0	27	267	698	7	0
CB	GMDP	CB109	_348	1323	1	14	488	803	13	4
CB	GMDP	CB109	_349	331	2	2	36	144	43	94
CB	CMP	CB109	_350	540	9	237	92	199	0	0
CB	CMP	CB109	_351	407	2	9	194	147	32	6
CB	CMP	CB109	_352	334	0	9	148	146	24	2
CB	CMP	CB109	_353	762	0	26	413	299	13	1
CB	CMP	CB109	_354	614	0	11	234	95	71	2
CB	CMP	CB109	_355	1189	0	173	882	104	9	1
CB	MPP	CB106	_356	23283	8	69	78	0	5	9
CB	MPP	CB106	_357	8874	0	35	446	0	28	51
CB	MPP	CB106	_358	2728	1	35	1194	0	257	202
CB	MPP	CB106	_359	4619	0	58	2602	4	70	46
CB	MPP	CB106	_360	36820	0	7054	18996	4	165	24

CB	MPP	CB106	_361	27528	4	2020	11332	0	221	2882
CB	MPP	CB106	_362	15250	0	18	3926	0	247	102
CB	MPP	CB106	_363	12709	0	5019	5282	0	80	774
CB	MLP	CB106	_364	416	0	12	13	0	12	305
CB	MLP	CB106	_365	886	0	69	704	1	11	95
CB	MLP	CB106	_366	236	0	0	102	19	32	82
CB	LMPP	CB106	_367	4333	1728	2307	7	0	2	9
CB	LMPP	CB106	_368	8988	6	2602	6182	17	160	2
CB	LMPP	CB106	_369	472	2	95	327	36	7	1
CB	LMPP	CB106	_370	295	0	125	87	68	0	14
CB	LMPP	CB106	_371	468	0	15	33	25	0	169
CB	LMPP	CB106	_372	1740	0	383	998	27	6	193
CB	LMPP	CB106	_373	436	0	15	316	0	13	51
CB	LMPP	CB106	_374	1039	0	22	336	0	10	23
CB	LMPP	CB106	_375	242	0	21	22	0	31	114
CB	HSC	CB106	_376	8003	0	834	3975	0	97	263
CB	HSC	CB106	_377	12597	1	54	437	0	63	18
CB	HSC	CB106	_378	8901	0	28	403	0	29	758
CB	GMDP	CB106	_379	9392	88	7870	1266	0	7	0
CB	GMDP	CB106	_380	8287	438	7565	106	0	77	0
CB	CMP	CB106	_381	21615	2694	15267	488	0	136	1
CB	CMP	CB106	_382	11912	102	9481	2147	0	10	1
CB	CMP	CB106	_383	66	15	14	22	0	8	0
CB	CMP	CB106	_384	18341	38	12977	2182	0	2	556
CB	MPP	CB100	_385	1063	788	19	13	1	1	207
CB	MPP	CB100	_386	27135	24646	2014	5	116	55	5
CB	MLP	CB100	_387	255	0	0	51	16	11	177
CB	MLP	CB100	_388	593	0	1	12	11	9	559
CB	GMDP	CB100	_389	2446	2	1377	1023	32	7	0
CB	GMDP	CB065	_390	2329	9	2225	13	5	0	11
CB	GMDP	CB065	_391	4137	1	100	3783	245	8	0
CB	GMDP	CB065	_392	1220	1	74	499	30	1	378
CB	GMDP	CB061	_393	2397	1	7	2335	27	24	1
CB	GMDP	CB061	_394	5859	3	280	5508	11	57	0
CB	GMDP	CB061	_395	4516	1	609	3857	18	31	0
CB	MLP	CB193	_396	81	0	0	10	50	12	6
CB	MLP	CB193	_397	518	0	6	50	404	42	4
CB	MLP	CB193	_398	90	0	0	17	60	8	5
CB	MLP	CB193	_399	47	0	0	12	27	7	0
CB	MLP	CB193	_400	87	0	0	14	58	0	11
CB	MLP	CB193	_401	85	0	0	11	24	5	40
CB	MLP	CB193	_402	32	0	0	3	13	7	9
CB	MLP	CB193	_403	35	0	1	0	11	9	11
CB	MDP	CB193	_404	634	2	56	18	9	0	0
CB	GMDP	CB193	_405	1354	210	835	309	0	0	0
CB	GMDP	CB193	_406	1995	7	1856	128	0	0	1
CB	GMDP	CB193	_407	370	41	17	310	0	0	0
CB	GMDP	CB193	_408	1004	817	24	124	3	1	0
CB	GMDP	CB193	_409	502	0	0	55	407	4	8
CB	BNKP	CB193	_410	2922	183	1637	233	0	0	3
CB	BNKP	CB193	_411	117	0	3	24	61	7	5
CB	BNKP	CB193	_412	765	0	0	8	0	10	16
CB	MLP	CB192	_413	112	0	0	31	41	38	1
CB	MLP	CB192	_414	1173	0	0	277	887	8	0

CB	MLP	CB192	_415	178	0	0	28	126	4	13
CB	MLP	CB192	_416	139	0	0	3	62	7	67
CB	MLP	CB192	_417	119	0	0	17	52	2	27
CB	MLP	CB192	_418	66	0	3	36	9	14	1
CB	MLP	CB192	_419	952	0	0	344	593	13	0
CB	GMDP	CB192	_420	1454	207	1204	26	0	0	0
CB	GMDP	CB192	_421	1269	0	567	472	229	0	0
CB	GMDP	CB192	_422	772	0	335	311	119	6	0
CB	GMDP	CB192	_423	1066	1	26	1022	17	0	0
CB	GMDP	CB192	_424	2754	0	1	346	2266	136	4
CB	GMDP	CB192	_425	144	0	0	17	71	6	30
CB	BNKP	CB192	_426	2425	2	661	1333	372	1	0
CB	BNKP	CB180	_427	185	1	75	79	30	0	0
CB	BNKP	CB180	_428	78	0	0	52	9	11	3
CB	BNKP	CB180	_429	121	0	0	25	46	38	4
CB	BNKP	CB180	_430	87	0	0	12	11	3	59
CB	MLP	CB179	_431	45	0	8	13	5	17	1
CB	MLP	CB179	_432	189	0	2	79	85	18	1
CB	MLP	CB179	_433	827	0	0	17	7	1	802
CB	MLP	CB179	_434	233	0	3	19	178	1	31
CB	MLP	CB179	_435	473	0	2	5	12	18	435
CB	LMPP	CB179	_436	42	0	23	7	7	0	0
CB	LMPP	CB179	_437	124	4	63	25	11	3	1
CB	LMPP	CB179	_438	65	0	28	17	13	0	0
CB	LMPP	CB179	_439	586	0	8	228	336	0	3
CB	LMPP	CB179	_440	1107	0	8	5	5	41	465
CB	GMDP	CB179	_441	721	81	595	42	0	0	0
CB	GMDP	CB179	_442	1919	82	664	1165	2	1	0
CB	GMDP	CB179	_443	2330	31	1104	1097	0	1	0
CB	GMDP	CB179	_444	3013	68	2473	470	0	0	0
CB	GMDP	CB179	_445	1751	35	1102	555	0	0	0
CB	GMDP	CB179	_446	276	20	239	11	0	1	1
CB	GMDP	CB179	_447	232	66	64	96	4	1	0
CB	GMDP	CB179	_448	277	12	251	2	10	1	0
CB	GMDP	CB179	_449	329	245	59	1	19	1	0
CB	GMDP	CB179	_450	265	8	5	10	237	0	2
CB	GMDP	CB179	_451	764	0	467	284	8	4	0
CB	GMDP	CB179	_452	468	6	105	326	27	1	0
CB	GMDP	CB179	_453	541	2	27	86	416	5	1
CB	GMDP	CB179	_454	456	0	41	404	9	0	0
CB	GMDP	CB179	_455	117	0	72	17	1	13	6
CB	CMP	CB179	_456	1801	126	138	981	2	1	1
CB	CMP	CB179	_457	2063	89	717	37	1	2	0
CB	CMP	CB179	_458	557	44	496	8	6	0	0
CB	CMP	CB179	_459	104	43	15	17	2	0	1
CB	CMP	CB179	_460	436	75	196	0	0	0	8
CB	CMP	CB179	_461	752	0	27	656	68	0	0
CB	CMP	CB179	_462	47	5	9	22	0	8	0
CB	CMP	CB179	_463	940	2	322	521	4	7	0
CB	MLP	CB177	_464	60	0	0	22	20	11	3
CB	MLP	CB177	_465	106	0	0	17	65	8	1
CB	LMPP	CB177	_466	62	0	23	12	6	2	11
CB	LMPP	CB177	_467	57	1	9	0	4	7	36
CB	LMPP	CB177	_468	194	1	3	18	19	5	138

CB	LMPP	CB177	_469	422	0	2	10	23	1	377
CB	GMDP	CB177	_470	328	205	56	9	0	0	1
CB	GMDP	CB177	_471	89	15	8	64	0	0	2
CB	GMDP	CB177	_472	161	38	31	36	0	6	4
CB	GMDP	CB177	_473	387	192	164	2	0	0	25
CB	GMDP	CB177	_474	94	0	22	7	60	0	4
CB	GMDP	CB177	_475	85	3	17	1	50	1	13
CB	CMP	CB177	_476	209	15	19	53	3	0	4
CB	CMP	CB177	_477	369	119	41	157	1	0	0
CB	CMP	CB177	_478	2041	62	1779	7	0	0	0
CB	CMP	CB177	_479	2375	78	1869	28	0	0	0
CB	CMP	CB177	_480	519	185	39	248	4	0	2
CB	CMP	CB177	_481	2069	816	299	0	0	0	13
CB	MPP	CB176	_482	16938	3525	10568	119	0	1	0
CB	MPP	CB176	_483	24577	4004	17856	185	0	1	0
CB	MPP	CB176	_484	7329	1733	5522	49	1	0	0
CB	MPP	CB176	_485	7959	481	6797	429	1	3	0
CB	MPP	CB176	_486	5899	59	3009	197	1	0	1
CB	MPP	CB176	_487	10502	6	70	23	0	63	6
CB	MPP	CB176	_488	316	0	49	99	0	3	15
CB	MPP	CB176	_489	2134	0	3	109	1	341	48
CB	MPP	CB176	_490	946	0	0	0	11	22	906
CB	LMPP	CB176	_491	2030	298	1262	376	1	1	1
CB	LMPP	CB176	_492	1295	7	1041	247	0	0	0
CB	LMPP	CB176	_493	8239	29	662	203	0	5	2
CB	LMPP	CB176	_494	571	46	480	44	0	0	0
CB	LMPP	CB176	_495	339	7	50	273	5	2	0
CB	LMPP	CB176	_496	1757	181	1567	7	0	0	0
CB	LMPP	CB176	_497	477	130	328	18	0	0	0
CB	LMPP	CB176	_498	420	200	18	191	4	2	0
CB	LMPP	CB176	_499	170	26	115	29	0	0	0
CB	LMPP	CB176	_500	246	8	128	109	1	0	0
CB	LMPP	CB176	_501	687	29	628	27	0	0	0
CB	LMPP	CB176	_502	636	30	570	4	3	9	0
CB	LMPP	CB176	_503	126	0	61	31	19	0	2
CB	LMPP	CB176	_504	228	0	9	203	10	6	0
CB	LMPP	CB176	_505	716	0	394	300	20	0	1
CB	LMPP	CB176	_506	263	0	7	213	42	0	0
CB	LMPP	CB176	_507	234	0	70	118	6	34	0
CB	LMPP	CB176	_508	346	4	181	154	0	7	0
CB	LMPP	CB176	_509	251	0	114	96	6	6	14
CB	LMPP	CB176	_510	117	1	10	2	1	49	43
CB	LMPP	CB176	_511	85	0	23	6	4	14	38
CB	LMPP	CB176	_512	273	3	0	62	31	177	0
CB	LMPP	CB176	_513	193	0	4	99	49	39	2
CB	LMPP	CB176	_514	86	0	1	23	52	9	1
CB	LMPP	CB176	_515	78	0	6	12	21	35	3
CB	LMPP	CB176	_516	97	0	0	8	79	8	1
CB	LMPP	CB176	_517	246	0	1	24	3	11	60
CB	LMPP	CB176	_518	184	0	0	14	1	12	147
CB	LMPP	CB176	_519	68	0	0	12	4	19	24
CB	LMPP	CB176	_520	90	0	0	2	19	31	36
CB	LMPP	CB176	_521	64	0	1	2	20	29	7
CB	LMPP	CB176	_522	2579	0	1	3	38	27	2490

CB	LMPP	CB176	_523	83	0	1	2	8	50	14
CB	LMPP	CB176	_524	152	0	0	0	14	53	84
CB	LMPP	CB176	_525	457	0	6	5	14	213	199
CB	LMPP	CB176	_526	110	0	0	4	17	25	62
CB	HSC	CB176	_527	6925	353	6378	124	6	0	1
CB	HSC	CB176	_528	64	0	1	2	12	24	16
CB	GMDP	CB176	_529	216	13	192	11	0	0	0
CB	GMDP	CB176	_530	920	190	588	142	0	0	0
CB	GMDP	CB176	_531	1381	45	1314	21	0	0	0
CB	GMDP	CB176	_532	594	16	347	231	0	0	0
CB	GMDP	CB176	_533	172	9	105	58	0	0	0
CB	GMDP	CB176	_534	294	86	42	163	1	0	0
CB	GMDP	CB176	_535	429	125	232	68	0	0	0
CB	GMDP	CB176	_536	740	343	232	165	0	0	0
CB	GMDP	CB176	_537	376	264	84	27	0	0	0
CB	GMDP	CB176	_538	638	17	572	49	0	0	0
CB	GMDP	CB176	_539	557	0	504	37	2	3	8
CB	GMDP	CB176	_540	43	0	0	7	13	22	1
CB	GMDP	CB176	_541	430	1	5	96	263	61	4
CB	GMDP	CB176	_542	133	0	1	14	13	95	5
CB	CMP	CB176	_543	3509	76	3385	46	2	0	0
CB	CMP	CB176	_544	6601	80	6455	19	2	0	0
CB	CMP	CB176	_545	847	27	724	93	1	1	0
CB	CMP	CB176	_546	1007	65	496	17	0	1	0
CB	CMP	CB176	_547	1886	2	1467	180	3	81	1
CB	CMP	CB176	_548	142	1	9	56	0	11	1
CB	CMP	CB176	_549	344	0	0	16	7	94	0
CB	CMP	CB176	_550	104	0	3	19	2	10	8
CB	MPP	CB155	_551	5154	2132	2645	304	3	1	2
CB	MPP	CB155	_552	183	0	2	49	95	29	5
CB	MLP	CB155	_553	88	0	15	37	12	1	5
CB	LMPP	CB155	_554	1033	0	1	16	20	6	452
CB	LMPP	CB155	_555	125	1	0	17	0	26	43
CB	HSC	CB155	_556	1480	782	688	9	0	0	0
CB	CMP	CB155	_557	339	105	181	29	0	0	0
CB	CMP	CB155	_558	1802	285	1492	15	1	0	1
CB	CMP	CB155	_559	498	5	63	360	70	0	0
CB	MPP	CB154	_560	613	39	551	20	0	0	0
CB	MPP	CB154	_561	982	0	1	2	11	45	877
CB	LMPP	CB154	_562	469	392	0	1	61	11	1
CB	LMPP	CB154	_563	87	0	34	40	13	0	0
CB	LMPP	CB154	_564	356	0	272	28	0	1	43
CB	LMPP	CB154	_565	127	0	0	48	65	9	2
CB	LMPP	CB154	_566	116	0	0	10	48	29	6
CB	LMPP	CB154	_567	147	0	1	5	8	38	80
CB	LMPP	CB154	_568	61	1	0	1	20	19	14
CB	HSC	CB154	_569	84	4	36	16	20	0	1
CB	HSC	CB154	_570	945	2	4	5	22	71	744
CB	CMP	CB154	_571	107	0	0	8	7	58	5
CB	MPP	CB114	_572	278	8	5	25	222	0	1
CB	MPP	CB114	_573	1042	5	13	24	9	1	0
CB	MPP	CB114	_574	569	0	2	27	518	1	17
CB	MPP	CB114	_575	844	0	3	34	407	3	363
CB	MLP	CB114	_576	201	0	0	19	148	31	3

CB	MLP	CB114	_577	127	1	2	6	35	30	21
CB	LMPP	CB114	_578	2848	294	378	53	0	0	1
CB	LMPP	CB114	_579	107	1	46	16	44	0	0
CB	LMPP	CB114	_580	263	0	13	135	108	2	0
CB	LMPP	CB114	_581	330	0	1	124	184	16	0
CB	LMPP	CB114	_582	295	0	5	185	68	27	0
CB	LMPP	CB114	_583	896	0	0	19	7	4	824
CB	LMPP	CB114	_584	150	0	0	61	49	4	21
CB	LMPP	CB114	_585	377	0	1	13	2	108	191
CB	HSC	CB114	_586	7792	3035	71	0	0	73	1
CB	HSC	CB114	_587	4371	670	29	0	0	17	0
CB	HSC	CB114	_588	1434	22	1	110	1280	0	0
CB	GMDP	CB114	_589	354	43	106	203	0	0	0
CB	GMDP	CB114	_590	229	106	65	57	0	0	0
CB	GMDP	CB114	_591	99	0	2	12	75	8	1
CB	GMDP	CB114	_592	766	0	1	183	541	24	6
CB	CMP	CB114	_593	125	0	0	55	15	18	6
CB	BNKP	CB114	_594	97	0	4	5	25	7	53
CB	MPP	CB110	_595	267	197	47	12	2	0	0
CB	MPP	CB110	_596	5890	254	42	2	2	77	2
CB	MPP	CB110	_597	1335	31	7	0	3	7	0
CB	MPP	CB110	_598	884	1	60	82	730	1	3
CB	MPP	CB110	_599	55	1	2	8	29	3	7
CB	MPP	CB110	_600	95	0	0	4	8	9	62
CB	MLP	CB110	_601	597	0	8	1	2	18	561
CB	MLP	CB110	_602	62	0	1	9	20	29	3
CB	MLP	CB110	_603	181	0	2	34	5	10	129
CB	LMPP	CB110	_604	135	2	85	12	22	3	6
CB	LMPP	CB110	_605	419	0	42	224	147	3	0
CB	LMPP	CB110	_606	481	0	18	45	418	0	0
CB	LMPP	CB110	_607	203	0	5	43	32	100	4
CB	LMPP	CB110	_608	69	0	0	22	19	25	0
CB	LMPP	CB110	_609	160	0	1	75	47	36	0
CB	LMPP	CB110	_610	255	0	1	76	137	39	0
CB	LMPP	CB110	_611	115	0	0	15	18	82	0
CB	LMPP	CB110	_612	123	0	0	11	33	58	1
CB	LMPP	CB110	_613	50	0	0	22	20	7	1
CB	LMPP	CB110	_614	1023	0	4	337	669	13	0
CB	LMPP	CB110	_615	234	0	0	68	47	106	6
CB	LMPP	CB110	_616	437	0	4	12	13	1	407
CB	LMPP	CB110	_617	45	1	4	17	4	7	10
CB	LMPP	CB110	_618	66	0	0	9	5	16	26
CB	LMPP	CB110	_619	282	0	1	9	0	7	253
CB	LMPP	CB110	_620	98	0	0	2	68	8	16
CB	LMPP	CB110	_621	96	0	0	2	24	8	61
CB	HSC	CB110	_622	85	10	10	2	2	3	35
CB	HSC	CB110	_623	301	0	11	23	0	7	0
CB	GMDP	CB110	_624	407	1	122	139	145	0	0
CB	GMDP	CB110	_625	137	0	14	16	107	0	0
CB	GMDP	CB110	_626	864	1	0	66	774	21	0
CB	CMP	CB110	_627	122	1	26	23	67	3	0
CB	CMP	CB110	_628	245	0	3	47	177	9	6
CB	MPP	CB109	_629	86	55	0	14	15	2	0
CB	MLP	CB109	_630	138	0	1	2	13	17	88

CB	LMPP	CB109	_631	460	0	97	288	75	0	0
CB	LMPP	CB109	_632	177	0	21	50	102	0	1
CB	LMPP	CB109	_633	88	0	23	14	46	0	1
CB	LMPP	CB109	_634	63	0	1	12	9	1	33
CB	LMPP	CB109	_635	169	0	0	44	5	28	91
CB	HSC	CB109	_636	7085	5920	7	0	0	25	0
CB	HSC	CB109	_637	3531	2985	8	1	1	10	0
CB	HSC	CB109	_638	367	1	12	70	24	0	1
CB	HSC	CB109	_639	135	1	8	28	3	10	0
CB	HSC	CB109	_640	11869	6	48	405	4	138	3
CB	GMDP	CB109	_641	681	0	40	346	291	3	0
CB	GMDP	CB109	_642	467	1	13	409	43	0	0
CB	CMP	CB109	_643	769	9	204	498	4	1	0
CB	CMP	CB109	_644	183	20	138	19	0	0	0
CB	CMP	CB109	_645	519	0	25	247	244	1	2
CB	CMP	CB109	_646	129	0	57	21	44	0	1
CB	BNKP	CB109	_647	53	0	0	21	17	15	0
CB	BNKP	CB109	_648	103	0	1	2	8	7	85
CB	MPP	CB106	_649	4188	31	38	18	0	4	1
CB	MPP	CB106	_650	18299	0	1635	2288	0	2	51
CB	LMPP	CB106	_651	7838	149	4753	465	0	1	3
CB	LMPP	CB106	_652	14510	172	9818	3944	0	3	4
CB	LMPP	CB106	_653	328	0	25	100	0	7	5
CB	LMPP	CB106	_654	3929	2	1261	2640	0	9	2
CB	LMPP	CB106	_655	220	0	14	15	0	0	86
CB	LMPP	CB106	_656	2134	0	144	422	0	6	14
CB	LMPP	CB106	_657	816	0	1	117	0	11	13
CB	LMPP	CB106	_658	1562	0	0	195	0	82	12
CB	LMPP	CB106	_659	377	0	0	167	1	60	50
CB	LMPP	CB106	_660	160	0	1	60	0	8	69
CB	LMPP	CB106	_661	291	0	0	140	0	15	124
CB	LMPP	CB106	_662	1258	0	3	53	1	48	97
CB	HSC	CB106	_663	9760	32	7454	528	0	6	2
CB	HSC	CB106	_664	8875	1	266	6666	2	72	1
CB	HSC	CB106	_665	3947	1	21	331	0	3	35
CB	HSC	CB106	_666	3630	1	25	12	0	5	98
CB	GMDP	CB106	_667	1164	15	1126	23	0	0	0
CB	GMDP	CB106	_668	6593	652	5647	35	0	0	3
CB	GMDP	CB106	_669	1660	21	1564	75	0	0	0
CB	GMDP	CB106	_670	1772	26	1678	66	0	0	0
CB	GMDP	CB106	_671	2864	34	1420	1397	5	1	4
CB	GMDP	CB106	_672	3892	94	1493	2295	0	0	0
CB	GMDP	CB106	_673	4938	654	4043	10	0	0	1
CB	GMDP	CB106	_674	3261	13	1293	1945	0	1	0
CB	GMDP	CB106	_675	5623	29	2008	3539	1	1	0
CB	GMDP	CB106	_676	1375	62	1299	9	0	0	2
CB	GMDP	CB106	_677	413	92	47	0	0	25	0
CB	GMDP	CB106	_678	1482	652	569	0	0	9	1
CB	GMDP	CB106	_679	166	39	52	0	0	50	0
CB	GMDP	CB106	_680	2216	1	123	1930	155	0	1
CB	GMDP	CB106	_681	2003	3	1062	876	58	1	3
CB	GMDP	CB106	_682	4913	5	1282	3593	3	17	4
CB	GMDP	CB106	_683	8684	2	6146	2514	0	3	9
CB	GMDP	CB106	_684	217	0	3	190	8	11	0

CB	GMDP	CB106	_685	91	0	5	30	41	1	11
CB	CMP	CB106	_686	168	24	113	7	0	2	0
CB	CMP	CB106	_687	5409	14	1992	3387	5	6	2
CB	CMP	CB106	_688	11300	93	7572	239	0	0	2
CB	CMP	CB106	_689	15393	8	4852	10456	1	0	0
CB	CMP	CB106	_690	117	97	8	12	0	0	0
CB	CMP	CB106	_691	976	788	8	1	0	9	0
CB	CMP	CB106	_692	138	15	11	4	0	12	0
CB	CMP	CB106	_693	3276	636	1142	1	0	17	1
CB	CMP	CB106	_694	141	33	47	6	0	28	0
CB	CMP	CB106	_695	887	523	188	2	0	41	0
CB	CMP	CB106	_696	1096	944	90	3	0	33	0
CB	CMP	CB106	_697	1224	69	194	0	0	19	0
CB	BNKP	CB106	_698	41	0	0	11	8	0	11
CB	MPP	CB100	_699	274	71	183	11	3	0	2
CB	MPP	CB100	_700	97	17	9	18	0	2	3
CB	MLP	CB100	_701	156	0	1	38	1	31	45
CB	HSC	CB100	_702	330	247	42	22	6	4	1
CB	HSC	CB100	_703	83419	76541	5950	0	0	154	5
CB	HSC	CB100	_704	762	102	112	0	0	2	123
CB	HSC	CB100	_705	1135	6	31	228	0	22	4
CB	GMDP	CB100	_706	559	12	536	7	3	0	0
CB	GMDP	CB100	_707	1454	16	1403	20	2	3	0
CB	GMDP	CB100	_708	1676	32	1213	412	1	5	0
CB	GMDP	CB100	_709	1481	94	1362	17	0	0	0
CB	MDP	CB065	_710	151	0	12	118	21	0	0
CB	GMDP	CB065	_711	421	17	278	122	0	0	0
CB	GMDP	CB065	_712	1122	0	62	996	64	0	0
CB	GMDP	CB065	_713	2513	1	94	1500	909	5	0
CB	GMDP	CB065	_714	1173	0	38	501	625	6	1
CB	GMDP	CB065	_715	1171	0	149	1001	21	0	0
CB	GMDP	CB065	_716	1131	0	31	633	462	5	0
CB	GMDP	CB065	_717	1598	3	832	699	5	0	30
CB	GMDP	CB061	_718	7806	224	4679	2736	0	0	1
CB	GMDP	CB061	_719	2385	13	2308	62	0	0	1
CB	GMDP	CB061	_720	682	29	484	166	0	0	0
CB	GMDP	CB061	_721	5037	8	735	4285	0	6	0
CB	GMDP	CB061	_722	9110	5	2635	6461	8	0	0
CB	GMDP	CB061	_723	2215	3	142	2049	20	0	0
CB	GMDP	CB061	_724	9524	1	20	9349	1	129	0
CB	GMDP	CB061	_725	2909	6	315	2433	0	1	19
CB	GMDP	CB061	_726	122	1	0	16	0	9	92
CB	MLP	CB193	_727	88	0	0	57	27	3	1
CB	MLP	CB193	_728	171	0	0	36	134	0	0
CB	MLP	CB193	_729	168	0	0	40	118	0	4
CB	MLP	CB193	_730	342	0	3	49	289	0	0
CB	MLP	CB193	_731	214	0	0	16	193	0	4
CB	MLP	CB193	_732	18	0	0	7	2	0	7
CB	MLP	CB193	_733	78	0	0	3	28	4	41
CB	MLP	CB193	_734	19	0	0	1	11	0	7
CB	MLP	CB193	_735	159	0	0	5	5	31	109
CB	MDP	CB193	_736	387	14	371	0	0	0	0
CB	MDP	CB193	_737	178	1	24	153	0	0	0
CB	MDP	CB193	_738	66	1	36	29	0	0	0

CB	MDP	CB193	_739	37	0	17	20	0	0	0
CB	MDP	CB193	_740	255	4	50	201	0	0	0
CB	MDP	CB193	_741	30	0	14	9	0	0	0
CB	MDP	CB193	_742	151	2	103	46	0	0	0
CB	MDP	CB193	_743	53	0	13	39	0	0	0
CB	MDP	CB193	_744	43	0	7	36	0	0	0
CB	MDP	CB193	_745	155	0	100	54	0	0	0
CB	MDP	CB193	_746	66	0	52	9	0	0	0
CB	MDP	CB193	_747	97	4	4	7	53	3	4
CB	MDP	CB193	_748	242	0	1	17	224	0	0
CB	MDP	CB193	_749	55	0	0	18	37	0	0
CB	MDP	CB193	_750	133	0	0	29	104	0	0
CB	GMDP	CB193	_751	1129	1090	19	0	0	0	0
CB	GMDP	CB193	_752	903	156	718	0	1	0	0
CB	GMDP	CB193	_753	166	54	110	1	0	0	0
CB	GMDP	CB193	_754	73	65	7	0	0	0	0
CB	GMDP	CB193	_755	1753	10	1593	2	0	0	0
CB	GMDP	CB193	_756	27	16	10	1	0	0	0
CB	GMDP	CB193	_757	193	160	32	0	0	0	0
CB	GMDP	CB193	_758	683	128	531	0	0	0	0
CB	GMDP	CB193	_759	148	0	48	98	0	0	0
CB	GMDP	CB193	_760	700	0	26	670	4	0	0
CB	GMDP	CB193	_761	252	0	44	206	0	0	0
CB	GMDP	CB193	_762	563	0	58	502	0	0	0
CB	GMDP	CB193	_763	827	0	1	334	488	1	0
CB	GMDP	CB193	_764	709	0	0	116	592	1	0
CB	GMDP	CB193	_765	814	1	1	232	577	0	0
CB	GMDP	CB193	_766	761	2	3	715	39	0	0
CB	BNKP	CB193	_767	121	0	30	86	5	0	0
CB	BNKP	CB193	_768	206	1	175	24	0	0	0
CB	BNKP	CB193	_769	42	1	11	3	0	0	8
CB	BNKP	CB193	_770	38	0	0	18	11	0	1
CB	BNKP	CB193	_771	192	0	0	32	157	0	3
CB	BNKP	CB193	_772	66	0	0	10	53	0	3
CB	BNKP	CB193	_773	190	0	0	7	182	0	0
CB	MLP	CB192	_774	642	0	0	26	6	2	157
CB	MLP	CB192	_775	63	0	0	3	42	1	15
CB	MLP	CB192	_776	63	0	0	12	32	4	5
CB	MLP	CB192	_777	136	0	0	14	115	5	0
CB	MLP	CB192	_778	4037	1073	2850	6	0	0	0
CB	MLP	CB192	_779	44	0	0	2	20	0	17
CB	MLP	CB192	_780	35	0	0	11	15	1	5
CB	MLP	CB192	_781	37	12	24	0	0	0	0
CB	MLP	CB192	_782	378	0	0	89	288	0	0
CB	MLP	CB192	_783	224	0	0	3	93	6	112
CB	MLP	CB192	_784	90	0	1	42	27	3	2
CB	MDP	CB192	_785	613	74	511	0	0	0	0
CB	MDP	CB192	_786	1961	1280	133	0	0	0	0
CB	MDP	CB192	_787	247	17	230	0	0	0	0
CB	MDP	CB192	_788	711	52	659	0	0	0	0
CB	MDP	CB192	_789	401	0	345	55	0	0	0
CB	MDP	CB192	_790	143	0	129	13	0	0	0
CB	MDP	CB192	_791	350	0	62	288	0	0	0
CB	MDP	CB192	_792	271	0	9	262	0	0	0

CB	MDP	CB192	_793	55	0	38	17	0	0	0
CB	MDP	CB192	_794	76	0	0	61	15	0	0
CB	MDP	CB192	_795	64	0	1	8	55	0	0
CB	GMDP	CB192	_796	16	9	0	0	7	0	0
CB	GMDP	CB192	_797	338	4	293	40	0	0	0
CB	GMDP	CB192	_798	577	0	415	162	0	0	0
CB	GMDP	CB192	_799	1174	0	6	1152	16	0	0
CB	GMDP	CB192	_800	217	0	0	153	62	1	0
CB	GMDP	CB192	_801	1171	0	1	355	806	2	1
CB	GMDP	CB192	_802	431	0	3	414	10	0	0
CB	GMDP	CB192	_803	48	0	0	38	8	2	0
CB	GMDP	CB192	_804	60	0	0	0	0	21	28
CB	BNKP	CB192	_805	134	0	75	55	0	1	0
CB	BNKP	CB192	_806	71	0	0	26	42	0	2
CB	BNKP	CB192	_807	102	0	0	9	81	5	1
CB	BNKP	CB192	_808	357	0	0	22	333	0	0
CB	BNKP	CB192	_809	499	1	0	174	324	0	0
CB	BNKP	CB192	_810	86	0	0	8	66	2	2
CB	BNKP	CB192	_811	46	0	6	31	9	0	0
CB	BNKP	CB192	_812	147	0	0	23	123	0	0
CB	BNKP	CB192	_813	179	0	0	18	157	0	1
CB	BNKP	CB180	_814	61	0	1	1	45	12	0
CB	BNKP	CB180	_815	254	0	0	0	10	1	241
CB	BNKP	CB180	_816	114	0	1	1	15	6	90
CB	BNKP	CB180	_817	50	0	1	0	0	32	11
CB	MLP	CB179	_818	18	0	8	2	0	7	0
CB	MLP	CB179	_819	72	0	0	22	41	5	3
CB	MLP	CB179	_820	216	0	2	13	2	0	176
CB	MLP	CB179	_821	35	0	2	2	15	15	1
CB	MLP	CB179	_822	762	0	3	0	43	1	715
CB	MLP	CB179	_823	55	0	0	0	13	0	42
CB	MLP	CB179	_824	240	0	0	4	27	3	206
CB	MLP	CB179	_825	127	0	0	1	12	1	113
CB	MLP	CB179	_826	94	0	0	2	25	4	62
CB	MLP	CB179	_827	344	0	0	0	11	4	329
CB	MLP	CB179	_828	133	0	0	0	114	0	19
CB	MLP	CB179	_829	312	0	2	0	0	17	293
CB	MLP	CB179	_830	230	0	4	5	6	10	205
CB	MLP	CB179	_831	1265	0	5	0	0	9	1251
CB	MLP	CB179	_832	28	0	1	2	0	7	18
CB	MLP	CB179	_833	97	0	1	2	2	29	61
CB	LMPP	CB179	_834	303	39	257	1	0	0	0
CB	LMPP	CB179	_835	853	456	136	0	0	0	1
CB	LMPP	CB179	_836	114	13	60	4	2	1	0
CB	LMPP	CB179	_837	51	0	21	18	5	1	2
CB	LMPP	CB179	_838	399	4	168	224	0	0	0
CB	LMPP	CB179	_839	63	1	29	6	9	0	1
CB	LMPP	CB179	_840	52	0	1	23	21	0	0
CB	LMPP	CB179	_841	246	0	1	0	0	39	110
CB	GMDP	CB179	_842	917	203	569	0	0	0	0
CB	GMDP	CB179	_843	372	102	270	0	0	0	0
CB	GMDP	CB179	_844	368	14	352	0	0	0	1
CB	GMDP	CB179	_845	351	7	344	0	0	0	0
CB	GMDP	CB179	_846	879	25	850	0	0	1	0

CB	GMDP	CB179	_847	97	82	8	0	3	3	1
CB	GMDP	CB179	_848	46	25	14	5	1	0	0
CB	GMDP	CB179	_849	53	26	25	0	0	0	0
CB	GMDP	CB179	_850	43	28	10	1	0	3	0
CB	GMDP	CB179	_851	554	98	443	3	2	2	4
CB	GMDP	CB179	_852	57	14	41	0	0	0	0
CB	GMDP	CB179	_853	862	218	641	0	0	2	0
CB	GMDP	CB179	_854	99	82	9	2	4	0	0
CB	GMDP	CB179	_855	61	20	25	0	0	3	0
CB	GMDP	CB179	_856	454	345	107	0	0	0	0
CB	GMDP	CB179	_857	43	33	7	0	0	2	0
CB	GMDP	CB179	_858	1254	68	1163	1	0	1	0
CB	GMDP	CB179	_859	216	73	0	142	0	0	0
CB	GMDP	CB179	_860	426	15	1	3	406	0	0
CB	GMDP	CB179	_861	23	0	12	8	0	2	0
CB	GMDP	CB179	_862	443	1	227	214	0	1	0
CB	GMDP	CB179	_863	264	0	11	249	0	2	0
CB	GMDP	CB179	_864	85	0	12	6	64	2	0
CB	GMDP	CB179	_865	201	2	4	57	134	3	0
CB	GMDP	CB179	_866	277	0	3	90	179	1	2
CB	GMDP	CB179	_867	1438	1	2	549	882	4	0
CB	CMP	CB179	_868	69	13	29	0	0	0	0
CB	CMP	CB179	_869	998	978	18	0	0	0	0
CB	CMP	CB179	_870	199	104	29	0	0	0	0
CB	CMP	CB179	_871	82	59	1	16	6	0	0
CB	CMP	CB179	_872	213	0	81	103	1	3	0
CB	CMP	CB179	_873	151	6	14	91	0	0	0
CB	CMP	CB179	_874	444	1	390	28	0	0	0
CB	CMP	CB179	_875	299	0	3	0	2	19	214
CB	CMP	CB179	_876	271	2	2	0	0	25	11
CB	MLP	CB177	_877	31	0	2	10	6	7	1
CB	MLP	CB177	_878	492	0	0	8	0	2	478
CB	MLP	CB177	_879	61	0	0	0	0	8	53
CB	MLP	CB177	_880	104	0	1	0	3	13	57
CB	MLP	CB177	_881	191	0	1	0	0	7	182
CB	MLP	CB177	_882	142	0	0	2	4	12	115
CB	LMPP	CB177	_883	30	2	4	9	6	0	7
CB	LMPP	CB177	_884	67	0	2	4	9	2	50
CB	GMDP	CB177	_885	911	109	558	0	0	0	0
CB	GMDP	CB177	_886	2363	320	1443	1	0	0	0
CB	GMDP	CB177	_887	2401	191	1990	1	0	0	0
CB	GMDP	CB177	_888	79	63	11	0	0	1	4
CB	GMDP	CB177	_889	81	60	21	0	0	0	0
CB	GMDP	CB177	_890	45	29	14	0	0	1	1
CB	GMDP	CB177	_891	606	329	252	0	1	0	0
CB	GMDP	CB177	_892	1024	84	813	0	0	0	0
CB	GMDP	CB177	_893	231	21	206	0	0	0	0
CB	GMDP	CB177	_894	99	90	0	0	1	0	7
CB	GMDP	CB177	_895	851	1	706	139	0	0	1
CB	GMDP	CB177	_896	85	1	49	35	0	0	0
CB	GMDP	CB177	_897	133	0	49	84	0	0	0
CB	GMDP	CB177	_898	91	0	51	39	0	1	0
CB	GMDP	CB177	_899	334	0	37	292	4	0	0
CB	GMDP	CB177	_900	526	3	11	504	4	4	0

CB	GMDP	CB177	_901	25	2	10	0	13	0	0
CB	GMDP	CB177	_902	168	0	0	30	137	0	1
CB	GMDP	CB177	_903	113	0	0	38	75	0	0
CB	GMDP	CB177	_904	125	0	4	44	76	1	0
CB	GMDP	CB177	_905	137	3	0	3	5	10	114
CB	CMP	CB177	_906	423	48	250	0	0	0	0
CB	CMP	CB177	_907	693	8	7	2	0	1	0
CB	CMP	CB177	_908	897	225	122	0	0	0	0
CB	CMP	CB177	_909	2589	2329	197	0	0	0	0
CB	CMP	CB177	_910	607	41	120	0	0	1	0
CB	CMP	CB177	_911	482	0	2	4	1	69	360
CB	CMP	CB177	_912	1119	2	0	1	3	11	818
CB	MPP	CB176	_913	231	62	8	0	0	0	0
CB	MPP	CB176	_914	1585	1577	7	0	0	0	0
CB	MPP	CB176	_915	3686	4	1	16	0	56	1
CB	MPP	CB176	_916	70	0	1	0	32	28	5
CB	MPP	CB176	_917	2131	1	0	2	0	16	28
CB	LMPP	CB176	_918	3364	351	2992	1	0	0	0
CB	LMPP	CB176	_919	609	578	13	0	0	0	1
CB	LMPP	CB176	_920	5056	110	4933	1	0	0	0
CB	LMPP	CB176	_921	4764	97	4661	0	0	0	0
CB	LMPP	CB176	_922	1065	57	998	0	0	0	0
CB	LMPP	CB176	_923	371	53	318	0	0	0	0
CB	LMPP	CB176	_924	1057	221	769	0	0	0	0
CB	LMPP	CB176	_925	222	9	4	198	5	6	0
CB	LMPP	CB176	_926	27	0	13	8	0	5	0
CB	LMPP	CB176	_927	215	4	168	43	0	0	0
CB	LMPP	CB176	_928	43	0	15	2	24	0	1
CB	LMPP	CB176	_929	23	0	11	0	1	8	3
CB	LMPP	CB176	_930	41	0	1	25	12	3	0
CB	LMPP	CB176	_931	21	0	0	11	10	0	0
CB	LMPP	CB176	_932	44	0	1	22	20	0	0
CB	LMPP	CB176	_933	205	1	1	87	114	0	1
CB	LMPP	CB176	_934	31	0	1	10	20	0	0
CB	LMPP	CB176	_935	354	1	2	333	17	1	0
CB	LMPP	CB176	_936	183	1	6	116	58	2	0
CB	LMPP	CB176	_937	29	0	0	14	15	0	0
CB	LMPP	CB176	_938	76	1	1	62	9	3	0
CB	LMPP	CB176	_939	232	0	0	32	1	41	3
CB	LMPP	CB176	_940	105	0	1	28	5	59	4
CB	LMPP	CB176	_941	55	0	0	26	2	26	1
CB	LMPP	CB176	_942	328	0	1	44	4	30	3
CB	LMPP	CB176	_943	64	0	0	11	0	51	1
CB	LMPP	CB176	_944	495	0	1	8	3	6	473
CB	LMPP	CB176	_945	19	0	0	2	7	10	0
CB	LMPP	CB176	_946	57	1	0	6	0	23	11
CB	LMPP	CB176	_947	148	0	6	4	0	7	70
CB	LMPP	CB176	_948	1052	0	0	0	2	45	866
CB	LMPP	CB176	_949	276	0	0	2	5	32	232
CB	LMPP	CB176	_950	230	0	1	0	1	8	220
CB	LMPP	CB176	_951	140	0	0	0	0	30	50
CB	LMPP	CB176	_952	172	0	4	2	0	68	86
CB	LMPP	CB176	_953	159	0	2	0	1	23	123
CB	LMPP	CB176	_954	503	0	0	5	3	52	339

CB	LMPP	CB176	_955	542	0	0	1	6	22	428
CB	LMPP	CB176	_956	48	0	0	0	3	23	22
CB	LMPP	CB176	_957	176	0	5	0	0	39	7
CB	LMPP	CB176	_958	548	1	0	0	1	24	118
CB	LMPP	CB176	_959	114	0	1	3	4	24	81
CB	LMPP	CB176	_960	57	0	0	3	0	10	40
CB	HSC	CB176	_961	7225	6148	628	1	0	0	3
CB	HSC	CB176	_962	51	3	2	13	0	16	2
CB	HSC	CB176	_963	1963	0	0	11	0	11	1
CB	HSC	CB176	_964	2631	2	1	1	0	22	52
CB	GMDP	CB176	_965	2370	12	2355	3	0	0	0
CB	GMDP	CB176	_966	522	31	491	0	0	0	0
CB	GMDP	CB176	_967	303	80	222	1	0	0	0
CB	GMDP	CB176	_968	899	29	870	0	0	0	0
CB	GMDP	CB176	_969	492	180	311	0	0	0	0
CB	GMDP	CB176	_970	999	67	932	0	0	0	0
CB	GMDP	CB176	_971	2648	200	2441	0	0	0	0
CB	GMDP	CB176	_972	988	217	770	0	0	0	0
CB	GMDP	CB176	_973	2827	580	2222	0	0	0	0
CB	GMDP	CB176	_974	2154	13	2140	1	0	0	0
CB	GMDP	CB176	_975	2283	248	2035	0	0	0	0
CB	GMDP	CB176	_976	619	172	444	3	0	0	0
CB	GMDP	CB176	_977	545	80	465	0	0	0	0
CB	GMDP	CB176	_978	518	284	170	0	0	0	0
CB	GMDP	CB176	_979	1129	43	1086	0	0	0	0
CB	GMDP	CB176	_980	1122	29	1093	0	0	0	0
CB	GMDP	CB176	_981	40	14	25	0	0	0	1
CB	GMDP	CB176	_982	961	23	935	3	0	0	0
CB	GMDP	CB176	_983	923	22	901	0	0	0	0
CB	GMDP	CB176	_984	865	41	824	0	0	0	0
CB	GMDP	CB176	_985	2019	152	1674	0	0	0	0
CB	GMDP	CB176	_986	2521	18	2502	0	0	0	0
CB	GMDP	CB176	_987	139	35	96	4	0	0	1
CB	GMDP	CB176	_988	266	126	132	0	0	1	0
CB	GMDP	CB176	_989	94	78	13	0	0	0	0
CB	GMDP	CB176	_990	127	18	108	0	0	0	0
CB	GMDP	CB176	_991	580	312	262	0	0	0	0
CB	GMDP	CB176	_992	556	45	508	0	0	0	0
CB	GMDP	CB176	_993	1037	478	555	0	0	0	0
CB	GMDP	CB176	_994	642	306	330	0	0	0	0
CB	GMDP	CB176	_995	232	193	39	0	0	0	0
CB	GMDP	CB176	_996	1589	347	1237	0	0	0	0
CB	GMDP	CB176	_997	1822	720	1050	0	0	0	0
CB	GMDP	CB176	_998	890	676	213	0	0	0	1
CB	GMDP	CB176	_999	136	59	76	0	1	0	0
CB	GMDP	CB176	_1000	272	150	120	0	0	0	1
CB	GMDP	CB176	_1001	465	91	371	0	0	0	0
CB	GMDP	CB176	_1002	750	200	549	0	0	0	0
CB	GMDP	CB176	_1003	74	37	32	0	0	0	0
CB	GMDP	CB176	_1004	150	84	66	0	0	0	0
CB	GMDP	CB176	_1005	1241	230	979	0	0	0	0
CB	GMDP	CB176	_1006	30	14	15	0	0	0	0
CB	GMDP	CB176	_1007	540	215	284	0	0	0	0
CB	GMDP	CB176	_1008	326	78	246	0	0	0	0

CB	GMDP	CB176	_1009	1242	441	801	0	0	0	0
CB	GMDP	CB176	_1010	179	153	24	0	0	0	0
CB	GMDP	CB176	_1011	112	6	67	39	0	0	0
CB	GMDP	CB176	_1012	961	2	584	375	0	0	0
CB	GMDP	CB176	_1013	306	1	2	79	216	6	2
CB	GMDP	CB176	_1014	88	1	1	74	11	0	0
CB	GMDP	CB176	_1015	59	0	0	4	37	18	0
CB	GMDP	CB176	_1016	83	0	1	5	66	11	0
CB	GMDP	CB176	_1017	34	0	0	6	15	8	5
CB	CMP	CB176	_1018	4456	282	3690	0	0	0	0
CB	CMP	CB176	_1019	451	296	66	0	0	0	1
CB	CMP	CB176	_1020	50	24	22	1	0	0	0
CB	CMP	CB176	_1021	492	107	250	0	0	0	0
CB	CMP	CB176	_1022	3293	48	3020	0	0	0	0
CB	CMP	CB176	_1023	679	410	190	0	0	0	0
CB	CMP	CB176	_1024	395	229	95	1	0	0	0
CB	CMP	CB176	_1025	767	122	536	0	0	0	1
CB	CMP	CB176	_1026	6146	310	5478	0	0	0	0
CB	CMP	CB176	_1027	363	78	18	0	0	0	1
CB	CMP	CB176	_1028	1131	4	304	47	0	0	0
CB	CMP	CB176	_1029	38	3	18	12	1	4	0
CB	CMP	CB176	_1030	362	0	287	42	0	0	0
CB	CMP	CB176	_1031	190	4	77	9	0	0	0
CB	CMP	CB176	_1032	1063	0	0	26	2	33	0
CB	CMP	CB176	_1033	308	0	1	2	1	180	38
CB	MPP	CB155	_1034	4977	125	88	5	0	0	4
CB	MLP	CB155	_1035	740	0	0	3	3	15	641
CB	LMPP	CB155	_1036	826	95	728	2	0	0	0
CB	LMPP	CB155	_1037	539	75	458	4	0	0	0
CB	LMPP	CB155	_1038	141	1	127	12	0	0	0
CB	LMPP	CB155	_1039	241	1	226	14	0	0	0
CB	LMPP	CB155	_1040	48	0	0	33	8	5	2
CB	LMPP	CB155	_1041	100	0	0	11	74	6	3
CB	HSC	CB155	_1042	8544	8511	16	1	0	4	0
CB	HSC	CB155	_1043	3068	2379	677	5	0	3	0
CB	HSC	CB155	_1044	1408	932	474	1	0	0	0
CB	HSC	CB155	_1045	182	5	2	136	38	1	0
CB	CMP	CB155	_1046	1649	561	1056	0	0	0	0
CB	CMP	CB155	_1047	51	0	0	1	6	19	16
CB	MPP	CB154	_1048	280	265	13	0	0	0	0
CB	MPP	CB154	_1049	82	0	0	4	31	34	5
CB	MPP	CB154	_1050	34	0	1	2	18	3	9
CB	MLP	CB154	_1051	56	1	0	1	0	10	43
CB	LMPP	CB154	_1052	65	0	1	1	26	22	1
CB	LMPP	CB154	_1053	49	0	4	6	10	13	4
CB	LMPP	CB154	_1054	146	0	0	4	2	45	37
CB	LMPP	CB154	_1055	304	1	3	6	6	73	122
CB	CMP	CB154	_1056	2969	433	2533	2	0	0	0
CB	CMP	CB154	_1057	981	214	13	0	0	0	1
CB	CMP	CB154	_1058	185	0	0	4	4	108	20
CB	MLP	CB114	_1059	24	0	0	7	3	1	11
CB	LMPP	CB114	_1060	952	565	217	0	0	3	0
CB	LMPP	CB114	_1061	336	264	12	0	0	2	0
CB	LMPP	CB114	_1062	64	46	13	0	0	0	1

CB	LMPP	CB114	_1063	69	0	46	19	0	0	0
CB	LMPP	CB114	_1064	198	0	33	159	0	0	5
CB	LMPP	CB114	_1065	141	0	2	67	69	1	1
CB	LMPP	CB114	_1066	26	0	0	11	12	1	1
CB	LMPP	CB114	_1067	56	0	1	19	1	5	13
CB	HSC	CB114	_1068	2388	23	8	0	0	1	0
CB	HSC	CB114	_1069	1372	101	5	27	1	4	0
CB	HSC	CB114	_1070	1533	659	6	0	1	23	1
CB	HSC	CB114	_1071	222	2	7	1	2	0	14
CB	GMDP	CB114	_1072	941	928	11	0	0	0	0
CB	GMDP	CB114	_1073	320	34	276	5	0	0	0
CB	GMDP	CB114	_1074	261	57	0	202	1	0	0
CB	GMDP	CB114	_1075	188	0	130	58	0	0	0
CB	GMDP	CB114	_1076	41	0	1	12	28	0	0
CB	BNKP	CB114	_1077	24	0	15	8	0	0	0
CB	BNKP	CB114	_1078	239	0	4	204	28	1	1
CB	BNKP	CB114	_1079	23	0	0	6	7	9	0
CB	MPP	CB110	_1080	63	7	3	0	4	3	42
CB	MPP	CB110	_1081	84	0	7	20	5	3	4
CB	MLP	CB110	_1082	56	0	2	8	39	0	0
CB	MLP	CB110	_1083	39	0	0	0	24	13	2
CB	LMPP	CB110	_1084	253	237	10	0	0	0	0
CB	LMPP	CB110	_1085	836	26	805	1	0	1	0
CB	LMPP	CB110	_1086	122	15	4	1	98	0	0
CB	LMPP	CB110	_1087	150	0	47	102	1	0	0
CB	LMPP	CB110	_1088	259	0	15	4	0	1	225
CB	LMPP	CB110	_1089	123	0	1	72	46	4	0
CB	LMPP	CB110	_1090	56	0	1	15	31	6	1
CB	LMPP	CB110	_1091	135	0	1	16	114	3	0
CB	LMPP	CB110	_1092	89	0	2	19	65	2	0
CB	LMPP	CB110	_1093	85	0	3	8	0	0	67
CB	LMPP	CB110	_1094	38	0	1	0	10	26	1
CB	LMPP	CB110	_1095	54	0	1	1	3	17	31
CB	LMPP	CB110	_1096	34	0	3	0	2	13	13
CB	LMPP	CB110	_1097	53	0	1	0	2	22	25
CB	HSC	CB110	_1098	3473	3050	1	0	0	78	1
CB	HSC	CB110	_1099	85	6	7	6	0	5	9
CB	HSC	CB110	_1100	87	0	5	5	4	7	10
CB	GMDP	CB110	_1101	371	23	347	0	0	0	0
CB	GMDP	CB110	_1102	92	15	77	0	0	0	0
CB	GMDP	CB110	_1103	353	4	314	31	2	0	0
CB	GMDP	CB110	_1104	162	1	123	37	1	0	0
CB	GMDP	CB110	_1105	127	0	0	7	116	2	2
CB	GMDP	CB110	_1106	273	1	4	8	259	0	0
CB	GMDP	CB110	_1107	177	1	4	6	132	29	3
CB	GMDP	CB110	_1108	149	0	4	1	123	17	2
CB	GMDP	CB110	_1109	24	0	0	0	10	0	13
CB	CMP	CB110	_1110	570	48	522	0	0	0	0
CB	MPP	CB109	_1111	1795	586	48	0	0	1	0
CB	MPP	CB109	_1112	340	280	46	4	2	0	0
CB	MPP	CB109	_1113	2405	1944	48	1	0	1	0
CB	MPP	CB109	_1114	43	21	3	6	8	0	0
CB	MPP	CB109	_1115	345	6	82	172	3	1	1
CB	MPP	CB109	_1116	27	0	3	12	7	0	0

CB	MLP	CB109	_1117	41	0	0	8	32	0	1
CB	MLP	CB109	_1118	156	0	1	6	3	40	65
CB	MLP	CB109	_1119	58	0	0	1	3	10	32
CB	LMPP	CB109	_1120	80	0	38	42	0	0	0
CB	LMPP	CB109	_1121	79	0	2	9	65	2	0
CB	LMPP	CB109	_1122	78	0	3	14	61	0	0
CB	LMPP	CB109	_1123	214	0	0	91	121	0	0
CB	LMPP	CB109	_1124	48	0	0	1	2	25	20
CB	HSC	CB109	_1125	81	19	25	6	0	0	0
CB	HSC	CB109	_1126	59	0	26	9	0	0	0
CB	HSC	CB109	_1127	59	3	8	6	4	3	10
CB	HSC	CB109	_1128	35	0	1	10	7	1	1
CB	HSC	CB109	_1129	41	0	6	8	7	2	0
CB	GMDP	CB109	_1130	125	81	44	0	0	0	0
CB	GMDP	CB109	_1131	56	43	10	0	0	0	0
CB	GMDP	CB109	_1132	175	9	166	0	0	0	0
CB	GMDP	CB109	_1133	129	0	33	96	0	0	0
CB	GMDP	CB109	_1134	1899	4	1658	236	0	0	0
CB	GMDP	CB109	_1135	101	0	78	21	0	0	0
CB	GMDP	CB109	_1136	99	5	63	31	0	0	0
CB	GMDP	CB109	_1137	489	0	1	13	475	0	0
CB	GMDP	CB109	_1138	140	0	5	72	63	0	0
CB	GMDP	CB109	_1139	155	0	4	14	136	0	0
CB	GMDP	CB109	_1140	135	0	1	12	119	1	0
CB	GMDP	CB109	_1141	101	0	0	91	8	0	0
CB	CMP	CB109	_1142	147	136	9	0	0	0	0
CB	CMP	CB109	_1143	103	14	86	0	0	0	0
CB	BNKP	CB109	_1144	94	0	2	2	68	1	20
CB	BNKP	CB109	_1145	68	0	4	1	53	3	7
CB	MPP	CB106	_1146	1083	15	11	1	0	1	0
CB	MPP	CB106	_1147	5193	1811	2396	2	0	1	2
CB	MPP	CB106	_1148	107	0	42	39	0	0	3
CB	MPP	CB106	_1149	1552	0	7	0	0	0	143
CB	MLP	CB106	_1150	706	694	12	0	0	0	0
CB	MLP	CB106	_1151	505	0	172	226	0	1	2
CB	MLP	CB106	_1152	162	0	38	0	1	0	115
CB	MLP	CB106	_1153	57	0	0	14	0	6	18
CB	MLP	CB106	_1154	626	1	3	9	0	4	545
CB	MLP	CB106	_1155	43	0	0	0	0	23	20
CB	MLP	CB106	_1156	486	0	0	0	0	7	392
CB	MLP	CB106	_1157	117	0	0	0	0	7	107
CB	MLP	CB106	_1158	745	3	0	0	0	7	646
CB	LMPP	CB106	_1159	2066	943	1063	1	0	0	0
CB	LMPP	CB106	_1160	2728	2089	633	1	0	0	0
CB	LMPP	CB106	_1161	40	0	20	20	0	0	0
CB	LMPP	CB106	_1162	8981	5	5408	3565	0	0	0
CB	LMPP	CB106	_1163	3070	6	1519	1529	4	1	1
CB	LMPP	CB106	_1164	3674	2	1134	2534	1	1	1
CB	LMPP	CB106	_1165	1817	0	965	730	6	0	3
CB	LMPP	CB106	_1166	23	0	12	11	0	0	0
CB	LMPP	CB106	_1167	1986	0	324	1659	0	0	0
CB	LMPP	CB106	_1168	5764	1	2254	3488	0	0	4
CB	LMPP	CB106	_1169	117	0	0	46	51	0	5
CB	LMPP	CB106	_1170	141	0	0	106	0	20	3

CB	LMPP	CB106	_1171	258	0	1	72	0	16	4
CB	LMPP	CB106	_1172	761	4	4	8	0	1	697
CB	LMPP	CB106	_1173	2062	0	4	42	0	0	1345
CB	LMPP	CB106	_1174	158	0	0	17	0	4	99
CB	LMPP	CB106	_1175	278	0	0	12	0	1	37
CB	LMPP	CB106	_1176	256	0	0	22	3	2	222
CB	LMPP	CB106	_1177	128	0	0	116	0	3	9
CB	LMPP	CB106	_1178	342	0	1	21	0	4	157
CB	LMPP	CB106	_1179	264	0	0	4	0	8	199
CB	HSC	CB106	_1180	2926	0	64	127	0	0	1
CB	GMDP	CB106	_1181	425	27	377	0	0	1	1
CB	GMDP	CB106	_1182	928	131	794	2	0	1	0
CB	GMDP	CB106	_1183	46	18	27	0	0	0	0
CB	GMDP	CB106	_1184	2803	1355	1222	0	0	0	0
CB	GMDP	CB106	_1185	6491	1599	4669	5	0	2	2
CB	GMDP	CB106	_1186	483	16	462	2	0	1	0
CB	GMDP	CB106	_1187	164	21	140	3	0	0	0
CB	GMDP	CB106	_1188	792	113	308	0	0	0	0
CB	GMDP	CB106	_1189	782	763	5	13	1	0	0
CB	GMDP	CB106	_1190	673	666	0	7	0	0	0
CB	GMDP	CB106	_1191	350	3	324	18	0	2	0
CB	GMDP	CB106	_1192	2437	3	948	1478	0	1	1
CB	GMDP	CB106	_1193	2104	5	1001	1095	0	0	1
CB	GMDP	CB106	_1194	1715	1	287	1425	0	1	0
CB	GMDP	CB106	_1195	29	1	11	16	0	0	0
CB	GMDP	CB106	_1196	2240	6	1401	828	0	0	0
CB	GMDP	CB106	_1197	73	3	11	0	0	11	0
CB	CMP	CB106	_1198	1539	1243	259	2	0	0	0
CB	CMP	CB106	_1199	2363	2006	302	1	0	0	0
CB	CMP	CB106	_1200	2144	2042	63	0	0	0	1
CB	CMP	CB106	_1201	6461	4774	1277	0	0	0	1
CB	CMP	CB106	_1202	844	798	46	0	0	0	0
CB	CMP	CB106	_1203	293	283	10	0	0	0	0
CB	CMP	CB106	_1204	42	0	26	7	0	0	1
CB	BNKP	CB106	_1205	603	1	49	553	0	0	0
CB	BNKP	CB106	_1206	402	0	92	308	1	0	0
CB	BNKP	CB106	_1207	507	2	437	67	0	0	1
CB	BNKP	CB106	_1208	2565	0	10	0	0	0	2353
CB	BNKP	CB106	_1209	46	0	0	8	0	26	5
CB	BNKP	CB106	_1210	695	0	0	109	0	2	12
CB	BNKP	CB106	_1211	432	1	2	6	0	17	271
CB	BNKP	CB106	_1212	100	0	1	0	0	9	89
CB	MPP	CB100	_1213	534	199	316	6	1	1	2
CB	MPP	CB100	_1214	58	44	10	0	0	0	0
CB	MPP	CB100	_1215	246	98	138	1	1	0	1
CB	MPP	CB100	_1216	37	11	14	5	1	1	2
CB	MPP	CB100	_1217	203	135	60	1	3	0	0
CB	MPP	CB100	_1218	232	1	1	59	0	7	1
CB	MLP	CB100	_1219	58	0	0	3	0	9	44
CB	MLP	CB100	_1220	31	0	1	1	0	7	20
CB	HSC	CB100	_1221	917	385	526	2	1	0	1
CB	HSC	CB100	_1222	37	10	19	4	1	0	1
CB	HSC	CB100	_1223	354	315	27	2	2	1	4
CB	GMDP	CB100	_1224	1003	15	980	0	0	0	0

CB	GMDP	CB100	_1225	64	35	28	0	0	0	0
CB	GMDP	CB100	_1226	68	31	26	1	0	6	0
CB	GMDP	CB100	_1227	541	72	459	1	0	1	0
CB	CMP	CB100	_1228	151	106	14	0	0	1	0
CB	CMP	CB100	_1229	1176	1090	13	1	0	1	0
CB	CMP	CB100	_1230	258	100	93	0	0	2	0
CB	CMP	CB100	_1231	128	111	16	0	0	0	0
CB	CMP	CB100	_1232	150	134	11	0	0	0	0
CB	BNKP	CB100	_1233	199	0	6	0	0	10	173
CB	MDP	CB065	_1234	1325	66	1259	0	0	0	0
CB	MDP	CB065	_1235	674	9	664	1	0	0	0
CB	MDP	CB065	_1236	192	7	185	0	0	0	0
CB	MDP	CB065	_1237	339	32	307	0	0	0	0
CB	MDP	CB065	_1238	124	9	115	0	0	0	0
CB	MDP	CB065	_1239	38	0	8	30	0	0	0
CB	MDP	CB065	_1240	108	0	0	9	99	0	0
CB	MDP	CB065	_1241	131	1	0	97	33	0	0
CB	MDP	CB065	_1242	29	0	1	8	20	0	0
CB	MDP	CB065	_1243	184	0	1	176	7	0	0
CB	MDP	CB065	_1244	182	0	0	21	161	0	0
CB	MDP	CB065	_1245	441	0	0	121	320	0	0
CB	MDP	CB065	_1246	115	1	2	95	17	0	0
CB	MDP	CB065	_1247	280	0	0	32	248	0	0
CB	GMDP	CB065	_1248	3018	126	2882	0	0	0	0
CB	GMDP	CB065	_1249	164	0	39	125	0	0	0
CB	GMDP	CB065	_1250	311	1	131	179	0	0	0
CB	GMDP	CB065	_1251	409	2	322	85	0	0	0
CB	GMDP	CB065	_1252	300	0	0	215	85	0	0
CB	CDP	CB065	_1253	13	0	0	3	10	0	0
CB	CDP	CB065	_1254	6	0	0	3	3	0	0
CB	MDP	CB061	_1255	238	1	224	13	0	0	0
CB	MDP	CB061	_1256	203	1	173	29	0	0	0
CB	MDP	CB061	_1257	1075	1	554	520	0	0	0
CB	MDP	CB061	_1258	1360	3	151	1206	0	0	0
CB	MDP	CB061	_1259	281	0	105	176	0	0	0
CB	MDP	CB061	_1260	445	1	75	369	0	0	0
CB	MDP	CB061	_1261	209	2	50	157	0	0	0
CB	MDP	CB061	_1262	2882	1	157	2724	0	0	0
CB	MDP	CB061	_1263	722	0	367	355	0	0	0
CB	MDP	CB061	_1264	293	2	129	162	0	0	0
CB	MDP	CB061	_1265	469	1	50	418	0	0	0
CB	MDP	CB061	_1266	401	2	296	91	0	0	0
CB	MDP	CB061	_1267	2139	0	14	2125	0	0	0
CB	MDP	CB061	_1268	1266	0	503	763	0	0	0
CB	MDP	CB061	_1269	655	4	238	413	0	0	0
CB	MDP	CB061	_1270	251	1	216	34	0	0	0
CB	MDP	CB061	_1271	449	0	327	39	0	0	0
CB	MDP	CB061	_1272	49	0	38	11	0	0	0
CB	MDP	CB061	_1273	163	0	0	82	81	0	0
CB	MDP	CB061	_1274	156	0	0	77	77	2	0
CB	MDP	CB061	_1275	20	0	2	9	9	0	0
CB	MDP	CB061	_1276	46	0	0	18	0	28	0
CB	MDP	CB061	_1277	267	0	0	228	0	39	0
CB	GMDP	CB061	_1278	122	77	40	5	0	0	0

CB	GMDP	CB061	_1279	2556	19	97	0	0	2	1
CB	GMDP	CB061	_1280	740	282	192	0	0	0	0
CB	GMDP	CB061	_1281	1042	1	617	424	0	0	0
CB	GMDP	CB061	_1282	6467	1	2333	4133	0	0	0
CB	GMDP	CB061	_1283	5976	2	4830	1144	0	0	0
CB	GMDP	CB061	_1284	534	1	15	507	0	0	5
CB	GMDP	CB061	_1285	1964	0	13	1945	0	6	0
CB	GMDP	CB061	_1286	52	0	13	39	0	0	0
CB	GMDP	CB061	_1287	630	0	0	603	0	25	0
CB	GMDP	CB061	_1288	81	0	0	7	0	0	29
CB	GMDP	CB061	_1289	100	1	0	44	0	0	38
CB	CDP	CB061	_1290	20	0	0	13	0	7	0
CB	CDP	CB061	_1291	17	0	1	13	0	3	0
CB	MDP	CB048	_1292	47	0	32	15	0	0	0
CB	MDP	CB048	_1293	28	0	10	18	0	0	0
CB	MDP	CB048	_1294	21	0	0	10	11	0	0
CB	MDP	CB048	_1295	30	0	2	13	15	0	0
CB	MLP	CB193	_1296	24	0	5	11	0	4	4
CB	MLP	CB193	_1297	33	0	0	26	5	0	2
CB	MLP	CB193	_1298	14	0	0	7	3	1	1
CB	MLP	CB193	_1299	23	0	0	20	0	0	2
CB	MLP	CB193	_1300	34	0	0	0	30	1	3
CB	MLP	CB193	_1301	301	0	0	0	293	0	6
CB	MLP	CB193	_1302	78	0	0	4	0	5	64
CB	MLP	CB193	_1303	87	0	0	0	0	0	86
CB	MLP	CB193	_1304	12	0	0	0	0	4	7
CB	MLP	CB193	_1305	13	0	0	1	0	0	11
CB	MLP	CB193	_1306	14	0	0	0	0	4	7
CB	MLP	CB193	_1307	26	0	0	3	1	0	22
CB	MDP	CB193	_1308	15	15	0	0	0	0	0
CB	MDP	CB193	_1309	154	154	0	0	0	0	0
CB	MDP	CB193	_1310	32	0	29	3	0	0	0
CB	MDP	CB193	_1311	400	6	389	4	0	0	0
CB	MDP	CB193	_1312	351	4	343	1	0	0	0
CB	MDP	CB193	_1313	15	0	14	0	0	0	0
CB	MDP	CB193	_1314	46	6	38	1	0	0	0
CB	MDP	CB193	_1315	44	1	36	5	0	0	0
CB	MDP	CB193	_1316	177	2	0	175	0	0	0
CB	MDP	CB193	_1317	25	0	1	24	0	0	0
CB	MDP	CB193	_1318	20	0	1	16	0	0	0
CB	MDP	CB193	_1319	327	3	4	319	0	0	0
CB	MDP	CB193	_1320	69	1	6	53	0	0	0
CB	MDP	CB193	_1321	47	0	0	47	0	0	0
CB	MDP	CB193	_1322	10	0	0	9	0	0	0
CB	MDP	CB193	_1323	22	0	0	21	0	0	0
CB	MDP	CB193	_1324	71	1	6	63	0	0	0
CB	MDP	CB193	_1325	97	1	3	91	0	0	0
CB	MDP	CB193	_1326	52	0	0	51	0	0	0
CB	MDP	CB193	_1327	14	0	0	3	9	0	0
CB	MDP	CB193	_1328	180	0	0	0	176	0	4
CB	MDP	CB193	_1329	76	0	0	0	75	0	0
CB	MDP	CB193	_1330	114	0	0	0	114	0	0
CB	MDP	CB193	_1331	210	0	0	2	206	0	0
CB	MDP	CB193	_1332	151	0	0	1	148	0	0

CB	MDP	CB193	_1333	27	0	0	0	26	0	0
CB	GMDP	CB193	_1334	30	28	0	0	1	0	0
CB	GMDP	CB193	_1335	388	386	1	0	0	0	0
CB	GMDP	CB193	_1336	414	410	2	0	0	0	0
CB	GMDP	CB193	_1337	182	176	0	1	4	0	0
CB	GMDP	CB193	_1338	18	0	16	1	0	0	0
CB	GMDP	CB193	_1339	24	0	0	12	0	1	4
CB	GMDP	CB193	_1340	32	0	0	0	28	1	1
CB	GMDP	CB193	_1341	16	0	1	1	0	3	7
CB	CDP	CB193	_1342	3	0	0	3	0	0	0
CB	CDP	CB193	_1343	6	0	0	3	0	0	0
CB	CDP	CB193	_1344	4	0	0	3	0	1	0
CB	CDP	CB193	_1345	7	0	0	7	0	0	0
CB	CDP	CB193	_1346	4	0	0	3	0	0	0
CB	CDP	CB193	_1347	3	0	0	3	0	0	0
CB	CDP	CB193	_1348	6	0	0	6	0	0	0
CB	CDP	CB193	_1349	3	0	0	3	0	0	0
CB	CDP	CB193	_1350	4	0	0	0	0	4	0
CB	CDP	CB193	_1351	4	0	0	0	0	4	0
CB	CDP	CB193	_1352	4	0	0	0	0	4	0
CB	BNKP	CB193	_1353	18	0	0	1	17	0	0
CB	BNKP	CB193	_1354	18	0	0	0	10	4	3
CB	BNKP	CB193	_1355	16	0	0	0	11	0	4
CB	BNKP	CB193	_1356	56	0	0	0	56	0	0
CB	BNKP	CB193	_1357	16	0	0	0	15	0	1
CB	BNKP	CB193	_1358	187	0	0	1	186	0	0
CB	BNKP	CB193	_1359	16	0	0	0	0	0	16
CB	BNKP	CB193	_1360	14	0	0	0	0	0	14
CB	BNKP	CB193	_1361	190	0	0	0	0	0	179
CB	BNKP	CB193	_1362	16	0	0	1	0	0	15
CB	BNKP	CB193	_1363	246	0	0	0	0	0	245
CB	BNKP	CB193	_1364	13	0	0	0	0	0	13
CB	MLP	CB192	_1365	10	0	8	0	0	0	0
CB	MLP	CB192	_1366	24	0	0	1	12	1	5
CB	MLP	CB192	_1367	54	0	0	4	3	0	32
CB	MLP	CB192	_1368	83	0	1	78	4	0	0
CB	MLP	CB192	_1369	46	0	0	3	0	3	28
CB	MLP	CB192	_1370	18	1	0	4	1	2	8
CB	MDP	CB192	_1371	89	89	0	0	0	0	0
CB	MDP	CB192	_1372	35	33	0	2	0	0	0
CB	MDP	CB192	_1373	485	0	479	5	0	0	0
CB	MDP	CB192	_1374	37	0	37	0	0	0	0
CB	MDP	CB192	_1375	34	0	34	0	0	0	0
CB	MDP	CB192	_1376	149	0	149	0	0	0	0
CB	MDP	CB192	_1377	119	0	119	0	0	0	0
CB	MDP	CB192	_1378	142	0	141	0	0	0	0
CB	MDP	CB192	_1379	17	0	17	0	0	0	0
CB	MDP	CB192	_1380	15	0	14	1	0	0	0
CB	MDP	CB192	_1381	70	0	70	0	0	0	0
CB	MDP	CB192	_1382	99	0	99	0	0	0	0
CB	MDP	CB192	_1383	209	2	207	0	0	0	0
CB	MDP	CB192	_1384	272	0	271	0	0	0	0
CB	MDP	CB192	_1385	72	0	71	0	0	0	0
CB	MDP	CB192	_1386	492	1	490	0	0	0	0

CB	MDP	CB192	_1387	19	0	19	0	0	0	0
CB	MDP	CB192	_1388	40	0	40	0	0	0	0
CB	MDP	CB192	_1389	94	0	2	92	0	0	0
CB	MDP	CB192	_1390	24	0	0	24	0	0	0
CB	MDP	CB192	_1391	131	0	0	131	0	0	0
CB	MDP	CB192	_1392	204	3	5	194	0	0	0
CB	MDP	CB192	_1393	29	0	1	28	0	0	0
CB	MDP	CB192	_1394	27	0	1	26	0	0	0
CB	MDP	CB192	_1395	62	0	0	62	0	0	0
CB	MDP	CB192	_1396	72	0	0	72	0	0	0
CB	MDP	CB192	_1397	88	0	0	0	88	0	0
CB	MDP	CB192	_1398	44	0	0	1	43	0	0
CB	GMDP	CB192	_1399	28	24	3	1	0	0	0
CB	GMDP	CB192	_1400	219	211	2	0	0	0	0
CB	GMDP	CB192	_1401	166	155	4	4	2	0	0
CB	GMDP	CB192	_1402	62	61	1	0	0	0	0
CB	GMDP	CB192	_1403	53	50	2	0	1	0	0
CB	GMDP	CB192	_1404	20	17	1	0	0	0	0
CB	GMDP	CB192	_1405	11	10	0	0	0	0	0
CB	GMDP	CB192	_1406	69	69	0	0	0	0	0
CB	GMDP	CB192	_1407	17	0	12	1	4	0	0
CB	GMDP	CB192	_1408	585	0	2	582	0	0	0
CB	GMDP	CB192	_1409	217	0	1	216	0	0	0
CB	GMDP	CB192	_1410	34	0	1	16	6	0	4
CB	GMDP	CB192	_1411	214	0	2	207	2	3	0
CB	GMDP	CB192	_1412	119	0	0	4	109	0	4
CB	BNKP	CB192	_1413	15	0	8	3	3	0	0
CB	BNKP	CB192	_1414	75	0	0	75	0	0	0
CB	BNKP	CB192	_1415	288	0	0	1	287	0	0
CB	BNKP	CB192	_1416	63	0	0	2	53	0	1
CB	BNKP	CB192	_1417	57	0	0	3	54	0	0
CB	BNKP	CB192	_1418	71	0	0	0	70	0	0
CB	BNKP	CB192	_1419	35	0	2	0	0	0	31
CB	BNKP	CB192	_1420	66	0	0	0	0	0	63
CB	BNKP	CB180	_1421	15	0	0	0	14	0	0
CB	BNKP	CB180	_1422	80	0	0	0	0	0	69
CB	BNKP	CB180	_1423	132	0	0	0	0	0	109
CB	BNKP	CB180	_1424	40	0	1	0	0	0	39
CB	BNKP	CB180	_1425	31	0	0	0	0	0	29
CB	BNKP	CB180	_1426	53	0	1	0	0	0	50
CB	BNKP	CB180	_1427	83	0	2	0	6	0	73
CB	BNKP	CB180	_1428	453	0	0	1	2	1	440
CB	BNKP	CB180	_1429	15	0	0	0	0	0	15
CB	BNKP	CB180	_1430	89	0	0	0	0	0	86
CB	BNKP	CB180	_1431	464	0	0	0	0	0	451
CB	BNKP	CB180	_1432	233	0	0	0	0	0	220
CB	BNKP	CB180	_1433	34	0	0	0	0	0	29
CB	BNKP	CB180	_1434	99	0	0	0	0	0	99
CB	BNKP	CB180	_1435	11	0	1	0	0	0	8
CB	BNKP	CB180	_1436	327	0	0	6	6	1	307
CB	BNKP	CB180	_1437	66	0	0	0	0	0	62
CB	BNKP	CB180	_1438	18	0	2	0	0	0	10
CB	BNKP	CB180	_1439	83	0	0	0	0	0	70
CB	BNKP	CB180	_1440	65	0	0	1	1	1	59

CB	BNKP	CB180	_1441	70	0	0	0	0	0	64
CB	BNKP	CB180	_1442	27	0	0	0	0	0	25
CB	BNKP	CB180	_1443	49	0	3	0	0	0	43
CB	BNKP	CB180	_1444	107	0	0	0	0	0	104
CB	BNKP	CB180	_1445	38	0	0	0	0	0	37
CB	BNKP	CB180	_1446	111	0	0	1	0	1	103
CB	BNKP	CB180	_1447	30	1	1	1	0	0	26
CB	BNKP	CB180	_1448	38	0	0	0	0	0	35
CB	BNKP	CB180	_1449	49	0	0	0	0	0	46
CB	BNKP	CB180	_1450	38	0	1	0	0	0	32
CB	BNKP	CB180	_1451	78	0	0	1	0	0	73
CB	BNKP	CB180	_1452	45	0	1	0	0	1	40
CB	BNKP	CB180	_1453	10	0	0	0	0	0	9
CB	BNKP	CB180	_1454	36	0	1	0	0	0	34
CB	BNKP	CB180	_1455	35	0	0	0	0	0	35
CB	BNKP	CB180	_1456	14	0	0	1	0	0	12
CB	BNKP	CB180	_1457	14	0	1	0	0	0	9
CB	MLP	CB179	_1458	30	0	0	26	0	2	1
CB	MLP	CB179	_1459	10	0	1	0	9	0	0
CB	MLP	CB179	_1460	161	0	0	1	158	0	2
CB	MLP	CB179	_1461	17	0	0	0	0	2	15
CB	MLP	CB179	_1462	1060	0	1	1	0	1	1046
CB	MLP	CB179	_1463	25	0	1	0	2	2	15
CB	MLP	CB179	_1464	298	0	3	0	0	3	292
CB	MLP	CB179	_1465	18	0	0	0	0	0	18
CB	MLP	CB179	_1466	12	0	1	0	2	0	9
CB	MLP	CB179	_1467	29	0	0	1	1	4	22
CB	MLP	CB179	_1468	650	0	0	1	0	1	595
CB	MLP	CB179	_1469	48	0	0	3	5	1	39
CB	MLP	CB179	_1470	25	0	1	3	0	3	17
CB	MLP	CB179	_1471	324	0	2	0	0	0	322
CB	MLP	CB179	_1472	126	0	0	3	0	2	121
CB	LMPP	CB179	_1473	70	43	4	0	0	0	0
CB	LMPP	CB179	_1474	568	553	3	0	0	1	1
CB	LMPP	CB179	_1475	17	8	1	0	0	0	2
CB	LMPP	CB179	_1476	87	0	4	3	79	0	0
CB	LMPP	CB179	_1477	156	0	3	1	0	0	102
CB	GMDP	CB179	_1478	516	516	0	0	0	0	0
CB	GMDP	CB179	_1479	135	124	5	5	0	0	0
CB	GMDP	CB179	_1480	89	81	6	0	0	1	0
CB	GMDP	CB179	_1481	34	32	2	0	0	0	0
CB	GMDP	CB179	_1482	284	280	0	1	3	0	0
CB	GMDP	CB179	_1483	90	86	1	1	2	0	0
CB	GMDP	CB179	_1484	336	336	0	0	0	0	0
CB	GMDP	CB179	_1485	120	117	1	0	2	0	0
CB	GMDP	CB179	_1486	169	168	0	1	0	0	0
CB	GMDP	CB179	_1487	78	76	1	0	0	0	0
CB	GMDP	CB179	_1488	263	263	0	0	0	0	0
CB	GMDP	CB179	_1489	114	109	5	0	0	0	0
CB	GMDP	CB179	_1490	138	137	1	0	0	0	0
CB	GMDP	CB179	_1491	86	83	2	1	0	0	0
CB	GMDP	CB179	_1492	11	0	11	0	0	0	0
CB	GMDP	CB179	_1493	727	0	726	0	0	0	0
CB	GMDP	CB179	_1494	96	3	93	0	0	0	0

CB	GMDP	CB179	_1495	20	0	15	1	0	1	0
CB	GMDP	CB179	_1496	13	0	9	0	3	1	0
CB	GMDP	CB179	_1497	12	0	8	0	3	1	0
CB	GMDP	CB179	_1498	16	6	7	0	0	2	0
CB	GMDP	CB179	_1499	215	0	212	0	0	0	0
CB	GMDP	CB179	_1500	27	0	4	19	0	2	0
CB	GMDP	CB179	_1501	296	1	2	5	287	0	0
CB	GMDP	CB179	_1502	27	0	3	2	20	2	0
CB	CMP	CB179	_1503	166	164	1	0	0	0	0
CB	CMP	CB179	_1504	41	34	2	0	0	0	0
CB	CMP	CB179	_1505	80	79	0	0	0	0	0
CB	CMP	CB179	_1506	182	179	1	0	0	0	0
CB	CMP	CB179	_1507	77	75	0	0	0	0	1
CB	CMP	CB179	_1508	83	81	1	1	0	0	0
CB	CMP	CB179	_1509	275	271	0	0	0	0	0
CB	CMP	CB179	_1510	200	197	2	0	0	0	0
CB	CMP	CB179	_1511	10	9	1	0	0	0	0
CB	CMP	CB179	_1512	126	122	0	2	2	0	0
CB	CMP	CB179	_1513	147	138	3	1	0	0	0
CB	CMP	CB179	_1514	35	27	2	2	2	2	0
CB	CMP	CB179	_1515	218	190	1	0	0	0	0
CB	CMP	CB179	_1516	14	0	7	3	0	1	0
CB	CMP	CB179	_1517	17	0	8	6	0	0	0
CB	CMP	CB179	_1518	57	1	6	32	0	0	1
CB	MLP	CB177	_1519	23	5	14	0	0	1	3
CB	MLP	CB177	_1520	22	0	0	0	0	1	13
CB	MLP	CB177	_1521	27	0	0	0	1	1	24
CB	MLP	CB177	_1522	21	0	0	0	0	0	21
CB	MLP	CB177	_1523	18	0	0	0	1	3	14
CB	MLP	CB177	_1524	212	0	0	1	1	4	202
CB	MLP	CB177	_1525	534	0	0	1	2	3	523
CB	LMPP	CB177	_1526	29	0	0	0	0	0	29
CB	LMPP	CB177	_1527	25	0	0	2	0	0	22
CB	LMPP	CB177	_1528	145	0	4	0	0	1	136
CB	LMPP	CB177	_1529	10	0	1	1	0	0	8
CB	LMPP	CB177	_1530	293	0	0	0	6	1	282
CB	LMPP	CB177	_1531	19	1	0	1	1	0	15
CB	LMPP	CB177	_1532	2301	0	2	1	3	4	2279
CB	LMPP	CB177	_1533	311	0	0	2	0	0	306
CB	LMPP	CB177	_1534	15	1	1	1	2	0	7
CB	LMPP	CB177	_1535	71	1	2	0	0	1	66
CB	LMPP	CB177	_1536	23	1	3	0	0	1	16
CB	LMPP	CB177	_1537	92	0	0	1	0	6	85
CB	LMPP	CB177	_1538	28	1	0	1	1	2	19
CB	LMPP	CB177	_1539	23	0	0	0	0	0	23
CB	GMDP	CB177	_1540	45	44	1	0	0	0	0
CB	GMDP	CB177	_1541	13	12	0	0	0	0	0
CB	GMDP	CB177	_1542	89	89	0	0	0	0	0
CB	GMDP	CB177	_1543	122	121	0	0	0	0	1
CB	GMDP	CB177	_1544	65	65	0	0	0	0	0
CB	GMDP	CB177	_1545	184	182	0	0	0	1	1
CB	GMDP	CB177	_1546	72	71	0	1	0	0	0
CB	GMDP	CB177	_1547	19	18	1	0	0	0	0
CB	GMDP	CB177	_1548	58	56	1	0	0	0	1

CB	GMDP	CB177	_1549	12	11	0	1	0	0	0
CB	GMDP	CB177	_1550	101	101	0	0	0	0	0
CB	GMDP	CB177	_1551	122	122	0	0	0	0	0
CB	GMDP	CB177	_1552	59	51	1	4	0	0	3
CB	GMDP	CB177	_1553	117	114	0	0	2	0	1
CB	GMDP	CB177	_1554	178	176	0	0	2	0	0
CB	GMDP	CB177	_1555	26	21	0	4	0	0	1
CB	GMDP	CB177	_1556	127	123	0	2	1	0	1
CB	GMDP	CB177	_1557	835	834	1	0	0	0	0
CB	GMDP	CB177	_1558	189	186	0	3	0	0	0
CB	GMDP	CB177	_1559	11	7	4	0	0	0	0
CB	GMDP	CB177	_1560	60	59	1	0	0	0	0
CB	GMDP	CB177	_1561	21	21	0	0	0	0	0
CB	GMDP	CB177	_1562	29	29	0	0	0	0	0
CB	GMDP	CB177	_1563	36	0	35	0	0	0	0
CB	GMDP	CB177	_1564	53	3	49	1	0	0	0
CB	GMDP	CB177	_1565	320	1	0	319	0	0	0
CB	GMDP	CB177	_1566	274	0	0	273	1	0	0
CB	GMDP	CB177	_1567	162	5	0	156	1	0	0
CB	GMDP	CB177	_1568	52	2	0	49	1	0	0
CB	GMDP	CB177	_1569	27	0	1	5	17	4	0
CB	GMDP	CB177	_1570	66	0	0	1	65	0	0
CB	GMDP	CB177	_1571	93	0	0	5	0	3	43
CB	GMDP	CB177	_1572	16	0	1	0	0	3	11
CB	CMP	CB177	_1573	16	16	0	0	0	0	0
CB	CMP	CB177	_1574	39	37	1	0	0	0	1
CB	CMP	CB177	_1575	175	174	0	0	0	0	0
CB	CMP	CB177	_1576	37	36	0	0	0	0	0
CB	CMP	CB177	_1577	516	504	1	0	0	0	0
CB	CMP	CB177	_1578	45	39	1	0	0	0	0
CB	CMP	CB177	_1579	17	15	0	0	0	0	1
CB	CMP	CB177	_1580	848	837	1	0	0	0	0
CB	CMP	CB177	_1581	62	46	1	0	0	0	0
CB	CMP	CB177	_1582	782	782	0	0	0	0	0
CB	CMP	CB177	_1583	121	117	3	0	0	0	0
CB	CMP	CB177	_1584	51	47	3	0	0	0	0
CB	CMP	CB177	_1585	35	14	3	0	0	0	0
CB	CMP	CB177	_1586	145	142	3	0	0	0	0
CB	CMP	CB177	_1587	396	4	361	0	0	0	0
CB	CMP	CB177	_1588	1005	2	28	1	0	1	6
CB	CMP	CB177	_1589	55	0	41	1	0	0	0
CB	CMP	CB177	_1590	15	0	10	0	0	0	0
CB	CMP	CB177	_1591	31	1	1	15	0	1	0
CB	CMP	CB177	_1592	759	0	0	0	0	0	50
CB	CMP	CB177	_1593	12	1	0	0	0	3	7
CB	CMP	CB177	_1594	34	1	0	0	0	3	22
CB	MPP	CB176	_1595	197	187	0	0	0	0	0
CB	MPP	CB176	_1596	1263	9	5	5	2	0	1
CB	MPP	CB176	_1597	624	10	0	0	0	0	0
CB	MPP	CB176	_1598	1186	1184	2	0	0	0	0
CB	MPP	CB176	_1599	989	792	0	0	0	0	0
CB	MPP	CB176	_1600	21	0	18	2	0	0	0
CB	MPP	CB176	_1601	22	4	17	0	1	0	0
CB	MPP	CB176	_1602	126	0	13	0	0	0	1

CB	MPP	CB176	_1603	13	1	10	1	0	0	0
CB	MPP	CB176	_1604	26	0	9	4	0	0	0
CB	MPP	CB176	_1605	15	1	9	1	0	0	0
CB	MPP	CB176	_1606	63	1	1	1	0	0	8
CB	MPP	CB176	_1607	866	4	6	1	0	1	15
CB	MPP	CB176	_1608	19	1	2	0	0	0	14
CB	LMPP	CB176	_1609	12	10	0	0	1	0	1
CB	LMPP	CB176	_1610	317	315	1	0	1	0	0
CB	LMPP	CB176	_1611	95	88	1	2	0	0	0
CB	LMPP	CB176	_1612	23	22	0	1	0	0	0
CB	LMPP	CB176	_1613	512	510	0	0	0	0	2
CB	LMPP	CB176	_1614	44	1	43	0	0	0	0
CB	LMPP	CB176	_1615	2623	1	2564	0	0	0	1
CB	LMPP	CB176	_1616	81	3	22	0	0	0	0
CB	LMPP	CB176	_1617	97	3	11	0	0	0	2
CB	LMPP	CB176	_1618	14	0	11	0	0	0	1
CB	LMPP	CB176	_1619	43	0	0	40	3	0	0
CB	LMPP	CB176	_1620	14	0	0	0	14	0	0
CB	LMPP	CB176	_1621	15	0	0	3	0	7	5
CB	LMPP	CB176	_1622	21	0	1	1	1	17	1
CB	LMPP	CB176	_1623	14	0	0	3	0	10	0
CB	LMPP	CB176	_1624	11	0	1	1	0	9	0
CB	LMPP	CB176	_1625	24	0	0	1	1	22	0
CB	LMPP	CB176	_1626	13	0	1	0	3	7	2
CB	LMPP	CB176	_1627	18	0	0	0	1	17	0
CB	LMPP	CB176	_1628	72	0	0	1	0	4	67
CB	LMPP	CB176	_1629	14	0	0	0	0	0	14
CB	LMPP	CB176	_1630	135	0	1	3	1	3	118
CB	LMPP	CB176	_1631	27	0	0	0	0	6	17
CB	LMPP	CB176	_1632	31	0	2	0	0	1	28
CB	LMPP	CB176	_1633	13	0	0	0	0	0	7
CB	LMPP	CB176	_1634	22	0	1	0	0	3	12
CB	LMPP	CB176	_1635	90	0	1	0	0	4	84
CB	HSC	CB176	_1636	636	299	1	0	0	0	0
CB	HSC	CB176	_1637	10	8	0	0	0	0	0
CB	HSC	CB176	_1638	46	34	0	0	0	0	0
CB	HSC	CB176	_1639	1188	503	1	0	0	0	0
CB	HSC	CB176	_1640	882	15	1	1	0	0	3
CB	HSC	CB176	_1641	1652	1649	0	0	0	0	0
CB	HSC	CB176	_1642	419	407	0	0	0	0	0
CB	HSC	CB176	_1643	45	0	33	5	0	0	1
CB	HSC	CB176	_1644	15	4	8	2	0	0	0
CB	HSC	CB176	_1645	35	0	24	3	1	0	1
CB	HSC	CB176	_1646	906	1	15	0	0	0	2
CB	HSC	CB176	_1647	16	0	5	1	0	0	9
CB	GMDP	CB176	_1648	15	12	1	2	0	0	0
CB	GMDP	CB176	_1649	50	49	0	1	0	0	0
CB	GMDP	CB176	_1650	141	141	0	0	0	0	0
CB	GMDP	CB176	_1651	22	21	0	1	0	0	0
CB	GMDP	CB176	_1652	41	41	0	0	0	0	0
CB	GMDP	CB176	_1653	29	28	0	0	0	0	1
CB	GMDP	CB176	_1654	212	212	0	0	0	0	0
CB	GMDP	CB176	_1655	17	17	0	0	0	0	0
CB	GMDP	CB176	_1656	15	14	1	0	0	0	0

CB	GMDP	CB176	_1657	255	254	0	0	0	1	0
CB	GMDP	CB176	_1658	151	143	6	0	0	0	0
CB	GMDP	CB176	_1659	141	141	0	0	0	0	0
CB	GMDP	CB176	_1660	75	75	0	0	0	0	0
CB	GMDP	CB176	_1661	41	36	2	3	0	0	0
CB	GMDP	CB176	_1662	47	44	3	0	0	0	0
CB	GMDP	CB176	_1663	67	67	0	0	0	0	0
CB	GMDP	CB176	_1664	163	163	0	0	0	0	0
CB	GMDP	CB176	_1665	108	108	0	0	0	0	0
CB	GMDP	CB176	_1666	22	21	1	0	0	0	0
CB	GMDP	CB176	_1667	368	367	0	0	0	0	0
CB	GMDP	CB176	_1668	27	26	0	1	0	0	0
CB	GMDP	CB176	_1669	47	44	1	0	0	0	0
CB	GMDP	CB176	_1670	238	236	0	0	0	0	0
CB	GMDP	CB176	_1671	27	26	1	0	0	0	0
CB	GMDP	CB176	_1672	420	418	1	0	0	0	1
CB	GMDP	CB176	_1673	32	30	0	0	0	0	0
CB	GMDP	CB176	_1674	22	22	0	0	0	0	0
CB	GMDP	CB176	_1675	95	94	0	0	0	0	0
CB	GMDP	CB176	_1676	17	16	0	0	0	0	0
CB	GMDP	CB176	_1677	39	36	0	0	0	0	0
CB	GMDP	CB176	_1678	17	16	0	0	0	0	0
CB	GMDP	CB176	_1679	16	14	0	0	0	0	0
CB	GMDP	CB176	_1680	11	2	9	0	0	0	0
CB	GMDP	CB176	_1681	10	0	10	0	0	0	0
CB	GMDP	CB176	_1682	31	3	28	0	0	0	0
CB	GMDP	CB176	_1683	14	1	11	1	0	0	0
CB	GMDP	CB176	_1684	23	3	19	1	0	0	0
CB	GMDP	CB176	_1685	27	0	0	1	18	0	2
CB	GMDP	CB176	_1686	22	1	1	6	14	0	0
CB	GMDP	CB176	_1687	60	0	0	2	54	3	1
CB	GMDP	CB176	_1688	16	0	0	0	0	12	1
CB	CMP	CB176	_1689	122	122	0	0	0	0	0
CB	CMP	CB176	_1690	111	107	1	2	0	0	0
CB	CMP	CB176	_1691	39	37	1	1	0	0	0
CB	CMP	CB176	_1692	545	543	2	0	0	0	0
CB	CMP	CB176	_1693	595	594	0	0	0	0	0
CB	CMP	CB176	_1694	1018	1016	0	0	0	0	0
CB	CMP	CB176	_1695	22	16	0	0	0	0	0
CB	CMP	CB176	_1696	44	19	0	0	0	0	0
CB	CMP	CB176	_1697	420	419	0	0	0	0	0
CB	CMP	CB176	_1698	205	204	0	1	0	0	0
CB	CMP	CB176	_1699	727	723	3	0	0	0	0
CB	CMP	CB176	_1700	91	89	0	0	0	0	0
CB	CMP	CB176	_1701	88	84	2	1	0	0	0
CB	CMP	CB176	_1702	90	89	0	0	0	0	0
CB	CMP	CB176	_1703	213	162	0	0	0	0	0
CB	CMP	CB176	_1704	148	73	2	0	0	0	0
CB	CMP	CB176	_1705	228	84	3	0	0	0	0
CB	CMP	CB176	_1706	297	13	0	0	0	0	0
CB	CMP	CB176	_1707	101	38	1	1	0	0	0
CB	CMP	CB176	_1708	120	8	2	0	0	0	0
CB	CMP	CB176	_1709	165	23	0	0	0	0	0
CB	CMP	CB176	_1710	33	3	27	0	0	0	0

CB	CMP	CB176	_1711	24	3	21	0	0	0	0
CB	CMP	CB176	_1712	72	1	66	0	0	0	0
CB	CMP	CB176	_1713	23	1	17	1	0	0	0
CB	CMP	CB176	_1714	300	0	0	29	6	0	5
CB	CMP	CB176	_1715	68	0	0	5	48	1	1
CB	CMP	CB176	_1716	305	0	2	0	0	0	56
CB	MLP	CB155	_1717	158	0	2	143	3	5	2
CB	MLP	CB155	_1718	17	0	0	9	1	5	2
CB	MLP	CB155	_1719	25	0	0	16	0	2	4
CB	MLP	CB155	_1720	88	0	0	4	84	0	0
CB	MLP	CB155	_1721	10	0	0	0	0	8	0
CB	LMPP	CB155	_1722	83	83	0	0	0	0	0
CB	LMPP	CB155	_1723	18	3	14	0	0	0	0
CB	LMPP	CB155	_1724	40	0	0	34	3	0	0
CB	LMPP	CB155	_1725	20	0	0	11	1	1	6
CB	LMPP	CB155	_1726	55	0	1	3	37	5	5
CB	LMPP	CB155	_1727	12	0	0	0	1	9	0
CB	LMPP	CB155	_1728	40	0	0	6	5	11	4
CB	LMPP	CB155	_1729	29	0	0	3	0	4	8
CB	LMPP	CB155	_1730	94	0	0	2	0	0	69
CB	LMPP	CB155	_1731	12	1	0	0	0	0	9
CB	LMPP	CB155	_1732	157	0	0	5	0	5	40
CB	HSC	CB155	_1733	2127	1	16	1	0	1	1
CB	CMP	CB155	_1734	575	568	0	0	0	0	0
CB	CMP	CB155	_1735	65	63	0	1	0	0	1
CB	CMP	CB155	_1736	800	800	0	0	0	0	0
CB	CMP	CB155	_1737	322	322	0	0	0	0	0
CB	CMP	CB155	_1738	617	616	0	0	0	0	0
CB	CMP	CB155	_1739	61	61	0	0	0	0	0
CB	MLP	CB154	_1740	23	0	0	0	0	9	4
CB	MLP	CB154	_1741	25	0	0	1	0	0	7
CB	LMPP	CB154	_1742	19	0	4	13	0	0	0
CB	LMPP	CB154	_1743	40	0	0	2	8	0	6
CB	LMPP	CB154	_1744	10	0	0	0	10	0	0
CB	LMPP	CB154	_1745	120	0	0	0	115	3	1
CB	LMPP	CB154	_1746	99	0	0	0	0	0	11
CB	CMP	CB154	_1747	32	5	27	0	0	0	0
CB	CMP	CB154	_1748	225	0	0	5	2	18	6
CB	MPP	CB114	_1749	36	0	19	0	4	0	0
CB	MPP	CB114	_1750	22	1	9	0	2	0	0
CB	MPP	CB114	_1751	43	0	0	4	36	0	1
CB	MPP	CB114	_1752	21	0	1	2	8	0	0
CB	MPP	CB114	_1753	18	1	1	1	7	0	3
CB	MPP	CB114	_1754	23	0	0	0	19	0	1
CB	MPP	CB114	_1755	1336	0	2	0	2	0	60
CB	MPP	CB114	_1756	99	3	0	0	0	1	11
CB	MLP	CB114	_1757	12	1	10	0	0	0	0
CB	MLP	CB114	_1758	34	0	2	17	1	5	5
CB	MLP	CB114	_1759	53	0	2	0	0	0	51
CB	HSC	CB114	_1760	42	5	11	4	2	0	4
CB	HSC	CB114	_1761	31	0	8	0	0	0	0
CB	HSC	CB114	_1762	16	1	0	0	8	0	0
CB	HSC	CB114	_1763	37	0	2	0	0	0	10
CB	HSC	CB114	_1764	2433	0	4	0	0	0	7

CB	GMDP	CB114	_1765	28	28	0	0	0	0	0
CB	GMDP	CB114	_1766	77	72	5	0	0	0	0
CB	GMDP	CB114	_1767	115	114	0	0	0	0	0
CB	GMDP	CB114	_1768	14	12	0	0	1	0	0
CB	GMDP	CB114	_1769	25	24	0	0	0	0	0
CB	GMDP	CB114	_1770	10	9	1	0	0	0	0
CB	GMDP	CB114	_1771	334	1	329	0	0	0	0
CB	GMDP	CB114	_1772	77	0	0	75	0	0	0
CB	GMDP	CB114	_1773	94	0	0	3	89	0	0
CB	CMP	CB114	_1774	560	532	2	0	0	0	0
CB	CMP	CB114	_1775	51	50	0	0	0	0	0
CB	BNKP	CB114	_1776	16	0	2	1	9	1	1
CB	BNKP	CB114	_1777	11	0	1	1	9	0	0
CB	BNKP	CB114	_1778	146	0	0	0	145	1	0
CB	BNKP	CB114	_1779	32	0	0	0	0	0	31
CB	BNKP	CB114	_1780	17	0	1	0	0	0	15
CB	BNKP	CB114	_1781	248	0	0	0	0	1	244
CB	BNKP	CB114	_1782	115	0	0	0	0	1	114
CB	BNKP	CB114	_1783	120	0	1	0	0	0	114
CB	MPP	CB110	_1784	4207	4168	5	0	1	4	0
CB	MPP	CB110	_1785	183	167	2	0	0	1	2
CB	MPP	CB110	_1786	16	0	12	0	0	0	2
CB	MPP	CB110	_1787	27	1	4	5	12	0	2
CB	MPP	CB110	_1788	47	4	0	0	0	0	41
CB	MPP	CB110	_1789	86	3	3	5	3	3	36
CB	MPP	CB110	_1790	210	0	1	1	0	1	16
CB	MPP	CB110	_1791	18	0	1	2	0	0	12
CB	MPP	CB110	_1792	22	0	1	0	3	0	16
CB	MPP	CB110	_1793	18	0	0	1	1	0	9
CB	MLP	CB110	_1794	13	0	1	8	0	4	0
CB	MLP	CB110	_1795	24	0	0	1	21	0	1
CB	MLP	CB110	_1796	23	0	1	0	16	6	0
CB	MLP	CB110	_1797	18	0	2	0	0	7	5
CB	MLP	CB110	_1798	13	0	0	0	4	9	0
CB	MLP	CB110	_1799	31	0	0	0	1	0	30
CB	LMPP	CB110	_1800	15	0	1	1	8	1	0
CB	LMPP	CB110	_1801	30	0	0	2	26	0	0
CB	LMPP	CB110	_1802	21	0	3	0	9	2	5
CB	LMPP	CB110	_1803	12	0	1	1	1	8	1
CB	LMPP	CB110	_1804	1234	2	2	5	5	5	1061
CB	HSC	CB110	_1805	100	98	0	0	0	0	0
CB	HSC	CB110	_1806	36	0	5	13	0	1	0
CB	HSC	CB110	_1807	36	0	1	4	1	6	19
CB	HSC	CB110	_1808	41	1	0	0	4	1	26
CB	HSC	CB110	_1809	58	5	3	2	0	5	17
CB	GMDP	CB110	_1810	67	62	4	0	0	0	0
CB	GMDP	CB110	_1811	36	31	2	0	3	0	0
CB	GMDP	CB110	_1812	13	11	0	0	0	0	0
CB	GMDP	CB110	_1813	91	89	1	0	0	0	0
CB	GMDP	CB110	_1814	21	19	1	0	1	0	0
CB	GMDP	CB110	_1815	280	2	278	0	0	0	0
CB	GMDP	CB110	_1816	61	2	59	0	0	0	0
CB	GMDP	CB110	_1817	381	1	378	0	1	0	0
CB	GMDP	CB110	_1818	271	1	268	1	0	0	0

CB	GMDP	CB110	_1819	45	0	44	1	0	0	0
CB	GMDP	CB110	_1820	114	0	3	106	4	0	0
CB	GMDP	CB110	_1821	47	0	6	40	1	0	0
CB	GMDP	CB110	_1822	323	0	2	5	313	0	0
CB	GMDP	CB110	_1823	228	0	3	1	224	0	0
CB	CMP	CB110	_1824	39	37	0	0	0	0	0
CB	CMP	CB110	_1825	269	267	2	0	0	0	0
CB	CMP	CB110	_1826	10	10	0	0	0	0	0
CB	CMP	CB110	_1827	362	352	2	0	0	0	0
CB	CMP	CB110	_1828	498	0	495	0	0	0	0
CB	CMP	CB110	_1829	12	0	12	0	0	0	0
CB	CMP	CB110	_1830	14	0	14	0	0	0	0
CB	BNKP	CB110	_1831	58	0	0	2	56	0	0
CB	BNKP	CB110	_1832	77	0	0	3	74	0	0
CB	MPP	CB109	_1833	107	54	1	0	0	0	0
CB	MPP	CB109	_1834	158	7	3	0	0	0	0
CB	MPP	CB109	_1835	32	4	21	4	1	0	0
CB	MPP	CB109	_1836	20	0	11	1	4	0	0
CB	MPP	CB109	_1837	19	1	1	4	11	0	0
CB	MPP	CB109	_1838	74	1	2	5	64	0	1
CB	MPP	CB109	_1839	21	0	1	0	1	7	4
CB	MPP	CB109	_1840	24	1	0	0	3	2	16
CB	MLP	CB109	_1841	19	0	2	13	3	0	1
CB	MLP	CB109	_1842	22	0	0	0	22	0	0
CB	MLP	CB109	_1843	16	0	1	0	2	12	1
CB	LMPP	CB109	_1844	11	0	1	0	1	0	8
CB	HSC	CB109	_1845	35	23	3	0	3	1	1
CB	HSC	CB109	_1846	828	147	0	0	0	6	0
CB	GMDP	CB109	_1847	22	21	0	0	0	0	0
CB	GMDP	CB109	_1848	16	8	5	0	0	0	0
CB	GMDP	CB109	_1849	13	0	11	0	0	0	2
CB	GMDP	CB109	_1850	44	0	0	4	38	1	1
CB	GMDP	CB109	_1851	121	0	0	0	120	1	0
CB	GMDP	CB109	_1852	207	0	1	5	200	0	0
CB	CMP	CB109	_1853	200	188	1	3	4	0	0
CB	CMP	CB109	_1854	76	75	1	0	0	0	0
CB	CMP	CB109	_1855	143	140	1	0	0	0	0
CB	CMP	CB109	_1856	11	0	10	1	0	0	0
CB	BNKP	CB109	_1857	16	0	1	0	11	0	4
CB	BNKP	CB109	_1858	47	0	1	0	46	0	0
CB	BNKP	CB109	_1859	22	0	0	1	19	2	0
CB	BNKP	CB109	_1860	12	0	1	0	11	0	0
CB	BNKP	CB109	_1861	17	0	2	1	13	0	1
CB	BNKP	CB109	_1862	27	0	1	0	0	0	26
CB	BNKP	CB109	_1863	17	0	3	0	0	0	14
CB	MPP	CB106	_1864	5324	0	9	2	0	2	2
CB	MPP	CB106	_1865	211	0	6	20	0	0	1
CB	MPP	CB106	_1866	143	0	1	14	0	2	2
CB	MPP	CB106	_1867	775	0	4	20	0	4	0
CB	MPP	CB106	_1868	169	0	5	113	0	2	0
CB	MPP	CB106	_1869	1520	0	2	1	0	2	11
CB	MPP	CB106	_1870	2116	0	5	0	0	1	11
CB	MLP	CB106	_1871	3286	6	3008	5	0	1	1
CB	MLP	CB106	_1872	18	0	0	1	0	16	1

CB	MLP	CB106	_1873	73	0	0	0	0	0	10
CB	MLP	CB106	_1874	453	0	2	0	0	0	421
CB	MLP	CB106	_1875	76	0	0	1	0	3	64
CB	MLP	CB106	_1876	928	0	0	0	0	1	903
CB	MLP	CB106	_1877	704	0	1	0	0	0	693
CB	MLP	CB106	_1878	1054	0	1	0	0	2	1024
CB	MLP	CB106	_1879	23	0	1	1	0	0	20
CB	MLP	CB106	_1880	197	0	0	0	0	0	179
CB	MLP	CB106	_1881	421	0	2	0	0	0	339
CB	MLP	CB106	_1882	11	0	0	0	0	0	9
CB	MLP	CB106	_1883	639	0	2	0	0	0	555
CB	MLP	CB106	_1884	711	0	1	1	0	1	696
CB	MLP	CB106	_1885	876	0	3	0	0	0	775
CB	MLP	CB106	_1886	959	0	1	2	0	3	896
CB	MLP	CB106	_1887	228	0	0	1	0	1	223
CB	MLP	CB106	_1888	623	0	2	1	0	1	460
CB	MLP	CB106	_1889	126	0	1	1	0	0	107
CB	MLP	CB106	_1890	163	0	0	0	0	0	158
CB	LMPP	CB106	_1891	24	6	15	1	0	0	1
CB	LMPP	CB106	_1892	1977	3	521	0	0	1	0
CB	LMPP	CB106	_1893	64	0	0	11	0	0	0
CB	LMPP	CB106	_1894	859	0	1	681	0	4	1
CB	LMPP	CB106	_1895	66	0	0	56	0	3	1
CB	LMPP	CB106	_1896	19	1	3	8	0	0	5
CB	LMPP	CB106	_1897	17	0	1	15	0	0	0
CB	LMPP	CB106	_1898	12	0	0	3	0	8	0
CB	LMPP	CB106	_1899	78	1	2	1	0	2	71
CB	LMPP	CB106	_1900	220	1	1	1	0	0	215
CB	LMPP	CB106	_1901	757	0	1	2	0	1	446
CB	LMPP	CB106	_1902	175	0	2	2	0	0	161
CB	LMPP	CB106	_1903	252	0	0	6	0	1	212
CB	LMPP	CB106	_1904	356	0	1	0	0	0	294
CB	LMPP	CB106	_1905	161	0	1	1	0	0	84
CB	LMPP	CB106	_1906	560	0	0	3	0	4	386
CB	LMPP	CB106	_1907	68	0	1	2	0	1	17
CB	HSC	CB106	_1908	2484	0	5	30	0	4	2
CB	HSC	CB106	_1909	271	0	3	54	0	1	2
CB	HSC	CB106	_1910	60	0	3	29	0	0	1
CB	HSC	CB106	_1911	2119	0	2	1	0	0	8
CB	GMDP	CB106	_1912	361	361	0	0	0	0	0
CB	GMDP	CB106	_1913	18	12	5	0	0	0	1
CB	GMDP	CB106	_1914	17	16	0	0	0	0	0
CB	GMDP	CB106	_1915	83	81	1	0	1	0	0
CB	GMDP	CB106	_1916	412	409	2	0	0	0	1
CB	GMDP	CB106	_1917	44	42	1	0	0	0	1
CB	GMDP	CB106	_1918	17	16	1	0	0	0	0
CB	GMDP	CB106	_1919	23	23	0	0	0	0	0
CB	GMDP	CB106	_1920	116	116	0	0	0	0	0
CB	GMDP	CB106	_1921	18	18	0	0	0	0	0
CB	GMDP	CB106	_1922	48	46	1	0	0	0	0
CB	GMDP	CB106	_1923	20	20	0	0	0	0	0
CB	GMDP	CB106	_1924	61	60	0	1	0	0	0
CB	GMDP	CB106	_1925	44	44	0	0	0	0	0
CB	GMDP	CB106	_1926	38	32	3	2	0	0	0

CB	GMDP	CB106	_1927	86	83	0	2	0	0	0
CB	GMDP	CB106	_1928	69	65	2	2	0	0	0
CB	GMDP	CB106	_1929	28	3	22	0	1	0	0
CB	GMDP	CB106	_1930	53	2	50	0	0	0	0
CB	GMDP	CB106	_1931	110	6	103	1	0	0	0
CB	GMDP	CB106	_1932	20	2	16	0	1	0	0
CB	GMDP	CB106	_1933	32	6	9	0	0	4	1
CB	GMDP	CB106	_1934	457	4	450	3	0	0	0
CB	GMDP	CB106	_1935	655	2	652	0	0	0	0
CB	GMDP	CB106	_1936	639	2	636	0	0	0	0
CB	GMDP	CB106	_1937	47	4	42	1	0	0	0
CB	GMDP	CB106	_1938	163	6	150	6	0	0	0
CB	GMDP	CB106	_1939	15	5	1	7	2	0	0
CB	GMDP	CB106	_1940	13	0	3	9	0	0	0
CB	GMDP	CB106	_1941	27	0	4	0	20	0	1
CB	GMDP	CB106	_1942	43	5	5	0	0	7	1
CB	CMP	CB106	_1943	21	13	2	1	0	0	0
CB	CMP	CB106	_1944	10	9	0	0	0	0	0
CB	CMP	CB106	_1945	24	19	5	0	0	0	0
CB	CMP	CB106	_1946	11	10	1	0	0	0	0
CB	CMP	CB106	_1947	16	16	0	0	0	0	0
CB	CMP	CB106	_1948	31	0	9	3	0	0	0
CB	CMP	CB106	_1949	54	5	38	0	0	0	2
CB	CMP	CB106	_1950	62	0	24	0	0	0	1
CB	CMP	CB106	_1951	12	1	2	9	0	0	0
CB	BNKP	CB106	_1952	56	0	0	39	0	0	1
CB	BNKP	CB106	_1953	32	0	0	1	31	0	0
CB	BNKP	CB106	_1954	63	0	0	0	63	0	0
CB	BNKP	CB106	_1955	224	0	0	0	0	0	221
CB	BNKP	CB106	_1956	165	0	0	0	0	0	164
CB	BNKP	CB106	_1957	299	0	0	0	0	0	296
CB	BNKP	CB106	_1958	1529	0	1	0	0	0	1150
CB	BNKP	CB106	_1959	670	0	1	0	0	0	516
CB	BNKP	CB106	_1960	17	0	0	0	0	0	16
CB	BNKP	CB106	_1961	467	0	0	0	0	0	368
CB	BNKP	CB106	_1962	231	0	0	0	0	0	210
CB	BNKP	CB106	_1963	293	0	0	0	0	0	256
CB	BNKP	CB106	_1964	1708	0	0	0	0	0	1088
CB	BNKP	CB106	_1965	148	0	0	0	0	0	148
CB	BNKP	CB106	_1966	860	0	4	0	0	0	775
CB	BNKP	CB106	_1967	236	0	2	0	0	0	227
CB	BNKP	CB106	_1968	218	1	0	0	0	0	216
CB	BNKP	CB106	_1969	11	0	0	0	0	0	11
CB	BNKP	CB106	_1970	15	0	0	0	0	0	14
CB	BNKP	CB106	_1971	10	0	0	0	0	0	10
CB	BNKP	CB106	_1972	429	0	3	0	0	0	407
CB	BNKP	CB106	_1973	222	0	0	0	0	0	215
CB	BNKP	CB106	_1974	48	0	0	0	0	0	48
CB	BNKP	CB106	_1975	382	0	0	1	0	3	335
CB	BNKP	CB106	_1976	255	0	0	0	0	0	237
CB	BNKP	CB106	_1977	811	0	4	1	0	0	757
CB	BNKP	CB106	_1978	307	0	0	0	0	0	209
CB	BNKP	CB106	_1979	366	0	1	0	0	0	362
CB	BNKP	CB106	_1980	366	0	0	0	0	0	346

CB	BNKP	CB106	_1981	372	0	0	0	0	0	370
CB	BNKP	CB106	_1982	878	0	1	0	0	0	825
CB	MLP	CB100	_1983	496	0	464	1	0	0	5
CB	MLP	CB100	_1984	17	0	1	0	1	0	10
CB	MLP	CB100	_1985	31	0	2	0	0	4	23
CB	MLP	CB100	_1986	50	0	0	0	0	0	48
CB	MLP	CB100	_1987	52	0	1	0	0	0	48
CB	GMDP	CB100	_1988	10	9	0	0	0	0	0
CB	GMDP	CB100	_1989	42	40	2	0	0	0	0
CB	GMDP	CB100	_1990	18	18	0	0	0	0	0
CB	GMDP	CB100	_1991	29	27	0	0	0	0	0
CB	GMDP	CB100	_1992	23	23	0	0	0	0	0
CB	GMDP	CB100	_1993	232	1	231	0	0	0	0
CB	GMDP	CB100	_1994	15	2	13	0	0	0	0
CB	CMP	CB100	_1995	11	11	0	0	0	0	0
CB	CMP	CB100	_1996	55	53	0	0	0	0	0
CB	CMP	CB100	_1997	292	289	2	0	0	0	0
CB	CMP	CB100	_1998	66	60	2	0	0	0	0
CB	BNKP	CB100	_1999	20	0	0	0	0	0	20
CB	CDP	CB087	_2000	7	1	4	0	0	0	0
CB	CDP	CB087	_2001	5	0	4	1	0	0	0
CB	CDP	CB087	_2002	11	0	0	11	0	0	0
CB	CDP	CB087	_2003	14	0	1	13	0	0	0
CB	CDP	CB087	_2004	14	0	1	12	0	0	0
CB	CDP	CB087	_2005	4	0	1	3	0	0	0
CB	CDP	CB087	_2006	5	0	0	5	0	0	0
CB	CDP	CB087	_2007	4	0	0	3	0	0	0
CB	CDP	CB087	_2008	4	0	0	3	0	0	0
CB	CDP	CB087	_2009	9	1	0	8	0	0	0
CB	CDP	CB087	_2010	3	0	0	3	0	0	0
CB	CDP	CB087	_2011	7	0	1	0	0	4	1
CB	CDP	CB087	_2012	3	0	0	0	0	3	0
CB	CDP	CB087	_2013	3	0	0	0	0	3	0
CB	CDP	CB087	_2014	4	0	0	0	0	3	1
CB	MDP	CB065	_2015	90	1	89	0	0	0	0
CB	MDP	CB065	_2016	1125	0	1125	0	0	0	0
CB	MDP	CB065	_2017	434	0	434	0	0	0	0
CB	MDP	CB065	_2018	236	0	236	0	0	0	0
CB	MDP	CB065	_2019	384	0	384	0	0	0	0
CB	MDP	CB065	_2020	645	1	644	0	0	0	0
CB	MDP	CB065	_2021	41	0	40	1	0	0	0
CB	MDP	CB065	_2022	231	1	230	0	0	0	0
CB	MDP	CB065	_2023	119	4	115	0	0	0	0
CB	MDP	CB065	_2024	1098	4	1093	1	0	0	0
CB	MDP	CB065	_2025	264	4	260	0	0	0	0
CB	MDP	CB065	_2026	957	4	952	0	1	0	0
CB	MDP	CB065	_2027	92	0	3	89	0	0	0
CB	MDP	CB065	_2028	64	1	4	59	0	0	0
CB	MDP	CB065	_2029	120	0	0	2	118	0	0
CB	GMDP	CB065	_2030	19	14	0	4	1	0	0
CB	GMDP	CB065	_2031	21	16	4	0	0	0	1
CB	GMDP	CB065	_2032	30	30	0	0	0	0	0
CB	GMDP	CB065	_2033	18	0	10	6	0	0	0
CB	GMDP	CB065	_2034	27	0	26	0	0	0	0

CB	GMDP	CB065	_2035	33	0	0	32	0	0	0
CB	GMDP	CB065	_2036	46	1	1	6	0	0	28
CB	GMDP	CB065	_2037	943	0	0	0	0	0	75
CB	CDP	CB065	_2038	14	0	0	14	0	0	0
CB	CDP	CB065	_2039	12	0	0	12	0	0	0
CB	CDP	CB065	_2040	10	0	0	10	0	0	0
CB	CDP	CB065	_2041	15	0	0	15	0	0	0
CB	CDP	CB065	_2042	11	0	0	11	0	0	0
CB	CDP	CB065	_2043	26	0	0	24	0	1	0
CB	CDP	CB065	_2044	32	0	0	32	0	0	0
CB	CDP	CB065	_2045	48	0	0	48	0	0	0
CB	CDP	CB065	_2046	10	0	0	10	0	0	0
CB	CDP	CB065	_2047	21	0	0	20	1	0	0
CB	CDP	CB065	_2048	21	0	0	20	1	0	0
CB	CDP	CB065	_2049	23	0	0	23	0	0	0
CB	CDP	CB065	_2050	22	0	0	22	0	0	0
CB	CDP	CB065	_2051	43	0	0	43	0	0	0
CB	CDP	CB065	_2052	15	0	0	15	0	0	0
CB	CDP	CB065	_2053	12	0	0	12	0	0	0
CB	CDP	CB065	_2054	17	0	0	17	0	0	0
CB	CDP	CB065	_2055	33	0	0	33	0	0	0
CB	CDP	CB065	_2056	13	0	0	13	0	0	0
CB	CDP	CB065	_2057	11	0	0	11	0	0	0
CB	CDP	CB065	_2058	18	0	0	17	0	1	0
CB	CDP	CB065	_2059	17	0	0	17	0	0	0
CB	CDP	CB065	_2060	41	0	0	41	0	0	0
CB	CDP	CB065	_2061	34	0	0	34	0	0	0
CB	CDP	CB065	_2062	22	0	0	22	0	0	0
CB	CDP	CB065	_2063	18	0	0	18	0	0	0
CB	CDP	CB065	_2064	23	0	0	23	0	0	0
CB	CDP	CB065	_2065	29	0	0	29	0	0	0
CB	CDP	CB065	_2066	13	0	0	13	0	0	0
CB	CDP	CB065	_2067	33	0	0	33	0	0	0
CB	CDP	CB065	_2068	18	0	0	18	0	0	0
CB	CDP	CB065	_2069	22	0	0	22	0	0	0
CB	CDP	CB065	_2070	20	0	0	20	0	0	0
CB	CDP	CB065	_2071	10	0	0	9	1	0	0
CB	CDP	CB065	_2072	17	0	0	16	1	0	0
CB	CDP	CB065	_2073	22	0	0	22	0	0	0
CB	CDP	CB065	_2074	8	0	0	8	0	0	0
CB	CDP	CB065	_2075	8	0	0	8	0	0	0
CB	CDP	CB065	_2076	8	0	0	8	0	0	0
CB	CDP	CB065	_2077	6	0	0	6	0	0	0
CB	CDP	CB065	_2078	7	0	0	7	0	0	0
CB	CDP	CB065	_2079	8	0	0	8	0	0	0
CB	CDP	CB065	_2080	3	0	0	3	0	0	0
CB	CDP	CB065	_2081	3	0	0	3	0	0	0
CB	CDP	CB065	_2082	4	0	1	3	0	0	0
CB	CDP	CB065	_2083	7	0	0	6	1	0	0
CB	CDP	CB065	_2084	5	0	0	5	0	0	0
CB	CDP	CB065	_2085	6	0	0	6	0	0	0
CB	CDP	CB065	_2086	5	0	0	5	0	0	0
CB	CDP	CB065	_2087	5	0	0	5	0	0	0
CB	CDP	CB065	_2088	8	0	0	8	0	0	0

CB	CDP	CB065	_2089	6	0	0	6	0	0	0
CB	CDP	CB065	_2090	8	0	0	8	0	0	0
CB	CDP	CB065	_2091	5	0	0	5	0	0	0
CB	CDP	CB065	_2092	8	0	0	8	0	0	0
CB	CDP	CB065	_2093	7	0	0	7	0	0	0
CB	CDP	CB065	_2094	7	0	0	7	0	0	0
CB	CDP	CB065	_2095	5	1	0	4	0	0	0
CB	CDP	CB065	_2096	7	0	0	7	0	0	0
CB	CDP	CB065	_2097	7	0	0	7	0	0	0
CB	CDP	CB065	_2098	5	0	0	5	0	0	0
CB	CDP	CB065	_2099	4	0	0	4	0	0	0
CB	CDP	CB065	_2100	8	0	0	8	0	0	0
CB	CDP	CB065	_2101	9	0	0	9	0	0	0
CB	CDP	CB065	_2102	5	0	0	4	1	0	0
CB	CDP	CB065	_2103	5	0	0	5	0	0	0
CB	CDP	CB065	_2104	14	0	0	0	14	0	0
CB	CDP	CB065	_2105	52	0	0	0	52	0	0
CB	CDP	CB065	_2106	57	0	0	0	57	0	0
CB	CDP	CB065	_2107	52	1	0	1	50	0	0
CB	CDP	CB065	_2108	41	0	0	0	40	0	0
CB	CDP	CB065	_2109	12	1	0	0	11	0	0
CB	CDP	CB065	_2110	20	0	0	0	20	0	0
CB	CDP	CB065	_2111	53	0	0	0	53	0	0
CB	CDP	CB065	_2112	16	0	0	0	16	0	0
CB	CDP	CB065	_2113	4	0	0	0	4	0	0
CB	CDP	CB065	_2114	3	0	0	0	0	3	0
CB	CDP	CB065	_2115	4	0	0	0	0	4	0
CB	MDP	CB061	_2116	10	9	1	0	0	0	0
CB	MDP	CB061	_2117	154	1	153	0	0	0	0
CB	MDP	CB061	_2118	62	1	61	0	0	0	0
CB	MDP	CB061	_2119	90	1	59	0	0	0	0
CB	MDP	CB061	_2120	313	0	313	0	0	0	0
CB	MDP	CB061	_2121	656	2	506	1	0	0	0
CB	MDP	CB061	_2122	421	1	419	0	0	0	1
CB	MDP	CB061	_2123	92	0	92	0	0	0	0
CB	MDP	CB061	_2124	284	2	278	4	0	0	0
CB	MDP	CB061	_2125	31	1	30	0	0	0	0
CB	MDP	CB061	_2126	173	1	172	0	0	0	0
CB	MDP	CB061	_2127	252	1	251	0	0	0	0
CB	MDP	CB061	_2128	119	2	117	0	0	0	0
CB	MDP	CB061	_2129	97	1	96	0	0	0	0
CB	MDP	CB061	_2130	76	1	75	0	0	0	0
CB	MDP	CB061	_2131	284	2	281	1	0	0	0
CB	MDP	CB061	_2132	229	1	228	0	0	0	0
CB	MDP	CB061	_2133	50	6	42	1	0	0	0
CB	MDP	CB061	_2134	548	2	546	0	0	0	0
CB	MDP	CB061	_2135	548	0	546	2	0	0	0
CB	MDP	CB061	_2136	70	0	70	0	0	0	0
CB	MDP	CB061	_2137	56	0	54	2	0	0	0
CB	MDP	CB061	_2138	56	0	56	0	0	0	0
CB	MDP	CB061	_2139	101	0	100	0	0	0	0
CB	MDP	CB061	_2140	27	0	22	5	0	0	0
CB	MDP	CB061	_2141	446	1	434	0	0	0	0
CB	MDP	CB061	_2142	281	0	279	2	0	0	0

CB	MDP	CB061	_2143	76	0	76	0	0	0	0
CB	MDP	CB061	_2144	72	0	70	0	0	0	0
CB	MDP	CB061	_2145	979	1	0	978	0	0	0
CB	MDP	CB061	_2146	16	0	0	14	0	2	0
CB	MDP	CB061	_2147	640	0	2	638	0	0	0
CB	MDP	CB061	_2148	1492	3	1	1488	0	0	0
CB	MDP	CB061	_2149	1390	0	2	1388	0	0	0
CB	MDP	CB061	_2150	264	0	1	263	0	0	0
CB	MDP	CB061	_2151	922	0	6	916	0	0	0
CB	MDP	CB061	_2152	871	1	1	869	0	0	0
CB	MDP	CB061	_2153	288	0	0	288	0	0	0
CB	MDP	CB061	_2154	691	1	0	690	0	0	0
CB	MDP	CB061	_2155	893	0	0	893	0	0	0
CB	MDP	CB061	_2156	1207	0	1	1206	0	0	0
CB	MDP	CB061	_2157	161	0	0	161	0	0	0
CB	MDP	CB061	_2158	1027	1	3	1023	0	0	0
CB	MDP	CB061	_2159	982	0	2	980	0	0	0
CB	MDP	CB061	_2160	136	0	1	135	0	0	0
CB	MDP	CB061	_2161	1048	0	1	1047	0	0	0
CB	MDP	CB061	_2162	860	0	0	860	0	0	0
CB	MDP	CB061	_2163	606	1	0	605	0	0	0
CB	MDP	CB061	_2164	513	1	4	508	0	0	0
CB	MDP	CB061	_2165	26	0	0	26	0	0	0
CB	MDP	CB061	_2166	42	0	0	42	0	0	0
CB	MDP	CB061	_2167	47	0	0	2	45	0	0
CB	MDP	CB061	_2168	34	0	0	4	30	0	0
CB	MDP	CB061	_2169	227	0	0	1	226	0	0
CB	MDP	CB061	_2170	18	0	0	1	0	17	0
CB	MDP	CB061	_2171	11	0	1	0	0	0	10
CB	GMDP	CB061	_2172	476	367	4	0	0	0	0
CB	GMDP	CB061	_2173	202	178	2	0	0	0	0
CB	GMDP	CB061	_2174	263	31	5	6	0	1	0
CB	GMDP	CB061	_2175	24	16	1	0	0	0	0
CB	GMDP	CB061	_2176	42	42	0	0	0	0	0
CB	GMDP	CB061	_2177	128	116	0	0	0	0	1
CB	GMDP	CB061	_2178	85	82	1	1	0	0	0
CB	GMDP	CB061	_2179	410	404	0	6	0	0	0
CB	GMDP	CB061	_2180	91	51	1	0	0	0	0
CB	GMDP	CB061	_2181	136	136	0	0	0	0	0
CB	GMDP	CB061	_2182	121	121	0	0	0	0	0
CB	GMDP	CB061	_2183	76	76	0	0	0	0	0
CB	GMDP	CB061	_2184	46	8	1	4	0	0	0
CB	GMDP	CB061	_2185	89	88	0	1	0	0	0
CB	GMDP	CB061	_2186	296	25	2	0	0	0	0
CB	GMDP	CB061	_2187	40	13	1	0	0	0	0
CB	GMDP	CB061	_2188	486	0	485	0	0	0	0
CB	GMDP	CB061	_2189	23	1	0	22	0	0	0
CB	GMDP	CB061	_2190	21	0	0	18	0	3	0
CB	GMDP	CB061	_2191	29	0	0	21	5	0	0
CB	GMDP	CB061	_2192	2590	4	0	2415	0	1	0
CB	GMDP	CB061	_2193	457	1	1	442	6	2	0
CB	GMDP	CB061	_2194	12	3	0	8	0	0	0
CB	GMDP	CB061	_2195	11	0	0	8	0	0	2
CB	GMDP	CB061	_2196	45	0	0	45	0	0	0

CB	GMDP	CB061	_2197	32	0	2	23	0	1	6
CB	GMDP	CB061	_2198	20	0	2	17	0	0	0
CB	GMDP	CB061	_2199	16	0	0	2	0	0	14
CB	CDP	CB061	_2200	18	0	0	18	0	0	0
CB	CDP	CB061	_2201	18	0	0	18	0	0	0
CB	CDP	CB061	_2202	15	0	0	15	0	0	0
CB	CDP	CB061	_2203	13	0	0	13	0	0	0
CB	CDP	CB061	_2204	19	0	0	19	0	0	0
CB	CDP	CB061	_2205	16	0	0	16	0	0	0
CB	CDP	CB061	_2206	13	0	0	13	0	0	0
CB	CDP	CB061	_2207	5	0	0	4	1	0	0
CB	CDP	CB061	_2208	9	0	0	9	0	0	0
CB	CDP	CB061	_2209	7	0	0	7	0	0	0
CB	CDP	CB061	_2210	5	0	0	5	0	0	0
CB	CDP	CB061	_2211	7	0	0	7	0	0	0
CB	CDP	CB061	_2212	3	0	0	3	0	0	0
CB	CDP	CB061	_2213	6	0	0	6	0	0	0
CB	CDP	CB061	_2214	3	0	0	3	0	0	0
CB	CDP	CB061	_2215	4	0	0	4	0	0	0
CB	CDP	CB061	_2216	3	0	0	0	0	3	0
CB	CDP	CB061	_2217	4	0	0	0	0	4	0
CB	CDP	CB061	_2218	3	0	0	0	0	3	0
CB	CDP	CB061	_2219	7	0	0	0	0	7	0
CB	MDP	CB048	_2220	30	0	30	0	0	0	0
CB	MDP	CB048	_2221	33	0	33	0	0	0	0
CB	MDP	CB048	_2222	33	0	33	0	0	0	0
CB	MDP	CB048	_2223	49	0	49	0	0	0	0
CB	MDP	CB048	_2224	29	0	29	0	0	0	0
CB	MDP	CB048	_2225	58	0	58	0	0	0	0
CB	MDP	CB048	_2226	12	0	12	0	0	0	0
CB	MDP	CB048	_2227	46	0	46	0	0	0	0
CB	MDP	CB048	_2228	25	0	25	0	0	0	0
CB	MDP	CB048	_2229	44	0	43	0	0	1	0
CB	MDP	CB048	_2230	11	0	11	0	0	0	0
CB	MDP	CB048	_2231	47	0	0	47	0	0	0
CB	MDP	CB048	_2232	15	0	0	13	2	0	0
CB	MDP	CB048	_2233	37	0	1	33	3	0	0
CB	MDP	CB048	_2234	154	0	1	153	0	0	0
CB	MDP	CB048	_2235	123	0	0	123	0	0	0
CB	MDP	CB048	_2236	17	0	2	5	7	3	0
CB	MDP	CB048	_2237	25	0	0	1	24	0	0
CB	MDP	CB048	_2238	20	0	1	0	0	19	0
CB	MDP	CB048	_2239	23	0	2	0	0	20	0
CB	MDP	CB048	_2240	17	0	0	1	0	16	0
CB	MDP	CB048	_2241	12	0	0	0	0	10	0
CB	MDP	CB048	_2242	10	0	0	0	0	10	0
CB	MDP	CB048	_2243	23	0	0	0	0	23	0
CB	MDP	CB048	_2244	39	1	2	0	0	32	0
CB	MDP	CB048	_2245	23	0	1	0	0	0	22
CB	CDP	CB048	_2246	29	1	1	27	0	0	0
CB	CDP	CB048	_2247	27	0	0	27	0	0	0
CB	CDP	CB048	_2248	13	0	0	13	0	0	0
CB	CDP	CB048	_2249	15	2	1	12	0	0	0
CB	CDP	CB048	_2250	19	0	0	18	0	1	0

CB	CDP	CB048	_2251	9	0	0	9	0	0	0
CB	CDP	CB048	_2252	4	0	0	4	0	0	0
CB	CDP	CB048	_2253	5	0	0	5	0	0	0
CB	CDP	CB048	_2254	7	0	0	7	0	0	0
CB	CDP	CB048	_2255	8	0	1	7	0	0	0
CB	CDP	CB048	_2256	4	0	0	0	0	4	0
CB	CDP	CB048	_2257	6	0	0	0	0	6	0
CB	CDP	CB048	_2258	3	0	0	0	0	3	0
CB	CDP	CB048	_2259	5	1	0	0	0	3	0
CB	CDP	CB048	_2260	3	0	0	0	0	3	0
CB	CDP	CB048	_2261	4	0	0	0	0	4	0

CB	Trace_CB215	HSC	Ancestor_002	470	0	19	48	14	54	335
CB	Trace_CB215	HSC	GDaughter_002	444	0	0	41	14	54	335
CB	Trace_CB215	HSC	GDaughter_002	26	0	19	7	0	0	0
CB	Trace_CB215	HSC	Ancestor_003	39954	1137	19163	10993	1312	1518	5831
CB	Trace_CB215	HSC	GDaughter_003	19830	787	12609	3256	657	393	2128
CB	Trace_CB215	HSC	GDaughter_003	11331	350	6101	3732	299	534	315
CB	Trace_CB215	HSC	GDaughter_003	4497	0	307	1734	253	346	1857
CB	Trace_CB215	HSC	GDaughter_003	4296	0	146	2271	103	245	1531
CB	Trace_CB215	HSC	Ancestor_004	648	8	178	163	18	40	241
CB	Trace_CB215	HSC	GDaughter_004	428	8	159	128	12	20	101
CB	Trace_CB215	HSC	GDaughter_004	124	0	10	35	6	8	65
CB	Trace_CB215	HSC	GDaughter_004	91	0	9	0	0	12	70
CB	Trace_CB215	HSC	GDaughter_004	5	0	0	0	0	0	5
CB	Trace_CB215	HSC	Ancestor_005	178	0	0	71	23	14	70
CB	Trace_CB215	HSC	GDaughter_005	155	0	0	71	0	14	70
CB	Trace_CB215	HSC	GDaughter_005	23	0	0	0	23	0	0
CB	Trace_CB215	HSC	Ancestor_006	287	13	122	52	16	11	73
CB	Trace_CB215	HSC	GDaughter_006	213	13	122	28	7	5	38
CB	Trace_CB215	HSC	GDaughter_006	65	0	0	24	0	6	35
CB	Trace_CB215	HSC	GDaughter_006	9	0	0	0	9	0	0
CB	Trace_CB215	HSC	Ancestor_007	2866	68	499	826	301	142	1030
CB	Trace_CB215	HSC	GDaughter_007	1282	48	499	349	83	68	235
CB	Trace_CB215	HSC	GDaughter_007	1199	20	0	400	161	57	561
CB	Trace_CB215	HSC	GDaughter_007	356	0	0	69	46	17	224
CB	Trace_CB215	HSC	GDaughter_007	29	0	0	8	11	0	10
CB	Trace_CB215	HSC	Ancestor_009	85019	7870	61326	14149	1560	87	27
CB	Trace_CB215	HSC	GDaughter_009	24806	3086	17018	4253	413	27	9
CB	Trace_CB215	HSC	GDaughter_009	28324	529	19559	7518	666	34	18
CB	Trace_CB215	HSC	GDaughter_009	31357	4166	24354	2339	472	26	0
CB	Trace_CB215	HSC	GDaughter_009	532	89	395	39	9	0	0
CB	Trace_CB215	HSC	Ancestor_010	22350	11412	10221	717	0	0	0
CB	Trace_CB215	HSC	GDaughter_010	16561	8480	7381	700	0	0	0
CB	Trace_CB215	HSC	GDaughter_010	5784	2932	2835	17	0	0	0
CB	Trace_CB215	HSC	Ancestor_011	26907	4931	21326	617	33	0	0
CB	Trace_CB215	HSC	GDaughter_011	6333	782	5066	452	33	0	0
CB	Trace_CB215	HSC	GDaughter_011	3116	440	2567	109	0	0	0
CB	Trace_CB215	HSC	GDaughter_011	11928	1707	10176	45	0	0	0
CB	Trace_CB215	HSC	GDaughter_011	5530	2002	3517	11	0	0	0
CB	Trace_CB215	HSC	Ancestor_013	9869	406	319	4248	3293	508	1095

CB	Trace_CB215	HSC	GDaughter_013	5762	406	263	1892	2075	392	734
CB	Trace_CB215	HSC	GDaughter_013	2418	0	47	1285	896	103	87
CB	Trace_CB215	HSC	GDaughter_013	1680	0	0	1071	322	13	274
CB	Trace_CB215	HSC	Ancestor_014	2247	1430	817	0	0	0	0
CB	Trace_CB215	HSC	GDaughter_014	2189	1393	796	0	0	0	0
CB	Trace_CB215	HSC	GDaughter_014	58	37	21	0	0	0	0
CB	Trace_CB215	HSC	Ancestor_015	6695	327	3962	2300	91	15	0
CB	Trace_CB215	HSC	GDaughter_015	3940	165	2012	1670	78	15	0
CB	Trace_CB215	HSC	GDaughter_015	527	22	244	248	13	0	0
CB	Trace_CB215	HSC	GDaughter_015	2122	61	1679	382	0	0	0
CB	Trace_CB215	HSC	GDaughter_015	106	79	27	0	0	0	0
CB	Trace_CB215	HSC	Ancestor_016	605	0	0	8	0	28	569
CB	Trace_CB215	HSC	GDaughter_016	128	0	0	0	0	8	120
CB	Trace_CB215	HSC	GDaughter_016	272	0	0	0	0	12	260
CB	Trace_CB215	HSC	GDaughter_016	36	0	0	0	0	8	28
CB	Trace_CB215	HSC	GDaughter_016	169	0	0	8	0	0	161
CB	Trace_CB215	HSC	Ancestor_017	7731	7715	16	0	0	0	0
CB	Trace_CB215	HSC	GDaughter_017	2378	2362	16	0	0	0	0
CB	Trace_CB215	HSC	GDaughter_017	2769	2769	0	0	0	0	0
CB	Trace_CB215	HSC	GDaughter_017	2568	2568	0	0	0	0	0
CB	Trace_CB215	HSC	GDaughter_017	16	16	0	0	0	0	0
CB	Trace_CB215	HSC	Ancestor_018	11	0	5	0	0	0	6
CB	Trace_CB215	HSC	GDaughter_018	6	0	0	0	0	0	6
CB	Trace_CB215	HSC	GDaughter_018	5	0	5	0	0	0	0
CB	Trace_CB215	HSC	Ancestor_022	1027	0	5	84	0	68	870
CB	Trace_CB215	HSC	GDaughter_022	510	0	5	55	0	45	405
CB	Trace_CB215	HSC	GDaughter_022	517	0	0	29	0	23	465
CB	Trace_CB215	HSC	Ancestor_023	851	0	0	402	0	36	413
CB	Trace_CB215	HSC	GDaughter_023	69	0	0	21	0	12	36
CB	Trace_CB215	HSC	GDaughter_023	464	0	0	244	0	18	202
CB	Trace_CB215	HSC	GDaughter_023	318	0	0	137	0	6	175
CB	Trace_CB215	HSC	Ancestor_024	34552	10723	23570	254	5	0	0
CB	Trace_CB215	HSC	GDaughter_024	3496	1446	2029	21	0	0	0
CB	Trace_CB215	HSC	GDaughter_024	17332	3068	14091	173	0	0	0
CB	Trace_CB215	HSC	GDaughter_024	13719	6209	7450	60	0	0	0
CB	Trace_CB210	HSC	Ancestor_027	193	0	0	0	0	0	193
CB	Trace_CB210	HSC	GDaughter_027	179	0	0	0	0	0	179
CB	Trace_CB210	HSC	GDaughter_027	14	0	0	0	0	0	14
CB	Trace_CB210	HSC	Ancestor_028	7413	470	119	2283	217	282	4042
CB	Trace_CB210	HSC	GDaughter_028	4081	50	17	1355	149	120	2390
CB	Trace_CB210	HSC	GDaughter_028	2896	0	86	928	68	162	1652
CB	Trace_CB210	HSC	GDaughter_028	375	359	16	0	0	0	0
CB	Trace_CB210	HSC	GDaughter_028	61	61	0	0	0	0	0
CB	Trace_CB210	HSC	Ancestor_029	1253	12	0	388	27	33	793
CB	Trace_CB210	HSC	GDaughter_029	1238	12	0	388	27	33	778
CB	Trace_CB210	HSC	GDaughter_029	15	0	0	0	0	0	15
CB	Trace_CB210	HSC	Ancestor_030	1383	0	345	1017	0	21	0
CB	Trace_CB210	HSC	GDaughter_030	827	0	113	693	0	21	0
CB	Trace_CB210	HSC	GDaughter_030	556	0	232	324	0	0	0
CB	Trace_CB210	HSC	Ancestor_031	2803	0	69	996	0	95	1643

CB	Trace_CB210	HSC	GDaughter_031	2213	0	69	939	0	95	1110
CB	Trace_CB210	HSC	GDaughter_031	568	0	0	35	0	0	533
CB	Trace_CB210	HSC	GDaughter_031	22	0	0	22	0	0	0
CB	Trace_CB210	HSC	Ancestor_032	478	0	0	0	0	0	478
CB	Trace_CB210	HSC	GDaughter_032	397	0	0	0	0	0	397
CB	Trace_CB210	HSC	GDaughter_032	81	0	0	0	0	0	81
CB	Trace_CB227	HSC	Ancestor_034	3192	54	38	718	2350	9	23
CB	Trace_CB227	HSC	GDaughter_034	197	0	0	50	147	0	0
CB	Trace_CB227	HSC	GDaughter_034	1231	12	24	345	850	0	0
CB	Trace_CB227	HSC	GDaughter_034	1764	42	14	323	1353	9	23
CB	Trace_CB227	HSC	Ancestor_035	4479	0	0	466	3158	535	320
CB	Trace_CB227	HSC	GDaughter_035	702	0	0	57	495	73	77
CB	Trace_CB227	HSC	GDaughter_035	2613	0	0	208	1972	320	113
CB	Trace_CB227	HSC	GDaughter_035	1164	0	0	201	691	142	130
CB	Trace_CB227	HSC	Ancestor_036	6485	2189	4284	12	0	0	0
CB	Trace_CB227	HSC	GDaughter_036	2643	622	2021	0	0	0	0
CB	Trace_CB227	HSC	GDaughter_036	2494	579	1903	12	0	0	0
CB	Trace_CB227	HSC	GDaughter_036	1348	988	360	0	0	0	0
CB	Trace_CB228	HSC	Ancestor_080	5074	0	0	956	20	1120	2978
CB	Trace_CB228	HSC	GDaughter_080	163	0	0	26	0	24	113
CB	Trace_CB228	HSC	GDaughter_080	4900	0	0	930	20	1096	2854
CB	Trace_CB228	HSC	GDaughter_080	11	0	0	0	0	0	11
CB	Trace_CB228	HSC	Ancestor_081	48	41	0	0	0	0	7
CB	Trace_CB228	HSC	GDaughter_081	16	16	0	0	0	0	0
CB	Trace_CB228	HSC	GDaughter_081	32	25	0	0	0	0	7
CB	Trace_CB228	HSC	Ancestor_086	593	123	7	23	370	0	70
CB	Trace_CB228	HSC	GDaughter_086	85	85	0	0	0	0	0
CB	Trace_CB228	HSC	GDaughter_086	127	16	0	0	111	0	0
CB	Trace_CB228	HSC	GDaughter_086	29	22	7	0	0	0	0
CB	Trace_CB228	HSC	GDaughter_086	352	0	0	23	259	0	70
CB	Trace_CB215	GMDP	Ancestor_001	355	32	269	54	0	0	0
CB	Trace_CB215	GMDP	GDaughter_001	334	32	255	47	0	0	0
CB	Trace_CB215	GMDP	GDaughter_001	21	0	14	7	0	0	0
CB	Trace_CB215	GMDP	Ancestor_002	314	0	223	91	0	0	0
CB	Trace_CB215	GMDP	GDaughter_002	38	0	17	21	0	0	0
CB	Trace_CB215	GMDP	GDaughter_002	206	0	206	0	0	0	0
CB	Trace_CB215	GMDP	GDaughter_002	70	0	0	70	0	0	0
CB	Trace_CB215	GMDP	Ancestor_004	285	0	285	0	0	0	0
CB	Trace_CB215	GMDP	GDaughter_004	32	0	32	0	0	0	0
CB	Trace_CB215	GMDP	GDaughter_004	15	0	15	0	0	0	0
CB	Trace_CB215	GMDP	GDaughter_004	172	0	172	0	0	0	0
CB	Trace_CB215	GMDP	GDaughter_004	66	0	66	0	0	0	0
CB	Trace_CB215	GMDP	Ancestor_005	19	0	0	6	0	0	13
CB	Trace_CB215	GMDP	GDaughter_005	13	0	0	6	0	0	7
CB	Trace_CB215	GMDP	GDaughter_005	6	0	0	0	0	0	6
CB	Trace_CB215	GMDP	Ancestor_006	146	0	7	139	0	0	0
CB	Trace_CB215	GMDP	GDaughter_006	66	0	7	59	0	0	0
CB	Trace_CB215	GMDP	GDaughter_006	7	0	0	7	0	0	0
CB	Trace_CB215	GMDP	GDaughter_006	60	0	0	60	0	0	0
CB	Trace_CB215	GMDP	GDaughter_006	13	0	0	13	0	0	0

CB	Trace_CB215	GMDP	Ancestor_009	195	0	29	153	13	0	0
CB	Trace_CB215	GMDP	GDaughter_009	163	0	21	136	6	0	0
CB	Trace_CB215	GMDP	GDaughter_009	24	0	0	17	7	0	0
CB	Trace_CB215	GMDP	GDaughter_009	8	0	8	0	0	0	0
CB	Trace_CB215	GMDP	Ancestor_010	757	0	300	448	0	0	9
CB	Trace_CB215	GMDP	GDaughter_010	297	0	153	135	0	0	9
CB	Trace_CB215	GMDP	GDaughter_010	295	0	75	220	0	0	0
CB	Trace_CB215	GMDP	GDaughter_010	155	0	62	93	0	0	0
CB	Trace_CB215	GMDP	GDaughter_010	10	0	10	0	0	0	0
CB	Trace_CB215	GMDP	Ancestor_011	118	0	0	110	0	0	8
CB	Trace_CB215	GMDP	GDaughter_011	41	0	0	33	0	0	8
CB	Trace_CB215	GMDP	GDaughter_011	7	0	0	7	0	0	0
CB	Trace_CB215	GMDP	GDaughter_011	6	0	0	6	0	0	0
CB	Trace_CB215	GMDP	GDaughter_011	64	0	0	64	0	0	0
CB	Trace_CB215	GMDP	Ancestor_013	146	0	0	98	48	0	0
CB	Trace_CB215	GMDP	GDaughter_013	11	0	0	11	0	0	0
CB	Trace_CB215	GMDP	GDaughter_013	55	0	0	55	0	0	0
CB	Trace_CB215	GMDP	GDaughter_013	48	0	0	0	48	0	0
CB	Trace_CB215	GMDP	GDaughter_013	32	0	0	32	0	0	0
CB	Trace_CB215	GMDP	Ancestor_015	300	21	44	235	0	0	0
CB	Trace_CB215	GMDP	GDaughter_015	135	0	28	107	0	0	0
CB	Trace_CB215	GMDP	GDaughter_015	95	0	16	79	0	0	0
CB	Trace_CB215	GMDP	GDaughter_015	49	0	0	49	0	0	0
CB	Trace_CB215	GMDP	GDaughter_015	21	21	0	0	0	0	0
CB	Trace_CB215	GMDP	Ancestor_017	904	880	19	5	0	0	0
CB	Trace_CB215	GMDP	GDaughter_017	303	284	19	0	0	0	0
CB	Trace_CB215	GMDP	GDaughter_017	596	596	0	0	0	0	0
CB	Trace_CB215	GMDP	Ancestor_019	57	0	0	14	43	0	0
CB	Trace_CB215	GMDP	GDaughter_019	50	0	0	14	36	0	0
CB	Trace_CB215	GMDP	GDaughter_019	7	0	0	0	7	0	0
CB	Trace_CB215	GMDP	Ancestor_020	53	0	53	0	0	0	0
CB	Trace_CB215	GMDP	GDaughter_020	31	0	31	0	0	0	0
CB	Trace_CB215	GMDP	GDaughter_020	16	0	16	0	0	0	0
CB	Trace_CB215	GMDP	Ancestor_021	29	0	0	7	22	0	0
CB	Trace_CB215	GMDP	GDaughter_021	12	0	0	7	5	0	0
CB	Trace_CB215	GMDP	GDaughter_021	11	0	0	0	11	0	0
CB	Trace_CB215	GMDP	GDaughter_021	6	0	0	0	6	0	0
CB	Trace_CB210	GMDP	Ancestor_023	359	51	265	43	0	0	0
CB	Trace_CB210	GMDP	GDaughter_023	349	51	265	33	0	0	0
CB	Trace_CB210	GMDP	GDaughter_023	10	0	0	10	0	0	0
CB	Trace_CB227	GMDP	Ancestor_024	811	0	376	435	0	0	0
CB	Trace_CB227	GMDP	GDaughter_024	142	0	16	126	0	0	0
CB	Trace_CB227	GMDP	GDaughter_024	170	0	47	123	0	0	0
CB	Trace_CB227	GMDP	GDaughter_024	184	0	146	38	0	0	0
CB	Trace_CB227	GMDP	GDaughter_024	315	0	167	148	0	0	0
CB	Trace_CB227	GMDP	Ancestor_026	400	0	0	400	0	0	0
CB	Trace_CB227	GMDP	GDaughter_026	178	0	0	178	0	0	0
CB	Trace_CB227	GMDP	GDaughter_026	64	0	0	64	0	0	0
CB	Trace_CB227	GMDP	GDaughter_026	158	0	0	158	0	0	0
CB	Trace_CB227	GMDP	Ancestor_028	186	0	186	0	0	0	0

CB	Trace_CB227	GMDP	GDaughter_028	81	0	81	0	0	0	0
CB	Trace_CB227	GMDP	GDaughter_028	105	0	105	0	0	0	0
CB	Trace_CB227	GMDP	Ancestor_029	95	0	0	95	0	0	0
CB	Trace_CB227	GMDP	GDaughter_029	83	0	0	83	0	0	0
CB	Trace_CB227	GMDP	GDaughter_029	12	0	0	12	0	0	0
CB	Trace_CB227	GMDP	Ancestor_030	150	0	150	0	0	0	0
CB	Trace_CB227	GMDP	GDaughter_030	114	0	114	0	0	0	0
CB	Trace_CB227	GMDP	GDaughter_030	36	0	36	0	0	0	0
CB	Trace_CB227	GMDP	Ancestor_032	684	77	607	0	0	0	0
CB	Trace_CB227	GMDP	GDaughter_032	126	0	126	0	0	0	0
CB	Trace_CB227	GMDP	GDaughter_032	284	0	284	0	0	0	0
CB	Trace_CB227	GMDP	GDaughter_032	31	31	0	0	0	0	0
CB	Trace_CB227	GMDP	GDaughter_032	243	46	197	0	0	0	0
CB	Trace_CB227	GMDP	Ancestor_033	148	7	95	46	0	0	0
CB	Trace_CB227	GMDP	GDaughter_033	35	0	0	35	0	0	0
CB	Trace_CB227	GMDP	GDaughter_033	63	0	52	11	0	0	0
CB	Trace_CB227	GMDP	GDaughter_033	43	0	43	0	0	0	0
CB	Trace_CB227	GMDP	Ancestor_034	47	0	0	47	0	0	0
CB	Trace_CB227	GMDP	GDaughter_034	25	0	0	25	0	0	0
CB	Trace_CB227	GMDP	GDaughter_034	22	0	0	22	0	0	0
CB	Trace_CB228	GMDP	Ancestor_039	139	34	105	0	0	0	0
CB	Trace_CB228	GMDP	GDaughter_039	10	10	0	0	0	0	0
CB	Trace_CB228	GMDP	GDaughter_039	79	24	55	0	0	0	0
CB	Trace_CB228	GMDP	GDaughter_039	50	0	50	0	0	0	0
CB	Trace_CB228	GMDP	Ancestor_041	2926	2267	659	0	0	0	0
CB	Trace_CB228	GMDP	GDaughter_041	2025	1366	659	0	0	0	0
CB	Trace_CB228	GMDP	GDaughter_041	766	766	0	0	0	0	0
CB	Trace_CB228	GMDP	GDaughter_041	135	135	0	0	0	0	0
CB	Trace_CB228	GMDP	Ancestor_042	103	0	0	94	9	0	0
CB	Trace_CB228	GMDP	GDaughter_042	41	0	0	41	0	0	0
CB	Trace_CB228	GMDP	GDaughter_042	33	0	0	24	9	0	0
CB	Trace_CB228	GMDP	GDaughter_042	29	0	0	29	0	0	0
CB	Trace_CB228	GMDP	Ancestor_043	36	36	0	0	0	0	0
CB	Trace_CB228	GMDP	GDaughter_043	20	20	0	0	0	0	0
CB	Trace_CB228	GMDP	GDaughter_043	16	16	0	0	0	0	0
CB	Trace_CB228	GMDP	Ancestor_046	108	108	0	0	0	0	0
CB	Trace_CB228	GMDP	GDaughter_046	72	72	0	0	0	0	0
CB	Trace_CB228	GMDP	GDaughter_046	36	36	0	0	0	0	0
CB	Trace_CB228	GMDP	Ancestor_049	239	0	0	48	0	32	159
CB	Trace_CB228	GMDP	GDaughter_049	56	0	0	0	0	9	47
CB	Trace_CB228	GMDP	GDaughter_049	83	0	0	28	0	12	43
CB	Trace_CB228	GMDP	GDaughter_049	100	0	0	20	0	11	69
CB	Trace_CB228	GMDP	Ancestor_054	780	0	414	366	0	0	0
CB	Trace_CB228	GMDP	GDaughter_054	192	0	192	0	0	0	0
CB	Trace_CB228	GMDP	GDaughter_054	207	0	189	18	0	0	0
CB	Trace_CB228	GMDP	GDaughter_054	184	0	33	151	0	0	0
CB	Trace_CB228	GMDP	GDaughter_054	197	0	0	197	0	0	0
CB	Trace_CB228	GMDP	Ancestor_055	74	74	0	0	0	0	0
CB	Trace_CB228	GMDP	GDaughter_055	16	16	0	0	0	0	0
CB	Trace_CB228	GMDP	GDaughter_055	15	15	0	0	0	0	0

CB	Trace_CB228	GMDP	GDaughter_055	23	23	0	0	0	0	0
CB	Trace_CB228	GMDP	GDaughter_055	11	11	0	0	0	0	0
CB	Trace_CB228	GMDP	Ancestor_056	174	0	0	138	36	0	0
CB	Trace_CB228	GMDP	GDaughter_056	12	0	0	0	12	0	0
CB	Trace_CB228	GMDP	GDaughter_056	103	0	0	103	0	0	0
CB	Trace_CB228	GMDP	GDaughter_056	35	0	0	35	0	0	0
CB	Trace_CB228	GMDP	GDaughter_056	24	0	0	0	24	0	0
CB	Trace_CB228	GMDP	Ancestor_057	191	174	0	0	17	0	0
CB	Trace_CB228	GMDP	GDaughter_057	104	104	0	0	0	0	0
CB	Trace_CB228	GMDP	GDaughter_057	70	70	0	0	0	0	0
CB	Trace_CB228	GMDP	GDaughter_057	17	0	0	0	17	0	0
CB	Trace_CB228	GMDP	Ancestor_058	39	39	0	0	0	0	0
CB	Trace_CB228	GMDP	GDaughter_058	19	19	0	0	0	0	0
CB	Trace_CB228	GMDP	GDaughter_058	13	13	0	0	0	0	0
CB	Trace_CB228	GMDP	GDaughter_058	7	7	0	0	0	0	0
CB	Trace_CB228	GMDP	Ancestor_059	399	399	0	0	0	0	0
CB	Trace_CB228	GMDP	GDaughter_059	105	105	0	0	0	0	0
CB	Trace_CB228	GMDP	GDaughter_059	45	45	0	0	0	0	0
CB	Trace_CB228	GMDP	GDaughter_059	137	137	0	0	0	0	0
CB	Trace_CB228	GMDP	GDaughter_059	112	112	0	0	0	0	0
CB	Trace_CB228	GMDP	Ancestor_060	966	0	387	579	0	0	0
CB	Trace_CB228	GMDP	GDaughter_060	67	0	0	67	0	0	0
CB	Trace_CB228	GMDP	GDaughter_060	269	0	65	204	0	0	0
CB	Trace_CB228	GMDP	GDaughter_060	135	0	0	135	0	0	0
CB	Trace_CB228	GMDP	GDaughter_060	495	0	322	173	0	0	0

JD culture

Donor	Progenitor	Sample	Ery	Mek	G	M	C	A	P	L	Total
CB245	HSCMPP	1: CB_HSCMPP	10	0	2	1143	223	102	42	116	1638
CB245	HSCMPP	8: CB_HSCMPP	13	41	90	4824	2824	91	1430	91	9404
CB245	HSCMPP	13: CB_HSCMP	8	82	14147	3324	136	52	34	47	17830
CB245	HSCMPP	28: CB_HSCMP	39	443	1319	9002	1653	298	252	43	13049
CB245	HSCMPP	34: CB_HSCMP	61	261	33	4039	4315	581	1465	36	10791
CB245	HSCMPP	53: CB_HSCMP	46	1411	9695	8080	778	348	71	29	20458
CB245	HSCMPP	69: CB_HSCMP	10	657	106	23496	3784	526	892	33	29504
CB245	HSCMPP	91: CB_HSCMP	69	402	5350	10438	775	8	117	97	17256
CB245	HSCMPP	95: CB_HSCMP	101	928	93	27345	2023	114	1220	107	31931
CB245	HSCMPP	10: CB_HSCMP	0	0	1	1	1	0	0	1	4
CB245	HSCMPP	103: CB_HSCM	22	2240	8	27496	243	11	166	57	30243
CB245	HSCMPP	12: CB_HSCMP	61	0	0	12	0	0	0	2	75
CB245	HSCMPP	121: CB_HSCM	28	11	14	8460	1713	33	227	167	10653
CB245	HSCMPP	14: CB_HSCMP	0	0	8	87	240	7	21	404	767
CB245	HSCMPP	15: CB_HSCMP	1	0	0	8	0	0	0	0	9
CB245	HSCMPP	130: CB_HSCM	150	1958	8	25379	658	18	116	15	28302
CB245	HSCMPP	141: CB_HSCM	18	446	88	2848	1283	596	285	271	5835
CB245	HSCMPP	193: CB_HSCM	9	699	123	17969	12170	695	4163	107	35935
CB245	HSCMPP	194: CB_HSCM	17	782	2713	10472	4611	1118	2787	269	22769
CB245	HSCMPP	207: CB_HSCM	8	874	563	9971	2469	462	734	30	15111
CB245	HSCMPP	21: CB_HSCMP	0	0	1	8	0	0	0	1	10
CB245	HSCMPP	214: CB_HSCM	27	535	90	7157	5690	333	1898	113	15843
CB245	HSCMPP	23: CB_HSCMP	16	0	3	89	127	45	16	108	404
CB245	HSCMPP	223: CB_HSCM	18	356	75	331	2691	141	291	197	4100
CB245	HSCMPP	237: CB_HSCM	24	1573	52	16965	1231	368	388	10	20611
CB245	HSCMPP	26: CB_HSCMP	1	0	8	154	154	39	16	104	476
CB245	HSCMPP	27: CB_HSCMP	1	0	1	5	0	0	0	1	8
CB245	HSCMPP	241: CB_HSCM	15	2614	14	26771	1423	64	415	13	31329
CB245	HSCMPP	278: CB_HSCM	37	1255	1345	4805	2095	465	110	41	10153
CB245	HSCMPP	5: CB_HSCMPP	12	217	26	4085	1101	93	64	6	5604
CB245	HSCMPP	16: CB_HSCMP	6	500	46	24942	1529	66	695	38	27822
CB245	HSCMPP	25: CB_HSCMP	538	1071	548	24148	70	6	13	51	26445
CB245	HSCMPP	38: CB_HSCMP	1	234	404	3402	223	235	215	23	4737
CB245	HSCMPP	47: CB_HSCMP	2	23	56	1848	2808	70	1553	239	6599
CB245	HSCMPP	61: CB_HSCMP	5	902	63	23201	5658	195	4248	52	34324
CB245	HSCMPP	72: CB_HSCMP	1	163	78	744	475	141	34	18	1654
CB245	HSCMPP	37: CB_HSCMP	0	0	1	16	38	16	3	68	142
CB245	HSCMPP	77: CB_HSCMP	4	439	77	3013	6161	1553	2636	70	13953
CB245	HSCMPP	82: CB_HSCMP	11	241	172	1532	64	19	25	4	2068
CB245	HSCMPP	83: CB_HSCMP	13	545	148	913	212	528	60	0	2419
CB245	HSCMPP	85: CB_HSCMP	26	1321	9	6976	985	3	87	37	9444
CB245	HSCMPP	107: CB_HSCM	5	592	60	355	3070	118	214	104	4518
CB245	HSCMPP	110: CB_HSCM	2	545	10	129	259	42	89	108	1184

CB245	HSCMPP	138: CB_HSCM	12	1338	24930	13755	149	6	18	8	40216
CB245	HSCMPP	140: CB_HSCM	7	1930	28	50732	1806	30	489	14	55036
CB245	HSCMPP	46: CB_HSCMP	0	0	1	3	0	0	0	0	4
CB245	HSCMPP	145: CB_HSCM	2	1303	863	4578	2729	210	306	46	10037
CB245	HSCMPP	48: CB_HSCMP	0	0	1	4	3	0	0	0	8
CB245	HSCMPP	152: CB_HSCM	5	1520	1613	7926	1553	305	474	20	13416
CB245	HSCMPP	50: CB_HSCMP	0	0	40	141	224	52	76	29	562
CB245	HSCMPP	156: CB_HSCM	2	1048	68	5817	4363	1217	1014	46	13575
CB245	HSCMPP	163: CB_HSCM	22	1009	16	17060	227	1	41	164	18540
CB245	HSCMPP	170: CB_HSCM	8	1185	357	17477	3431	188	1027	4	23677
CB245	HSCMPP	197: CB_HSCM	39	777	8	124	58	5	16	108	1135
CB245	HSCMPP	220: CB_HSCM	25	3333	26	30024	132	1	34	55	33630
CB245	HSCMPP	228: CB_HSCM	3646	1654	18244	8266	41	26	9	2	31888
CB245	HSCMPP	232: CB_HSCM	6	3177	93	24090	3887	130	3242	101	34726
CB245	HSCMPP	235: CB_HSCM	1	266	4549	1395	631	35	34	8	6919
CB245	HSCMPP	239: CB_HSCM	3	328	76	2921	1660	825	750	274	6837
CB245	HSCMPP	60: CB_HSCMP	0	7	698	632	278	18	41	9	1683
CB245	HSCMPP	243: CB_HSCM	6	979	125	6495	6168	1218	2659	62	17712
CB245	HSCMPP	244: CB_HSCM	2	929	112	12801	7300	101	4723	381	26349
CB245	HSCMPP	251: CB_HSCM	3	1372	86	7938	7744	1849	3348	147	22487
CB245	HSCMPP	255: CB_HSCM	2	191	14	207	308	79	26	169	996
CB245	HSCMPP	264: CB_HSCM	6	739	590	1035	3503	1063	1175	230	8341
CB245	HSCMPP	271: CB_HSCM	10	366	75	19	90	3	35	117	715
CB245	HSCMPP	9: CB_HSCMPP	1	66	5	70	333	95	37	291	898
CB245	HSCMPP	54: CB_HSCMP	1	298	38	262	288	5	46	162	1100
CB245	HSCMPP	57: CB_HSCMP	6	915	57	38906	494	16	446	3	40843
CB245	HSCMPP	79: CB_HSCMP	25	1040	5	29589	1938	2	100	12	32711
CB245	HSCMPP	71: CB_HSCMP	0	0	0	2	1	0	0	0	3
CB245	HSCMPP	124: CB_HSCM	22	610	21263	7114	19	4	2	96	29130
CB245	HSCMPP	134: CB_HSCM	4331	2541	16	46231	37	0	280	5	53441
CB245	HSCMPP	74: CB_HSCMP	1	0	20	80	862	39	34	138	1174
CB245	HSCMPP	137: CB_HSCM	1468	2257	16	51160	113	1	189	2	55206
CB245	HSCMPP	76: CB_HSCMP	6	0	0	10	0	0	0	0	16
CB245	HSCMPP	154: CB_HSCM	1753	2632	204	866	18	0	1	25	5499
CB245	HSCMPP	173: CB_HSCM	8	5003	28	30326	458	4	639	7	36473
CB245	HSCMPP	182: CB_HSCM	1	183	32	119	100	28	60	3	526
CB245	HSCMPP	184: CB_HSCM	35	1262	41	37068	414	4	33	1	38858
CB245	HSCMPP	258: CB_HSCM	1	518	11	39	45	0	18	64	696
CB245	HSCMPP	18: CB_HSCMP	2567	418	14075	23918	0	0	1	11	40990
CB245	HSCMPP	42: CB_HSCMP	12	169	2	23473	417	1	17	7	24098
CB245	HSCMPP	51: CB_HSCMP	45	288	5410	28878	0	0	4	15	34640
CB245	HSCMPP	52: CB_HSCMP	42	332	6	40658	89	0	3	8	41138
CB245	HSCMPP	68: CB_HSCMP	43	718	28883	14418	0	0	2	14	44078
CB245	HSCMPP	78: CB_HSCMP	3502	1076	71	76	7	2	3	8	4745
CB245	HSCMPP	97: CB_HSCMP	10	1589	2985	1672	7	0	0	13	6276

CB245	HSCMPP	122: CB_HSCM	47	1286	3027	43037	2	0	17	2	47418
CB245	HSCMPP	133: CB_HSCM	8586	664	656	594	8	0	1	3	10512
CB245	HSCMPP	147: CB_HSCM	3	170	7699	7121	18	5	4	34	15054
CB245	HSCMPP	167: CB_HSCM	96	475	7775	32586	0	0	8	2	40942
CB245	HSCMPP	172: CB_HSCM	0	52	3	67	37	3	23	35	220
CB245	HSCMPP	185: CB_HSCM	33	937	2201	4797	0	0	13	0	7981
CB245	HSCMPP	191: CB_HSCM	2447	410	11229	23263	0	0	12	0	37361
CB245	HSCMPP	199: CB_HSCM	31	953	2916	5521	0	0	12	3	9436
CB245	HSCMPP	202: CB_HSCM	2	694	5	56	224	30	0	13	1024
CB245	HSCMPP	205: CB_HSCM	2820	2013	988	211	2	1	1	17	6053
CB245	HSCMPP	225: CB_HSCM	1497	671	337	379	15	7	5	0	2911
CB245	HSCMPP	254: CB_HSCM	97	413	4610	29641	0	0	4	20	34785
CB245	HSCMPP	263: CB_HSCM	10	505	19	3086	87	0	0	0	3707
CB245	HSCMPP	7: CB_HSCMPP	570	853	1192	597	0	0	0	6	3218
CB245	HSCMPP	11: CB_HSCMP	2317	688	28	35	1	0	0	6	3075
CB245	HSCMPP	17: CB_HSCMP	2991	525	33556	7442	2	0	2	1	44519
CB245	HSCMPP	30: CB_HSCMP	8	319	22	32	0	0	0	2	383
CB245	HSCMPP	33: CB_HSCMP	1	382	344	8	0	0	0	10	745
CB245	HSCMPP	49: CB_HSCMP	20	777	41159	9846	0	0	0	1	51803
CB245	HSCMPP	58: CB_HSCMP	169	614	2731	21774	0	0	3	7	25298
CB245	HSCMPP	65: CB_HSCMP	1670	725	4837	35874	0	0	0	4	43110
CB245	HSCMPP	73: CB_HSCMP	323	453	471	452	0	0	1	6	1706
CB245	HSCMPP	75: CB_HSCMP	2987	1223	20	53	1	0	0	2	4286
CB245	HSCMPP	81: CB_HSCMP	25	479	1579	5530	5	1	4	3	7626
CB245	HSCMPP	93: CB_HSCMP	10	1007	3897	2674	1	0	3	3	7595
CB245	HSCMPP	98: CB_HSCMP	1330	1520	781	18157	4	0	7	6	21805
CB245	HSCMPP	119: CB_HSCM	5122	1947	11	55	0	0	0	3	7138
CB245	HSCMPP	123: CB_HSCM	2356	3084	19984	4200	0	0	0	5	29629
CB245	HSCMPP	126: CB_HSCM	4670	768	2540	2297	0	0	0	2	10277
CB245	HSCMPP	129: CB_HSCM	3	993	18	12	0	0	0	26	1052
CB245	HSCMPP	135: CB_HSCM	20	809	26668	11127	0	0	0	1	38625
CB245	HSCMPP	143: CB_HSCM	26	1430	31075	13210	0	0	0	3	45744
CB245	HSCMPP	146: CB_HSCM	42	326	4	1505	8	0	1	2	1888
CB245	HSCMPP	149: CB_HSCM	8	1190	46589	8864	0	0	2	4	56657
CB245	HSCMPP	150: CB_HSCM	3	1531	105	289	54	0	0	3	1985
CB245	HSCMPP	158: CB_HSCM	13287	996	21713	17382	0	0	1	1	53380
CB245	HSCMPP	159: CB_HSCM	3766	789	43	90	0	0	0	1	4689
CB245	HSCMPP	160: CB_HSCM	12	1097	8	74	0	0	0	0	1191
CB245	HSCMPP	161: CB_HSCM	15	1286	42812	2590	0	0	0	5	46708
CB245	HSCMPP	162: CB_HSCM	27	947	13266	23270	0	0	6	4	37520
CB245	HSCMPP	166: CB_HSCM	5080	2185	10350	406	0	0	5	3	18029
CB245	HSCMPP	179: CB_HSCM	30	78	5	9780	76	0	0	0	9969
CB245	HSCMPP	180: CB_HSCM	2166	2241	19	159	0	0	0	0	4585
CB245	HSCMPP	192: CB_HSCM	33	763	4380	9244	0	0	0	0	14420
CB245	HSCMPP	195: CB_HSCM	2084	995	11505	14222	0	0	7	3	28816

CB245	HSCMPP	196: CB_HSCM	4560	3319	18	68	0	0	0	3	7968
CB245	HSCMPP	200: CB_HSCM	1966	1856	8448	3010	1	0	1	1	15283
CB245	HSCMPP	216: CB_HSCM	11	855	24910	24348	1	0	2	6	50133
CB245	HSCMPP	217: CB_HSCM	1146	383	52607	2056	0	0	3	5	56200
CB245	HSCMPP	218: CB_HSCM	11	1286	30031	9310	4	0	6	6	40654
CB245	HSCMPP	221: CB_HSCM	3058	281	42340	7690	0	0	4	2	53375
CB245	HSCMPP	222: CB_HSCM	3116	1689	26	32	0	0	0	1	4864
CB245	HSCMPP	226: CB_HSCM	2429	603	36	24	4	0	0	1	3097
CB245	HSCMPP	231: CB_HSCM	286	908	43412	13712	0	0	1	0	58319
CB245	HSCMPP	257: CB_HSCM	201	491	36	2888	1	0	1	1	3619
CB245	HSCMPP	259: CB_HSCM	3761	1664	16	54	0	0	0	1	5496
CB245	HSCMPP	261: CB_HSCM	5851	2847	23	20	0	0	0	2	8743
CB245	HSCMPP	270: CB_HSCM	3506	807	33263	4566	0	0	3	1	42146
CB245	HSCMPP	273: CB_HSCM	3323	1638	29	32	0	0	0	0	5022
CB245	HSCMPP	279: CB_HSCM	30	914	819	1077	1	0	0	2	2843
CB245	HSCMPP	280: CB_HSCM	3379	3397	10	74	0	0	0	6	6866
CB245	HSCMPP	281: CB_HSCM	705	3051	1152	8667	1	0	7	4	13587
CB245	HSCMPP	282: CB_HSCM	876	1275	127	3296	0	1	2	1	5578
CB245	HSCMPP	4: CB_HSCMPP	7	27	50	541	0	0	1	0	626
CB245	HSCMPP	19: CB_HSCMP	8	22	5	22	0	0	0	0	57
CB245	HSCMPP	29: CB_HSCMP	2	693	22	33	3	0	0	0	753
CB245	HSCMPP	41: CB_HSCMP	2	250	0	751	54	0	0	4	1061
CB245	HSCMPP	55: CB_HSCMP	3	354	26340	6889	0	0	0	6	33592
CB245	HSCMPP	56: CB_HSCMP	6	677	17037	23863	0	0	0	5	41588
CB245	HSCMPP	59: CB_HSCMP	0	217	15	51	0	0	0	0	283
CB245	HSCMPP	67: CB_HSCMP	2	1814	9	18	0	0	0	3	1846
CB245	HSCMPP	70: CB_HSCMP	1	98	18	52	4	0	1	0	174
CB245	HSCMPP	87: CB_HSCMP	9	1190	0	9	0	0	0	0	1208
CB245	HSCMPP	89: CB_HSCMP	1	1692	241	26	1	0	0	0	1961
CB245	HSCMPP	102: CB_HSCM	3099	3155	4	40	0	0	0	0	6298
CB245	HSCMPP	106: CB_HSCM	1890	811	0	24	0	0	0	0	2725
CB245	HSCMPP	115: CB_HSCM	462	1020	1	192	0	0	1	5	1681
CB245	HSCMPP	120: CB_HSCM	20	146	0	15	0	0	0	2	183
CB245	HSCMPP	125: CB_HSCM	2	558	13	13	0	0	0	1	587
CB245	HSCMPP	128: CB_HSCM	0	944	1951	12	0	0	0	0	2907
CB245	HSCMPP	136: CB_HSCM	1	784	18	22	0	0	0	1	826
CB245	HSCMPP	139: CB_HSCM	1	573	17	28	0	0	0	0	619
CB245	HSCMPP	144: CB_HSCM	1	203	28	28	0	0	0	0	260
CB245	HSCMPP	148: CB_HSCM	2	525	13	24	0	0	0	0	564
CB245	HSCMPP	155: CB_HSCM	4	1107	55	72	0	1	0	0	1239
CB245	HSCMPP	168: CB_HSCM	0	491	17112	162	1	0	3	3	17772
CB245	HSCMPP	175: CB_HSCM	8	796	3	12885	0	0	0	7	13699
CB245	HSCMPP	178: CB_HSCM	5	175	38	230	0	0	0	0	448
CB245	HSCMPP	190: CB_HSCM	2061	362	4	8	0	0	1	0	2436
CB245	HSCMPP	203: CB_HSCM	781	379	5	20	0	0	0	0	1185

CB245	HSCMPP	204: CB_HSCM	17	517	5	9	0	0	0	0	548
CB245	HSCMPP	209: CB_HSCM	0	1033	24871	84	0	0	0	0	25988
CB245	HSCMPP	219: CB_HSCM	0	380	18	11	2	1	0	1	413
CB245	HSCMPP	229: CB_HSCM	2	558	48	13	1	1	0	0	623
CB245	HSCMPP	230: CB_HSCM	1	1045	17	17	0	0	0	0	1080
CB245	HSCMPP	233: CB_HSCM	4	603	8	23	6	0	1	1	646
CB245	HSCMPP	240: CB_HSCM	3	492	22	25	1	1	0	0	544
CB245	HSCMPP	242: CB_HSCM	204	278	2	214	1	0	0	4	703
CB245	HSCMPP	252: CB_HSCM	0	188	10	14	4	0	1	1	218
CB245	HSCMPP	253: CB_HSCM	1	669	13	8	1	0	0	0	692
CB245	HSCMPP	256: CB_HSCM	6	1195	48814	7520	1	0	0	0	57536
CB245	HSCMPP	260: CB_HSCM	22	355	4	10	1	0	0	0	392
CB245	HSCMPP	262: CB_HSCM	8	283	5	11	2	0	0	0	309
CB245	HSCMPP	267: CB_HSCM	8715	5453	7	64	3	0	0	1	14243
CB245	HSCMPP	268: CB_HSCM	4	396	27	25	1	0	0	0	453
CB245	HSCMPP	275: CB_HSCM	3	215	15	23	0	0	0	0	256
CB245	HSCMPP	276: CB_HSCM	0	287	30	17	0	0	0	1	335
CB245	HSCMPP	277: CB_HSCM	0	170	21	17	5	1	2	0	216
CB245	HSCMPP	2: CB_HSCMPP	0	85	0	10	0	0	0	0	95
CB245	HSCMPP	6: CB_HSCMPP	0	163	0	18	5	1	0	2	189
CB245	HSCMPP	31: CB_HSCMP	0	561	0	10	0	0	0	0	571
CB245	HSCMPP	35: CB_HSCMP	3	80	0	12	2	1	0	0	98
CB245	HSCMPP	39: CB_HSCMP	0	238	1	10	0	0	1	0	250
CB245	HSCMPP	40: CB_HSCMP	618	241	0	4	0	0	0	0	863
CB245	HSCMPP	43: CB_HSCMP	0	266	0	22	0	0	0	2	290
CB245	HSCMPP	44: CB_HSCMP	0	69	1	8	0	0	0	0	78
CB245	HSCMPP	45: CB_HSCMP	0	200	1	16	0	0	0	0	217
CB245	HSCMPP	62: CB_HSCMP	1	280	0	21	1	1	2	1	307
CB245	HSCMPP	63: CB_HSCMP	0	358	0	17	0	0	0	2	377
CB245	HSCMPP	64: CB_HSCMP	0	79	0	13	1	0	0	1	94
CB245	HSCMPP	66: CB_HSCMP	6	733	6	34	0	0	0	1	780
CB245	HSCMPP	80: CB_HSCMP	0	581	7	34	2	0	0	1	625
CB245	HSCMPP	84: CB_HSCMP	1	1240	1	14	2	1	0	0	1259
CB245	HSCMPP	86: CB_HSCMP	8	987	1	7	2	0	3	0	1008
CB245	HSCMPP	88: CB_HSCMP	2	1344	6	135	0	0	0	3	1490
CB245	HSCMPP	92: CB_HSCMP	0	369	5	14	2	0	1	0	391
CB245	HSCMPP	96: CB_HSCMP	0	532	1	22	3	0	0	0	558
CB245	HSCMPP	99: CB_HSCMP	1	758	1	24	0	0	0	0	784
CB245	HSCMPP	100: CB_HSCM	0	1004	2	8	0	0	0	2	1016
CB245	HSCMPP	101: CB_HSCM	0	718	2	13	0	0	0	0	733
CB245	HSCMPP	104: CB_HSCM	1	423	2	18	0	0	0	0	444
CB245	HSCMPP	108: CB_HSCM	0	280	2	8	3	0	0	0	293
CB245	HSCMPP	109: CB_HSCM	1	1660	2	19	0	0	0	0	1682
CB245	HSCMPP	111: CB_HSCM	0	900	1	13	0	0	1	0	915
CB245	HSCMPP	113: CB_HSCM	0	1411	1	14	0	0	0	0	1426

CB245	HSCMPP	117: CB_HSCM	0	1679	2	24	0	0	1	0	1706
CB245	HSCMPP	118: CB_HSCM	1	339	0	10	0	0	1	2	353
CB245	HSCMPP	127: CB_HSCM	4	518	2	8	1	0	0	1	534
CB245	HSCMPP	131: CB_HSCM	5	386	1	28	0	0	1	0	421
CB245	HSCMPP	132: CB_HSCM	0	2141	0	24	0	0	0	0	2165
CB245	HSCMPP	142: CB_HSCM	0	677	0	29	0	0	1	0	707
CB245	HSCMPP	151: CB_HSCM	0	1275	4	39	0	0	0	0	1318
CB245	HSCMPP	153: CB_HSCM	1	1106	4	21	4	0	0	0	1136
CB245	HSCMPP	157: CB_HSCM	1	565	4	10	7	0	3	0	590
CB245	HSCMPP	164: CB_HSCM	1	636	4	30	0	0	0	1	672
CB245	HSCMPP	165: CB_HSCM	1	954	3	12	0	0	0	1	971
CB245	HSCMPP	169: CB_HSCM	2	222	10	6	0	0	0	0	240
CB245	HSCMPP	171: CB_HSCM	1	393	1	27	1	0	0	2	425
CB245	HSCMPP	174: CB_HSCM	1	754	2	81	4	0	2	0	844
CB245	HSCMPP	176: CB_HSCM	1	1014	0	33	0	0	0	0	1048
CB245	HSCMPP	181: CB_HSCM	8	513	2	7	0	0	1	0	531
CB245	HSCMPP	186: CB_HSCM	0	587	2	23	0	0	0	0	612
CB245	HSCMPP	187: CB_HSCM	1	250	0	15	0	0	0	1	267
CB245	HSCMPP	188: CB_HSCM	0	237	2	16	0	0	0	0	255
CB245	HSCMPP	198: CB_HSCM	3	1648	1	8	0	0	0	1	1661
CB245	HSCMPP	206: CB_HSCM	3	1207	5	12	2	0	1	1	1231
CB245	HSCMPP	208: CB_HSCM	0	1503	2	29	2	0	1	0	1537
CB245	HSCMPP	210: CB_HSCM	0	161	15	6	0	0	0	0	182
CB245	HSCMPP	211: CB_HSCM	0	559	2	13	0	0	0	1	575
CB245	HSCMPP	227: CB_HSCM	15	313	3	7	0	0	0	1	339
CB245	HSCMPP	234: CB_HSCM	3	194	2	11	0	0	0	0	210
CB245	HSCMPP	238: CB_HSCM	0	331	2	14	1	0	0	0	348
CB245	HSCMPP	245: CB_HSCM	3	51	6	22	5	0	6	1	94
CB245	HSCMPP	246: CB_HSCM	0	357	1	10	0	0	0	0	368
CB245	HSCMPP	247: CB_HSCM	1	576	1	15	1	0	0	2	596
CB245	HSCMPP	248: CB_HSCM	0	963	0	15	0	0	0	3	981
CB245	HSCMPP	249: CB_HSCM	0	247	1	9	0	0	0	0	257
CB245	HSCMPP	265: CB_HSCM	1	1033	7	14	3	0	2	0	1060
CB245	HSCMPP	266: CB_HSCM	0	561	4	22	1	0	0	0	588
CB245	HSCMPP	269: CB_HSCM	2	1372	3	23	0	0	1	0	1401
CB245	HSCMPP	272: CB_HSCM	2	849	4	12	0	0	0	0	867
CB245	HSCMPP	274: CB_HSCM	4	219	2	8	1	0	0	0	234
CB245	HSCMPP	283: CB_HSCM	2	621	1	10	0	0	1	0	635
CB245	HSCMPP	284: CB_HSCM	0	402	1	9	0	0	0	0	412
CB245	HSCMPP	3: CB_HSCMPP	0	234	0	2	0	0	0	0	236
CB245	HSCMPP	20: CB_HSCMP	2	104	0	6	0	0	0	0	112
CB245	HSCMPP	22: CB_HSCMP	0	460	1	4	0	2	0	0	467
CB245	HSCMPP	24: CB_HSCMP	0	124	0	6	0	0	0	0	130
CB245	HSCMPP	32: CB_HSCMP	1	628	0	7	0	0	0	2	638
CB245	HSCMPP	36: CB_HSCMP	1	301	0	5	0	0	0	0	307

CB245	HSCMPP	90: CB_HSCMP	4	418	1	7	0	0	0	0	430
CB245	HSCMPP	94: CB_HSCMP	3	375	1	4	0	0	0	0	383
CB245	HSCMPP	105: CB_HSCM	0	488	0	6	0	0	1	0	495
CB245	HSCMPP	112: CB_HSCM	0	539	2	7	0	0	0	1	549
CB245	HSCMPP	114: CB_HSCM	1	567	1	7	0	0	0	0	576
CB245	HSCMPP	116: CB_HSCM	0	587	1	7	0	0	0	2	597
CB245	HSCMPP	177: CB_HSCM	0	369	2	4	0	0	0	0	375
CB245	HSCMPP	183: CB_HSCM	0	163	0	5	0	0	0	0	168
CB245	HSCMPP	189: CB_HSCM	0	840	1	7	0	0	2	0	850
CB245	HSCMPP	201: CB_HSCM	1	363	5	6	0	0	0	0	375
CB245	HSCMPP	212: CB_HSCM	2	27	5	6	0	0	0	0	40
CB245	HSCMPP	213: CB_HSCM	0	442	2	4	0	0	0	0	448
CB245	HSCMPP	215: CB_HSCM	2	318	1	6	5	0	0	0	332
CB245	HSCMPP	224: CB_HSCM	1	470	5	6	1	0	0	2	485
CB245	HSCMPP	236: CB_HSCM	0	453	1	6	0	0	0	0	460
CB245	HSCMPP	250: CB_HSCM	2	522	3	5	0	0	1	0	533
CB245	HSCMPP	285: CB_HSCM	3	1270	1	7	0	0	0	0	1281
